# Differential Effects of the Hormonal and Copper Intrauterine Device **on the Endometrial Transcriptome**

**DOI:** 10.1038/s41598-020-63798-8

**Published:** 2020-04-23

**Authors:** Karen Smith-McCune, Reuben Thomas, Sarah Averbach, Dominika Seidman, Margaret Takeda, Sahar Houshdaran, Linda C. Giudice

**Affiliations:** 1 Department of Obstetrics, Gynecology and Reproductive Sciences, University of California San Francisco, San Francisco, CA USA; 2 Gladstone Institutes, San Francisco, CA USA; 3 Present Address: Department of Obstetrics, Gynecology and Reproductive Sciences, University of California San Diego, San Diego, CA USA

**Keywords:** Hormonal therapies, Molecular medicine

## Abstract

The contraceptive effectiveness of intrauterine devices (IUDs) has been attributed in part to a foreign body reaction in the endometrium. We performed this study to better understand mechanisms of action of contraceptives of by studying their effects on endometrial and cervical transcriptomes. We collected endometrial and cervical biopsies from women using the levonorgestrel-releasing intrauterine system (LNG-IUS, n = 11), copper intrauterine device (cu-IUD, n = 13) or levonorgestrel-containing combined oral contraceptives (COC, n = 12), and from women not using contraceptives (control group, n = 11). Transcriptional profiling was performed with Affymetrix arrays, Principal Component Analysis and the bioconductor package limma. In endometrial samples from cu-IUD users, there were no genes with statistically significant differential expression compared to controls. In LNG-IUS users, 2509 genes were differentially expressed and mapped predominantly onto immune and inflammatory pathways. The cervical samples showed no statistically significant differential gene expression compared to controls. Hormonal and copper IUDs have significantly different effects on the endometrial transcriptome, with the LNG-IUS transcriptome showing pronounced inflammation and immune activation compared to controls whereas the cu-IUD transcriptome was indistinguishable from luteal phase endometrium. These findings argue against a foreign body reaction as a common mechanism of action of IUDs.

## Introduction

The intrauterine device (IUD) is a popular and effective form of contraception used by approximately 14% of women globally^1^. Commonly used IUDs include a hormonal IUD that releases levonorgestrel (LNG-IUS), and a non-hormonal copper IUD (cu-IUD) that releases copper ions. The contraceptive efficacy of IUDs has been attributed in part to their effects as foreign bodies that induce local inflammation; in addition, the cu-IUD and LNG-IUS have direct toxic effects on sperm and the LNG-IUS also causes endometrial atrophy and alterations in cervical mucus rendering it unfavorable to sperm penetration^2–5^.

We recently studied the local immune microenvironment in the upper female reproductive tract of LNG-IUS users compared to mid-luteal samples from women not using hormonal or intrauterine contraceptives^6,7^. We found that the endometrial transcriptome from LNG-IUS users was associated with pronounced signals of inflammation consistent with a foreign body reaction^7^. These findings could be due to the presence of a foreign body (the IUD), the effect of LNG released by the IUD, or both. The purpose of this study was to compare the independent and combined effects of LNG exposure and IUD exposure on the transcriptome of the upper female reproductive tract, in order to better understand potential mechanisms of contraceptive action by these different methods.

## Methods

### Study design

This was a cross-sectional study comparing transcriptomes from the endometrium or cervical transformation zone (TZ) from samples donated by 4 groups of women using: no hormonal or intrauterine contraception (controls), cu-IUD (Paragard T 380 A, Cooper Surgical, Trumbull CT), LNG-IUS (Mirena, Bayer Healthcare Pharmaceuticals Inc, Finland) or LNG-containing COC. The UCSF Human Research Protection Program & IRB approved the study protocol, recruiting and consent materials; all procedures were performed in accordance with these regulations.

### Study procedures

Methods for the recruitment, screening and sample collection are described in detail elsewhere^8^; participants whose samples contributed to this study had sufficient amounts of appropriate endometrial and/or cervical tissue. Briefly, healthy women volunteers age 18–45 years were recruited from the San Francisco Bay Area. Participants in the COC group had to be using a cyclic 28-day pill pack of ethinyl estrogen plus LNG containing either 0.10 or 0.15 mg of LNG per tablet. Participants in the control and cu-IUD groups had to have regular periods every 21–35 days. Exclusion criteria included hysterectomy, breast-feeding, being within 6 months of parturition, having abnormal cervical cytology in the past year, and use of systemic corticosteroids or immune‐modulating therapies. At the screening visit, written informed consent was obtained from all participants, urine was tested for pregnancy, *Chlamydia trachomatis* and *Neisseria gonorrheae*, and blood was tested for HIV serology; a positive result or clinical evidence of vaginitis, vaginosis or pelvic inflammatory disease resulted in exclusion. At the second study visit, participants were given kits and instructions for urine testing for luteinizing hormone (LH) (ClearBlue Ovulation Test Digital, Proctor and Gamble, Cincinnati, OH). Women in the control and cu-IUD groups underwent biopsies 7 to 11 days after a positive LH test result. Women using the LNG-IUS underwent biopsies 7 to 11 days after a positive LH test or at their convenience after 2 months with no positive result, whichever came first. COC users underwent biopsies on day 12–16 of their pill pack.

For sample collection, the posterior vaginal fornix was swabbed with Q-tips for measurement of pH and prostate specific antigen (Abacus Diagnostics, West Hills, CA), a marker of recent vaginal intercourse; pH > 6.0 or positive PSA test led to exclusion of the sample from analysis. Endometrial biopsy was obtained with a 3 mm biopsy cannula (Softflex Endometrial Biopsy Cannula, Integra Miltex, York PA) and tissue was collected with 1 or 2 passes. A biopsy with Tischler forceps was performed at the cervical TZ, identified as the junction between Lugol’s staining and non-staining epithelium; if the TZ was not seen, the biopsy was obtained with one of the biopsy prongs inside the os. Blood was collected for measurement of plasma progesterone level. Endometrial and cervical biopsies were snap frozen and stored at −80 degrees until analysis.

### RNA Extraction and Whole Genome Microarrays

Biopsy samples were minced into small fragments and total RNA was extracted and DNase-treated using the NuceloSpin RNA II Kit (Marcherey-Nagel Inc, Bethlehem, PA). RNA quality was assessed using Bioanalyzer 2100 (Agilent Technologies, Santa Clara, CA) and quantity and purity determined with a NanoDrop Spectrometer. The NuGen Pico V2, based on Ribo-SPIA technology, was used for amplification, fragmentation and biotin-labelling and the labeled cDNA was hybridized to Human Gene 1.0 microarrays (Affymetrix, Santa Clara, CA) at the UCSF Gladstone Institutes Genomics Core. The signal intensity fluorescent images produced during the Affymetrix GeneChip hybridizations were read using the Affymetrix Model 3000 Scanner and converted into GeneChip probe results files (cel) using Command and Expression Console software (Affymetrix).

### Gene expression data processing and statistical analysis

The analyses were performed separately comparing each of the contraceptive groups with the control group for the cervical and the endometrial samples. The raw cel files were read using the *read.celfiles* function that is part of the oligo package^9^ in bioconductor^10^. The probes were matched to their corresponding gene symbols and ensembl ids using the annotate^11^ and hugene10sttranscriptcluster.db^12^ packages. The expression of all genes were normalized across all the samples in the chosen contraceptive and control group using the Robust Multi-array Average (RMA) procedure^13^. The top 500 most variable (across all samples) genes were chosen using these normalized data. The data corresponding to these 500 genes were used to perform Principal Component Analyses (PCA) using the *prcomp* function in R^14^. The association of the expression of genes with the particular contraceptive use was estimated using the bioconductor package limma^15^. Due to potential confounding due to the age of the subjects, age was adjusted as a linear term in the (limma) models associating expression of each gene with contraceptive use. The p-values reported by limma are adjusted for multiple testing using the False Discovery Rate (FDR) procedure^16^. The raw cel files discussed in this publication have been deposited in NCBI’s Gene Expression Omnibus^17^ and are accessible through GEO Series accession number GSE137765.

### Gene ontology and pathway analysis

The list of differentially expressed genes with adjusted p ≤ 0.05 were entered into EnrichR, a gene list enrichment analysis tool^18,19^ and the Pathways analysis produced by Reactome 2016^20^. The outputs displaying pathways ranked by combined score, a calculation based on the p-value and the Z score [log(p)•z], were chosen for presentation here.

## Results

### Characteristics of study participants

Table 1 demonstrates the demographic characteristics of participants who donated endometrial samples to the study. The control group was significantly older than the IUD and COC users, hence the gene expression association analyses presented below are adjusted for age as described above. The groups did not otherwise differ with respect to race, ethnicity, education, or smoking history. For endometrial samples, the median progesterone level (ng/ml) in the control group was 10.8 (range 5.8–18.6) and in the cu-IUD group was 11.2 (5.7–27.8), confirming that the participants in these groups were in the luteal phase. The median progesterone level (ng/ml) in the LNG-IUS group was 3.0 (<0.5–14.3) and 4 of 11 (36%) had values >2 indicating ovulation. None of the participants in the COC group had measurable progesterone levels, consistent with the known suppression of ovulation by COCs. The median length of contraceptive exposure was 25 (range 9–42) months for COC, 17 (7–36) months for LNG-IUS and 23 (6–45) months for cu-IUD.

**Table 1:** Demographic characteristics of participants who contributed endometrial samples to the study.

	Control (n = 11)	COC (n = 12)	LNG-IUS (n = 11)	Cu-IUD (n = 13)	P value
Age, median (min, max)	32(25,46)	24(19,33)	26(20,41)	28(22,33)	0.002*
Race, Number (%)					
Asian	1(9.1)	4(33.3)	1(9.1)	3(23.1)	0.99^§^
Black/African American	5(45.4)	1(8.3)	0	0	
Other/2 or more	1(9.1)	2(16.7)	1(9.1)	1(7.7)	
Unknown	0	1(8.3)	1(9.1)	1(7.7)	
White	4(36.4)	4(33.3)	8(72.7)	8(61.5)	
Ethnicity, Number (%)					0.55^§^
Latino	3(27.3)	1(8.3)	3(27.3)	4(30.8)	
Non-Latino	8(72.7)	11(91.7)	8(72.7)	9(69.2)	
Unknown	0	0	0	0	
Education, Number (%)					0.77^§^
Some high school or college	3(27.3)	4(33.3)	3(27.3)	2(15.4)	
Finished college or					
graduate school	8(72.7)	8(66.7)	8(72.7)	11(84.6)	
Smoking ever, Number (%)	3(27.3)	0	2(18.2)	1(7.7)	0.18^§^

COC combined oral contraceptive; LNG-IUS levonorgestrel-releasing intrauterine system; cu-IUD copper IUD.

* ANOVA. ^§^Fisher’s exact test.

### Effects of contraceptives on gene expression

**Figure 1:**
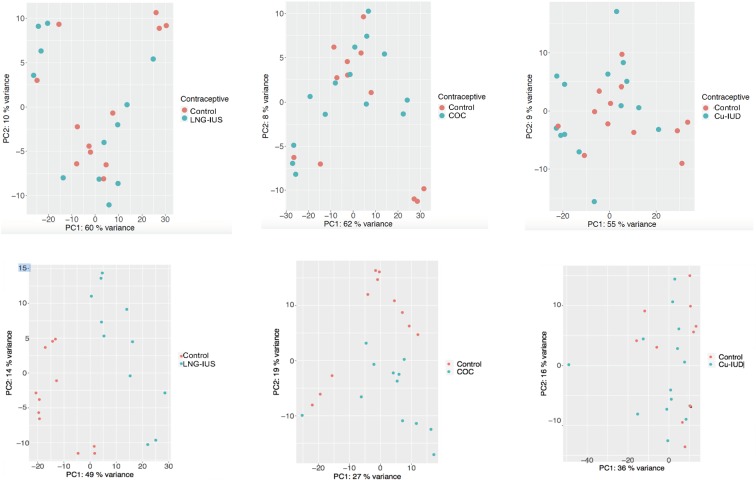
Principal component analysis. After gene expression was normalized across all the samples in the chosen contraceptive and control group using the Robust Multi-array Average (RMA) procedure^13^, the data corresponding to the top 500 most variable genes were used to perform Principal Component Analyses (PCA) using the *prcomp* function in R^14^ for samples from cervix (top) and endometrium (bottom).

Principal component analysis (PCA) of each of the contraceptive groups compared to the control group is shown in Fig. 1. The cervical PCA plots demonstrate minimal differentiation between control samples and each of the contraceptive groups (Fig. 1top). In statistical analysis of gene expression using an adjusted p ≤ 0.05 as the threshold, no genes were significantly differentially expressed in cervical samples from women using LNG-IUS, COC or cu-IUD compared to controls. The PCA plot for endometrial samples shows considerable sample separation for LNG-IUS versus control and is suggestive of separation for COC versus control (Fig. 1bottom); these comparisons are further analyzed below. For cu-IUD users, the PCA plot shows minimal differentiation with controls, and no genes had statistically significant differential expression in cu-IUD users compared to controls.

### Effect of LNG-IUS on endometrial gene expression

There were 2509 genes with significantly altered expression levels (adjusted p ≤ 0.05) in LNG-IUS users compared to control samples a; the top 50 genes are shown in Table 2 and the complete list of differentially expressed genes is provided in Supp Table 1.

**Table 2:** Top 50 differentially expressed genes in endometrial biopsies from LNG-IUS users compared to controls presented hierarchically by adjusted P value.

Symbol	GENENAME	Log2 Fold Change	Adjusted P Value
C6orf141	chromosome 6 open reading frame 141	−2.093	6.55E-09
MT1G	metallothionein 1G	−3.286	6.08E-08
MT1E	metallothionein 1E	−2.150	1.55E-07
MT1F	metallothionein 1F	−2.978	1.55E-07
PHYHIPL	phytanoyl-CoA 2-hydroxylase interacting protein like	−3.968	4.02E-07
FAM84B	family with sequence similarity 84 member B	−2.039	7.02E-07
CCL2	C-C motif chemokine ligand 2	3.339	7.02E-07
C9orf152	chromosome 9 open reading frame 152	−2.317	7.22E-07
CRISP3	cysteine rich secretory protein 3	−3.961	1.21E-06
CWH43	cell wall biogenesis 43 C-terminal homolog	−2.371	2.19E-06
MT1M	metallothionein 1M	−3.446	2.40E-06
MT1H	metallothionein 1H	−3.299	2.40E-06
IFIT1	interferon induced protein with tetratricopeptide repeats 1	2.017	2.40E-06
GCNT3	glucosaminyl (N-acetyl) transferase 3, mucin type	−1.753	2.40E-06
UPK1B	uroplakin 1B	−3.443	4.63E-06
SLC15A1	solute carrier family 15 member 1	−3.211	4.63E-06
SLC5A1	solute carrier family 5 member 1	−1.944	4.63E-06
IFI44L	interferon induced protein 44 like	3.015	4.63E-06
MT1L	metallothionein 1L (gene/pseudogene)	−2.688	4.63E-06
HSD17B2	hydroxysteroid 17-beta dehydrogenase 2	−1.812	5.18E-06
SLC30A2	solute carrier family 30 member 2	−1.627	6.43E-06
PLA2G16	phospholipase A2 group XVI	−1.598	6.43E-06
DNAJC15	DnaJ heat shock protein family (Hsp40) member C15	−1.173	1.00E-05
MRC1	mannose receptor, C type 1	3.027	1.24E-05
MRC1	mannose receptor, C type 1	3.027	1.24E-05
HGD	homogentisate 1,2-dioxygenase	−3.030	1.33E-05
HGD	homogentisate 1,2-dioxygenase	−3.030	1.33E-05
ELMO1	engulfment and cell motility 1	1.730	1.42E-05
IL20RA	interleukin 20 receptor subunit alpha	−2.157	1.54E-05
GJB1	gap junction protein beta 1	−1.172	1.57E-05
FAM177A1	family with sequence similarity 177 member A1	−1.834	1.87E-05
DTNA	dystrobrevin alpha	1.448	1.87E-05
MANSC1	MANSC domain containing 1	−1.469	2.01E-05
TNS1	tensin 1	1.504	2.01E-05
CLDN4	claudin 4	−1.411	2.01E-05
PLXDC2	plexin domain containing 2	1.325	2.05E-05
PLCB4	phospholipase C beta 4	−1.932	2.19E-05
TREM1	triggering receptor expressed on myeloid cells 1	3.154	2.19E-05
IGFBP1	insulin like growth factor binding protein 1	2.652	2.19E-05
IL10RA	interleukin 10 receptor subunit alpha	2.022	2.53E-05
SRD5A3	steroid 5 alpha-reductase 3	−1.406	2.58E-05
FCGR2B	Fc fragment of IgG receptor IIb	3.266	2.58E-05
MFSD4A	major facilitator superfamily domain containing 4A	−1.855	2.58E-05
TPD52L1	tumor protein D52-like 1	−2.255	2.58E-05
EPYC	epiphycan	4.785	2.74E-05
MT1X	metallothionein 1×	−2.069	2.86E-05
TCN1	transcobalamin 1	−3.623	3.10E-05
WIPF1	WAS/WASL interacting protein family member 1	1.700	3.20E-05
MX1	MX dynamin like GTPase 1	1.798	3.29E-05
CTSL	cathepsin L	2.027	3.29E-05

The Reactome pathway categories altered in LNG-IUS users are shown in Table 3 and indicate that differentially expressed genes mapped predominantly onto immune and inflammatory pathways. A complete list of pathways that showed altered expression with a combined score (defined in section 2.5 above)>5 is shown in Supp Table 2.

**Table 3:** Pathway categories showing altered expression from Reactome 2016 presented hierarchically by combined score values.

Levonorgestrel-releasing intrauterine system versus control	P value	Z score	Combined score
Immune system	2.91E-34	−2.23	172.37
Hemostasis	1.27E-18	−2.14	88.2
Cytokine signaling in Immune system	2.15E-16	−2.39	86.11
Innate immune system	1.51E-18	−2.4	82.05
Platelet activation, signaling and aggregation	9.94E-14	−2.12	63.48
Interferon signaling	7.82E-14	−2.07	62.46
Adaptive immune system	8.64E-12	−2.22	56.43
Interferon gamma signaling	1.40E-13	−1.74	51.35
Immunoregulatory interactions between a lymphoid and a non-lymphoid cell	4.04E-12	−1.95	51.26
Generation of second messenger molecules	1.23E-10	−1.89	43.22
**Combined oral contraceptives versus control**			
Metallothioneins bind metals	7.37E-19	−1.85	77.15
Response to metal ions	7.37E-19	−1.8	75.23
Metabolism	8.92E-07	−2.23	31.05
Acyl chain remodeling of phosphatidylcholine	5.31E-04	−1.88	14.19
Acyl chain remodeling of phosphatidylinositol	4.60E-03	−1.86	10.03
O-linked glycosylation of mucins	8.74E-03	−2.02	9.58
Acyl chain remodeling of phosphatidylserine	5.19E-03	−1.73	9.1
Synthesis and interconversion of nucleotide di-and triphosphates	1.11E-02	−1.94	8.75
Diseases associated with glycosaminoglycan metabolism	1.20E-02	−1.9	8.41
Acyl chain remodeling of phosphatidylethanolamine	9.42E-03	−1.79	8.36

Given that the samples in the LNG-IUS groups included a combination of those from women who had ovulated and those who had not, we performed a sensitivity analysis to determine the effect of ovulatory status on our findings, by comparing gene expression in LNG-IUS users who had ovulated (n = 4) to controls, all of whom had ovulated. We found 1650 genes being associated with LNG-IUS use versus those in the control group (adjusted p-value <0.05). The decrease from the original 2509 genes is expected given the lower statistical power to declare significance resulting from the use of a smaller number of samples (n = 4 versus n = 11). There is an overlap of 1390 genes between these two sets of genes, or 84% of genes passing statistical threshold in the reduced number of samples were also in the original list of associated genes. The concordance is also apparent when one associates the estimated fold-changes of the expression of 2509 genes identified using all the data versus the estimated fold-changes of expression of these genes using data for only those LNG-IUS samples who had ovulated (Supplemental Figure). These results indicate that lack of ovulation was not the primary driver of our results in the LNG-IUS group.

### Effect of COC on Endometrial Gene Expression

There were 133 genes with significantly altered expression (adjusted p ≤ 0.05) in COC users compared to control samples; the top 50 genes are shown in Table 4 and the complete gene list is provided in Supp Table 3. The Reactome pathway categories that are altered in COC users are shown in Table 3 and indicate that the differentially expressed genes mapped predominantly onto pathways involving metal ions and acyl chain remodeling. A complete list of pathways that showed altered expression with a combined score >5 is shown in Supp Table 4.

**Table 4:** Top 50 differentially expressed genes in endometrial biopsies from COC users compared to controls presented hierarchically by adjusted P value.

SYMBOL	GENENAME	Log2 Fold Change	Adjusted P Value
MT2A	metallothionein 2A	−2.404	7.33E-05
MT1M	metallothionein 1M	−3.467	7.33E-05
MT2A	metallothionein 2A	−2.198	9.04E-05
MT1JP	metallothionein 1J, pseudogene	−2.030	0.0002
MT1A	metallothionein 1A	−2.232	0.0002
S100P	S100 calcium binding protein P	−3.685	0.0002
MT1F	metallothionein 1F	−2.747	0.0003
MT1X	metallothionein 1×	−2.371	0.0003
DHCR24	24-dehydrocholesterol reductase	−1.716	0.0003
MT1L	metallothionein 1L (gene/pseudogene)	−3.102	0.0003
MT1HL1	metallothionein 1H-like 1	−1.798	0.0003
ATRNL1	attractin like 1	1.713	0.0003
MT1G	metallothionein 1G	−3.451	0.0003
MT1B	metallothionein 1B	−1.394	0.0003
SLC30A2	solute carrier family 30 member 2	−1.450	0.0008
MT2A	metallothionein 2A	−1.604	0.0010
MFSD4A	major facilitator superfamily domain containing 4A	−1.938	0.0016
MT1H	metallothionein 1H	−3.206	0.0023
MT1E	metallothionein 1E	−2.243	0.0024
AIMP1	aminoacyl tRNA synthetase complex interacting multifunctional protein 1	−1.801	0.0024
FABP5	fatty acid binding protein 5	−1.497	0.0024
FABP5	fatty acid binding protein 5	−1.521	0.0024
SLC5A1	solute carrier family 5 member 1	−1.797	0.0025
PLXNC1	plexin C1	1.353	0.0025
SFN	stratifin	−0.995	0.0026
CATSPERB	cation channel sperm associated auxiliary subunit beta	−2.608	0.0029
AVPR1A	arginine vasopressin receptor 1A	1.407	0.0031
ANXA2	annexin A2	−1.079	0.0031
TMEM154	transmembrane protein 154	−2.000	0.0036
STEAP1	STEAP family member 1	−1.471	0.0039
BNC2	basonuclin 2	1.362	0.0046
PLA2G2A	phospholipase A2 group IIA	−2.489	0.0047
DEPDC1B	DEP domain containing 1B	−1.692	0.0055
LURAP1L	leucine rich adaptor protein 1 like	−1.026	0.0063
ESR1	estrogen receptor 1	1.401	0.0065
OTUB2	OTU deubiquitinase, ubiquitin aldehyde binding 2	−0.776	0.0077
TRIM5	tripartite motif containing 5	0.731	0.0088
TC2N	tandem C2 domains, nuclear	−1.493	0.0088
L3MBTL3	l(3)mbt-like 3 (Drosophila)	0.828	0.0111
GPX1	glutathione peroxidase 1	−1.019	0.0127
ZNF750	zinc finger protein 750	−1.564	0.0128
CAPZA2	capping actin protein of muscle Z-line alpha subunit 2	−1.309	0.0129
MMP7	matrix metallopeptidase 7	3.066	0.0134
DPP6	dipeptidyl peptidase like 6	1.668	0.0141
LPCAT2	lysophosphatidylcholine acyltransferase 2	1.134	0.0141
CPED1	cadherin like and PC-esterase domain containing 1	1.230	0.0141
PRELP	proline and arginine rich end leucine rich repeat protein	0.801	0.0141
ACACB	acetyl-CoA carboxylase beta	0.558	0.0147
FXYD3	FXYD domain containing ion transport regulator 3	−1.294	0.0147
RHPN2	rhophilin Rho GTPase binding protein 2	−1.396	0.0147

## Discussion

Our results demonstrate that of the three contraceptive methods examined in this study, the LNG-IUS had the strongest effect on the endometrial transcriptome, resulting in significant alterations in genes regulating immune and inflammatory pathways. These results validate findings previously published by our group in an independent set of samples from LNG-IUS users^7^. We performed the present study in part to test the hypothesis that different types of IUDs, as foreign bodies in the uterus, would elicit common changes in the endometrium. Instead, we found that the LNG-IUS resulted in differential expression of 2509 genes, whereas the cu-IUD group showed no effect. This unexpected finding suggests that the predominant driver of the inflammatory signal we observed with LNG-IUS is due to the locally released LNG, and is not a result of a foreign body per se. Previous work has demonstrated that LNG is implicated in oxidative stress and apoptosis, which could contribute to the effects we observed on the endometrial transcriptome^21,22^. To avoid measuring perturbations resulting from the insertion itself, participants were required to have had the IUD for ≥6 months, thus we do not think the transcriptional differences are related to length of use of the device. Previous morphological analyses demonstrated that cu-IUDs cause a foreign body reaction and leukocyte infiltration in the endometrium^23,24^; the fact that we did not see this effect in the global endometrial transcriptome could be due to our analysis sampling a larger surface area of the uterine cavity and containing both endometrium and stroma, which might have blunted the ability to detect the local effect seen previously on immunohistochemistry.

A prior analysis of endometrial RNA expression in users of an inert IUD demonstrated 147 genes that were significantly dysregulated in the first month after IUD insertion^25^, whereas our study showed no significantly differentially expressed genes in cu-IUD users with a median use of 23 months. Their study looked at Lippes loop IUD, not cu-IUD; the length of IUD exposure was longer in our study, and hence the effects they observed might be attenuated over time; and the statistical methods used to analyze gene expression were different. In our study, samples from both the control and the cu-IUD groups were obtained in the mid-luteal phase as confirmed by timing the sample collection 7–11 days after the LH surge and by serum progesterone levels >2 ng/ml at the time of sample collection. Our results suggest that the effects of the cu-IUD on the endometrial transcriptome are subtle and might be dominated by the progesterone effects of the luteal phase.

The effect of COCs on the endometrial transcriptome was intermediate between the cu-IUD and the LNG-IUS. Unlike the LNG-IUS, COCs did not cause changes in inflammatory pathways, however both the LNG-IUS and COCs caused significant downregulation of several members of the metallothionein (MT) gene family. MT has well-documented roles in binding of heavy metals including copper for homeostasis and detoxification, and is increasingly being recognized for its role in immunomodulation, apoptosis and the stress response^26,27^. Exposure to LNG-IUS in women with endometrial hyperplasia resulted in decreased immunohistochemical detection of MT protein^28^, consistent with our results showing down-regulation of MT RNA in endometrium in both LNG-exposed groups. Release of copper ions from the cu-IUD is postulated to be an important factor for its contraceptive efficacy through spermicidal effects but we did not observe a change in MT gene expression in the cu-IUD group. The effect on MT gene expression in the LNG-IUS and COC groups, and not in the cu-IUD group, supports a common effect of LNG on the endometrium in users of LNG-containing contraceptives related to the stress response functions of MT genes.

In our previous work, we reported 23 genes with altered expression in cervical biopsies from controls compared to LNG-IUS users^7^, whereas in this study we found no significant differences in the cervical transcriptomes. The low number of differentially expressed genes and low fold-change in expression levels (maximum of 2.4-fold) in the prior study indicated a relatively weak effect of LNG-IUS on the cervix^7^. We attribute our inability to replicate this finding in the current study to the differences in the statistical methods used including methods for normalization, for comparisons of gene expression, and adjustment for false discovery rate.

This work has several strengths. The parallel processing of samples from 4 groups of women provided a direct comparison of contraceptive transcriptomes that to our knowledge has not previously been performed. In cycling women, we synchronized collection to the mid-luteal phase to limit the effects of cyclical hormonal variation on our assays, and to compare contraceptive effects on the endometrial environment at the time of implantation. The rigorous methodology for statistical comparison of gene expression resulted in the detection of robust differences between the groups. Our study also has limitations. The control group was older than the other groups, resulting in adjustment for age in the analysis. Our sample sizes were relatively small, which may have limited our ability to demonstrate more subtle differences between some of the groups. We were unable to time sample collection precisely in the menstrual cycle in women on LNG-IUS because the majority of cycles were anovulatory, although our sensitivity analysis indicated that ovulatory status did not affect the results. The failure to find differences between the cervical transcriptomes could be due to the timing of sample collection in the luteal phase, when the hormonal and/or contraceptive effects would be predicted to be have produced unfavorable mucus in all groups. Finally, the cu-IUD is not a perfect comparator to the LNG-IUS: the ideal comparison would be the same IUD with and without LNG, and an ideal comparator to the LNG-IUS group would be locally applied rather than systemic LNG. Nevertheless, our findings, particularly with respect to differences in IUD groups, indicate a strong effect of local LNG on gene expression in the endometrium.

In summary, our results demonstrate that the LNG-IUS and cu-IUD differ significantly in their effects on the endometrial transcriptome. Whereas the LNG-IUS induces many changes in immune and inflammatory genes and pathways, endometrium from cu-IUD users is indistinguishable from the luteal phase endometrium in the control group. These results suggest the presence of a foreign body per se has less effect on the endometrium than locally released LNG and argue against a foreign body reaction as a common mechanism of action of IUDs.

## Supplementary information

Supplementary information.

Supplementary information2.
