# Supplementary material for: Differential Effects of the Hormonal and Copper Intrauterine Device on the Endometrial Transcriptome
Source: Sci Rep. 2020 Apr 23;10:6888. doi: 10.1038/s41598-020-63798-8 (PMC7181869; doi:10.1038/s41598-020-63798-8)
Supplement: Supplementary file 1 — Supplementary information. [file 41598_2020_63798_MOESM1_ESM.pdf]

Supplemental figure for:

Differential Effects of the Hormonal and Copper Intrauterine Device on the Endometrial Transcriptome

Karen Smith-McCune[1]*, Reuben Thomas[2], Sarah Averbach[1,3], Dominika Seidman[1], Margaret Takeda[1], Sahar Houshdaran[1], Linda C Giudice[1]

Author affiliations:

[1] Department of Obstetrics, Gynecology and Reproductive Sciences, University of California San Francisco, San Francisco, CA

[2] Gladstone Institutes, San Francisco, CA

[3] Current address: Department of Obstetrics, Gynecology and Reproductive Sciences, University of California San Diego, CA

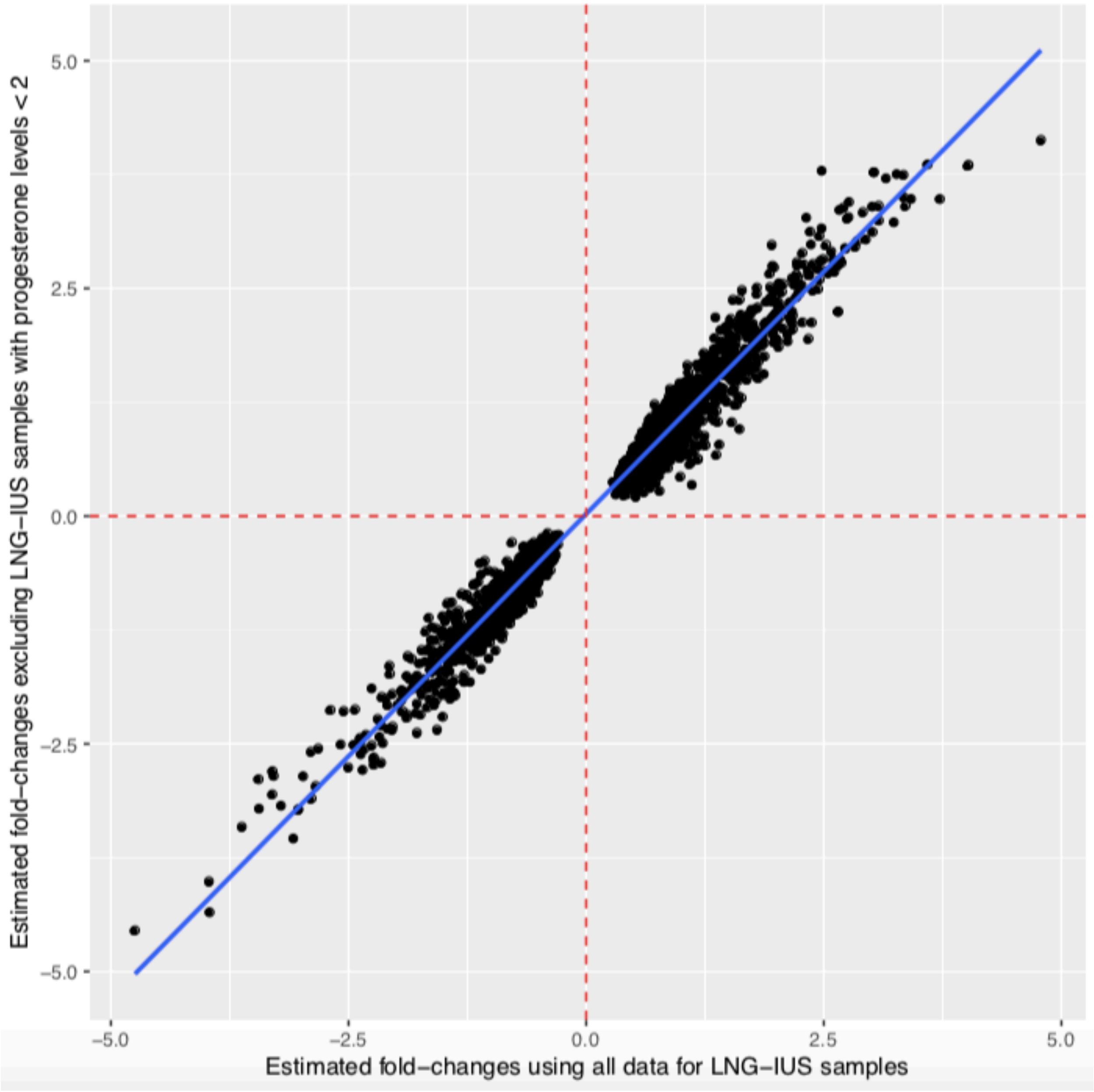

5.0
2.5
0.0
-2.5
-5.0
-5.0
-2.5
0.0
2.5
5.0
Estimated fold−changes using all data for LNG−IUS samples
Estimated fold−changes excluding LNG−IUS samples with progesterone levels < 2

Supplemental Figure Legend: Concordance of results of gene expression in LNG-IUS samples overall with results from ovulatory samples.

Scatter plot with best fit line demonstrates the estimated fold-changes of the expression of 2509 genes identified using all the data (x-axis) plotted against the estimated fold-changes of expression of these genes using data for only those LNG-IUS samples who had ovulated (progesterone >2) (y-axis).
