# Supplementary material for: Differential Effects of the Hormonal and Copper Intrauterine Device on the Endometrial Transcriptome
Source: Sci Rep. 2020 Apr 23;10:6888. doi: 10.1038/s41598-020-63798-8 (PMC7181869; doi:10.1038/s41598-020-63798-8)
Supplement: Supplementary file 2 — Supplementary information2. [file 41598_2020_63798_MOESM2_ESM.pdf]

Supplemental material for "**Differential Effects of the Hormonal and Copper Intrauterine Device on the Endometrial Transcriptome**"

Karen Smith-McCune[1], Reuben Thomas[2], Sarah Averbach[1,3], Dominika Seidman[1], Margaret Takeda[1], Sahar Houshdaran[1], Linda C Giudice[1]

Author affiliations:

[1] Department of Obstetrics, Gynecology and Reproductive Sciences, University of California San Francisco, San Francisco, CA

[2] Gladstone Institutes, San Francisco, CA

[3] Current address: Department of Obstetrics, Gynecology and Reproductive Sciences, University of California San Diego, CA

Supplemental Table 1. Differentially expressed genes LNG-IUS users versus controls

SYMBOL	GENENAME	log2FC	adj.P.Val
C6orf141	chromosome 6 open reading frame 141	-2.093	6.55E-09
MT1G	metallothionein 1G	-3.286	6.08E-08
MT1E	metallothionein 1E	-2.150	1.55E-07
MT1F	metallothionein 1F	-2.978	1.55E-07
PHYHIPL	phytanoyl-CoA 2-hydroxylase interacting protein like	-3.968	4.02E-07
FAM84B	family with sequence similarity 84 member B	-2.039	7.02E-07
CCL2	C-C motif chemokine ligand 2	3.339	7.02E-07
C9orf152	chromosome 9 open reading frame 152	-2.317	7.22E-07
CRISP3	cysteine rich secretory protein 3	-3.961	1.21E-06
CWH43	cell wall biogenesis 43 C-terminal homolog	-2.371	2.19E-06
MT1M	metallothionein 1M	-3.446	2.40E-06
MT1H	metallothionein 1H	-3.299	2.40E-06
IFIT1	interferon induced protein with tetratricopeptide repeats 1	2.017	2.40E-06
GCNT3	glucosaminyl (N-acetyl) transferase 3, mucin type	-1.753	2.40E-06
UPK1B	uroplakin 1B	-3.443	4.63E-06
SLC15A1	solute carrier family 15 member 1	-3.211	4.63E-06
SLC5A1	solute carrier family 5 member 1	-1.944	4.63E-06
IFI44L	interferon induced protein 44 like	3.015	4.63E-06
MT1L	metallothionein 1L (gene/pseudogene)	-2.688	4.63E-06
HSD17B2	hydroxysteroid 17-beta dehydrogenase 2	-1.812	5.18E-06
SLC30A2	solute carrier family 30 member 2	-1.627	6.43E-06
PLA2G16	phospholipase A2 group XVI	-1.598	6.43E-06
DNAJC15	DnaJ heat shock protein family (Hsp40) member C15	-1.173	1.00E-05
MRC1	mannose receptor, C type 1	3.027	1.24E-05
MRC1	mannose receptor, C type 1	3.027	1.24E-05
HGD	homogentisate 1,2-dioxygenase	-3.030	1.33E-05
HGD	homogentisate 1,2-dioxygenase	-3.030	1.33E-05
ELMO1	engulfment and cell motility 1	1.730	1.42E-05
IL20RA	interleukin 20 receptor subunit alpha	-2.157	1.54E-05
GJB1	gap junction protein beta 1	-1.172	1.57E-05
FAM177A1	family with sequence similarity 177 member A1	-1.834	1.87E-05
DTNA	dystrobrevin alpha	1.448	1.87E-05
MANSC1	MANSC domain containing 1	-1.469	2.01E-05
TNS1	tensin 1	1.504	2.01E-05
CLDN4	claudin 4	-1.411	2.01E-05
PLXDC2	plexin domain containing 2	1.325	2.05E-05
PLCB4	phospholipase C beta 4	-1.932	2.19E-05
TREM1	triggering receptor expressed on myeloid cells 1	3.154	2.19E-05
IGFBP1	insulin like growth factor binding protein 1	2.652	2.19E-05
IL10RA	interleukin 10 receptor subunit alpha	2.022	2.53E-05
SRD5A3	steroid 5 alpha-reductase 3	-1.406	2.58E-05
FCGR2B	Fc fragment of IgG receptor IIb	3.266	2.58E-05
MFSD4A	major facilitator superfamily domain containing 4A	-1.855	2.58E-05
TPD52L1	tumor protein D52-like 1	-2.255	2.58E-05

EPYC	epiphycan	4.785	2.74E-05
MT1X	metallothionein 1X	-2.069	2.86E-05
TCN1	transcobalamin 1	-3.623	3.10E-05
WIPF1	WAS/WASL interacting protein family member 1	1.700	3.20E-05
MX1	MX dynamin like GTPase 1	1.798	3.29E-05
CTSL	cathepsin L	2.027	3.29E-05
C11orf88	chromosome 11 open reading frame 88	-1.677	3.51E-05
CCL22	C-C motif chemokine ligand 22	2.244	3.79E-05
FAM149A	family with sequence similarity 149 member A	-1.890	3.79E-05
FXYD3	FXYD domain containing ion transport regulator 3	-1.598	3.79E-05
ELMO1	engulfment and cell motility 1	1.813	4.13E-05
MYO5A	myosin VA	1.177	4.13E-05
TSPAN8	tetraspanin 8	-3.302	4.13E-05
PLD1	phospholipase D1	1.365	4.13E-05
RHPN2	rhophilin Rho GTPase binding protein 2	-1.316	4.13E-05
LPCAT2	lysophosphatidylcholine acyltransferase 2	1.545	4.13E-05
GNPNAT1	glucosamine-phosphate N-acetyltransferase 1	-0.883	4.13E-05
CNR1	cannabinoid receptor 1	2.831	4.13E-05
PLXNC1	plexin C1	1.254	4.13E-05
TMEM45B	transmembrane protein 45B	-1.511	4.25E-05
MT1A	metallothionein 1A	-1.756	4.40E-05
FPR3	formyl peptide receptor 3	2.706	4.42E-05
EFNA1	ephrin A1	-1.288	4.44E-05
PTPRE	protein tyrosine phosphatase, receptor type E	1.468	4.49E-05
SST	somatostatin	3.344	5.32E-05
PTPRD	protein tyrosine phosphatase, receptor type D	-1.065	5.62E-05
ARHGAP31	Rho GTPase activating protein 31	0.971	5.66E-05
MS4A14	membrane spanning 4-domains A14	2.164	5.66E-05
CD300A	CD300a molecule	1.277	5.78E-05
DOCK11	dedicator of cytokinesis 11	1.745	5.78E-05
SAMD9	sterile alpha motif domain containing 9	1.475	6.02E-05
MICAL2	microtubule associated monooxygenase, calponin and LIM dom	1.581	6.02E-05
ADGRE5	adhesion G protein-coupled receptor E5	1.849	6.31E-05
NQO1	NAD(P)H quinone dehydrogenase 1	-1.502	6.32E-05
RIMKLB	ribosomal modification protein rimK like family member B	-2.378	6.35E-05
DDX52	DEAD-box helicase 52	-2.108	6.58E-05
TRIM22	tripartite motif containing 22	1.287	6.58E-05
AMIGO2	adhesion molecule with Ig like domain 2	-1.299	6.58E-05
AOAH	acyloxyacyl hydrolase	1.999	6.60E-05
ST6GALNAC1	ST6 N-acetylgalactosaminide alpha-2,6-sialyltransferase 1	-1.447	6.60E-05
SCGB1D4	secretoglobin family 1D member 4	-4.747	6.60E-05
FAM3C	family with sequence similarity 3 member C	-1.177	6.80E-05
ST6GALNAC1	ST6 N-acetylgalactosaminide alpha-2,6-sialyltransferase 1	-1.412	6.87E-05
ZNF816-ZNF321I	ZNF816-ZNF321P readthrough	-1.388	7.19E-05
CYBB	cytochrome b-245 beta chain	2.637	7.19E-05

IFI30	IFI30, lysosomal thiol reductase	2.525	7.28E-05
WDFY4	WDFY family member 4	1.161	7.35E-05
NRP2	neuropilin 2	2.061	7.35E-05
TC2N	tandem C2 domains, nuclear	-1.742	7.35E-05
ACSL5	acyl-CoA synthetase long-chain family member 5	-1.572	7.35E-05
NCKAP1L	NCK associated protein 1 like	2.206	7.35E-05
SPTLC3	serine palmitoyltransferase long chain base subunit 3	-2.040	7.97E-05
DHCR24	24-dehydrocholesterol reductase	-1.252	7.97E-05
ALOX5AP	arachidonate 5-lipoxygenase activating protein	2.728	7.97E-05
IL7R	interleukin 7 receptor	3.594	7.97E-05
KIAA1324	KIAA1324	-2.844	8.12E-05
ASAP1	ArfGAP with SH3 domain, ankyrin repeat and PH domain 1	1.223	8.12E-05
RNASE1	ribonuclease A family member 1, pancreatic	2.223	8.12E-05
MX2	MX dynamin like GTPase 2	1.997	8.13E-05
AIMP1	aminoacyl tRNA synthetase complex interacting multifunctional p	-1.803	8.14E-05
GRAMD1C	GRAM domain containing 1C	-2.331	8.25E-05
WEE1	WEE1 G2 checkpoint kinase	-1.038	8.25E-05
CD86	CD86 molecule	2.455	8.32E-05
FAM3C	family with sequence similarity 3 member C	-1.163	8.85E-05
MT2A	metallothionein 2A	-1.687	8.94E-05
PAPSS1	3'-phosphoadenosine 5'-phosphosulfate synthase 1	-1.845	9.48E-05
HLA-DRA	major histocompatibility complex, class II, DR alpha	2.612	9.55E-05
IL6ST	interleukin 6 signal transducer	-1.506	9.67E-05
MMP7	matrix metallopeptidase 7	3.722	0.0001
DYNLT3	dynein light chain Tctex-type 3	-1.634	0.0001
MT2A	metallothionein 2A	-1.509	0.0001
CD24	CD24 molecule	-1.520	0.0001
GAST	gastrin	-2.895	0.0001
F13A1	coagulation factor XIII A chain	1.895	0.0001
OAS1	2'-5'-oligoadenylate synthetase 1	1.508	0.0001
HLA-DRA	major histocompatibility complex, class II, DR alpha	2.546	0.0001
ITGAM	integrin subunit alpha M	2.071	0.0001
HLA-DRA	major histocompatibility complex, class II, DR alpha	2.564	0.0001
EPB41L4B	erythrocyte membrane protein band 4.1 like 4B	-0.885	0.0001
VSIG4	V-set and immunoglobulin domain containing 4	2.292	0.0001
MT1JP	metallothionein 1J, pseudogene	-1.486	0.0001
ARG2	arginase 2	-1.411	0.0001
HSDL2	hydroxysteroid dehydrogenase like 2	-0.845	0.0001
B4GALNT3	beta-1,4-N-acetyl-galactosaminyltransferase 3	-1.617	0.0001
ADGRE2	adhesion G protein-coupled receptor E2	1.751	0.0001
PSTK	phosphoseryl-tRNA kinase	-1.147	0.0001
ENTPD1	ectonucleoside triphosphate diphosphohydrolase 1	1.086	0.0001
CYTIP	cytohesin 1 interacting protein	3.078	0.0001
ADAM19	ADAM metallopeptidase domain 19	0.937	0.0001
C3AR1	complement C3a receptor 1	2.476	0.0001

ID1	inhibitor of DNA binding 1, HLH protein	-1.224	0.0001
KCNMB1	potassium calcium-activated channel subfamily M regulatory beta	0.747	0.0001
RGCC	regulator of cell cycle	1.574	0.0001
RSAD2	radical S-adenosyl methionine domain containing 2	1.759	0.0001
HN1L	hematological and neurological expressed 1 like	-1.035	0.0001
SYT11	synaptotagmin 11	1.005	0.0001
TMEM30B	transmembrane protein 30B	-0.972	0.0001
LAPTM5	lysosomal protein transmembrane 5	2.495	0.0001
GMNN	geminin, DNA replication inhibitor	-1.799	0.0001
PLCB2	phospholipase C beta 2	1.311	0.0001
GPR137B	G protein-coupled receptor 137B	1.266	0.0001
SFRP4	secreted frizzled related protein 4	-2.819	0.0001
ITGAX	integrin subunit alpha X	1.928	0.0001
GNG2	G protein subunit gamma 2	1.349	0.0001
NRP2	neuropilin 2	1.487	0.0001
LPAR3	lysophosphatidic acid receptor 3	-1.740	0.0002
JAKMIP2	janus kinase and microtubule interacting protein 2	1.270	0.0002
FMNL3	formin like 3	0.817	0.0002
SEPT6	septin 6	1.305	0.0002
ALDH6A1	aldehyde dehydrogenase 6 family member A1	-1.291	0.0002
SAMHD1	SAM and HD domain containing deoxynucleoside triphosphate tr	1.159	0.0002
CATSPERB	cation channel sperm associated auxiliary subunit beta	-2.582	0.0002
IDS	iduronate 2-sulfatase	0.726	0.0002
LILRB2	leukocyte immunoglobulin like receptor B2	1.303	0.0002
CDYL2	chromodomain Y-like 2	-1.034	0.0002
PERP	PERP, TP53 apoptosis effector	-1.302	0.0002
DEPDC1B	DEP domain containing 1B	-1.639	0.0002
HCLS1	hematopoietic cell-specific Lyn substrate 1	1.893	0.0002
DDX60L	DEAD-box helicase 60-like	1.203	0.0002
CCL23	C-C motif chemokine ligand 23	1.226	0.0002
BCL2L11	BCL2 like 11	0.773	0.0002
SLC5A3	solute carrier family 5 member 3	1.252	0.0002
PARP12	poly(ADP-ribose) polymerase family member 12	0.731	0.0002
LPL	lipoprotein lipase	1.977	0.0002
OAS2	2'-5'-oligoadenylate synthetase 2	1.510	0.0002
CD74	CD74 molecule	2.441	0.0002
FXYD5	FXYD domain containing ion transport regulator 5	1.203	0.0002
CD53	CD53 molecule	2.386	0.0002
TLR3	toll like receptor 3	-1.681	0.0002
ITGA6	integrin subunit alpha 6	-1.022	0.0002
NOSTRIN	nitric oxide synthase trafficking	-2.447	0.0002
ZNF253	zinc finger protein 253	-0.951	0.0002
SCIMP	SLP adaptor and CSK interacting membrane protein	2.228	0.0002
DEFB1	defensin beta 1	-1.943	0.0002
THEMIS2	thymocyte selection associated family member 2	1.275	0.0002

ARRB2	arrestin beta 2	1.802	0.0002
CD14	CD14 molecule	2.211	0.0002
PTK2B	protein tyrosine kinase 2 beta	1.008	0.0002
MLKL	mixed lineage kinase domain like	1.100	0.0002
RGS2	regulator of G-protein signaling 2	2.169	0.0002
IFI44	interferon induced protein 44	1.858	0.0002
EFEMP1	EGF containing fibulin like extracellular matrix protein 1	1.816	0.0002
FAM129A	family with sequence similarity 129 member A	1.729	0.0002
TGFBR1	transforming growth factor beta receptor 1	1.059	0.0002
SCGB2A1	secretoglobin family 2A member 1	-3.082	0.0002
HOOK1	hook microtubule tethering protein 1	-1.781	0.0002
IGSF6	immunoglobulin superfamily member 6	2.267	0.0002
CDK14	cyclin dependent kinase 14	0.931	0.0002
SNX7	sorting nexin 7	-1.273	0.0002
IL1RL1	interleukin 1 receptor like 1	2.578	0.0002
ALOX5	arachidonate 5-lipoxygenase	1.917	0.0002
SLC5A3	solute carrier family 5 member 3	1.559	0.0002
DOCK2	dedicator of cytokinesis 2	1.832	0.0002
ADGRL3	adhesion G protein-coupled receptor L3	-1.698	0.0002
IGFBP3	insulin like growth factor binding protein 3	1.877	0.0002
GPNMB	glycoprotein nmb	2.464	0.0002
MTSS1	MTSS1, I-BAR domain containing	1.203	0.0002
NCF4	neutrophil cytosolic factor 4	1.786	0.0002
HLA-DPA1	major histocompatibility complex, class II, DP alpha 1	1.988	0.0002
HLA-DPA1	major histocompatibility complex, class II, DP alpha 1	1.988	0.0002
LARP1B	La ribonucleoprotein domain family member 1B	-1.206	0.0002
PARP15	poly(ADP-ribose) polymerase family member 15	1.495	0.0002
SLAMF7	SLAM family member 7	1.415	0.0002
PHLPP1	PH domain and leucine rich repeat protein phosphatase 1	-0.904	0.0002
CXCL14	C-X-C motif chemokine ligand 14	-2.257	0.0002
TPP1	tripeptidyl peptidase 1	1.349	0.0002
MT1B	metallothionein 1B	-1.129	0.0002
ST3GAL5	ST3 beta-galactoside alpha-2,3-sialyltransferase 5	1.636	0.0002
ATP6V1C2	ATPase H+ transporting V1 subunit C2	-2.175	0.0002
HLA-DPA1	major histocompatibility complex, class II, DP alpha 1	2.175	0.0002
GIMAP4	GTPase, IMAP family member 4	1.716	0.0003
MPEG1	macrophage expressed 1	1.771	0.0003
SLAMF8	SLAM family member 8	1.948	0.0003
PARP8	poly(ADP-ribose) polymerase family member 8	1.160	0.0003
PLBD1	phospholipase B domain containing 1	1.487	0.0003
LCP2	lymphocyte cytosolic protein 2	1.947	0.0003
GCSH	glycine cleavage system protein H	-1.480	0.0003
FAM13C	family with sequence similarity 13 member C	-1.021	0.0003
ID4	inhibitor of DNA binding 4, HLH protein	-1.441	0.0003
CLEC2D	C-type lectin domain family 2 member D	1.135	0.0003

BAIAP2L1	BAI1 associated protein 2 like 1	-1.400	0.0003
CSF1R	colony stimulating factor 1 receptor	1.725	0.0003
TRPV2	transient receptor potential cation channel subfamily V member 2	1.098	0.0003
XBP1	X-box binding protein 1	-0.725	0.0003
SP100	SP100 nuclear antigen	0.841	0.0003
CD163	CD163 molecule	2.744	0.0003
SAMD9L	sterile alpha motif domain containing 9 like	1.351	0.0003
MSR1	macrophage scavenger receptor 1	2.365	0.0003
SLC18A2	solute carrier family 18 member A2	-2.063	0.0003
STAT1	signal transducer and activator of transcription 1	1.169	0.0003
ANKRD20A2	ankyrin repeat domain 20 family member A2	-1.428	0.0003
MT1HL1	metallothionein 1H-like 1	-1.309	0.0003
ZNF750	zinc finger protein 750	-1.603	0.0003
ID3	inhibitor of DNA binding 3, HLH protein	-0.962	0.0003
FBP1	fructose-bisphosphatase 1	3.080	0.0003
SP140L	SP140 nuclear body protein like	0.912	0.0003
PRELP	proline and arginine rich end leucine rich repeat protein	1.182	0.0003
GCSH	glycine cleavage system protein H	-1.466	0.0003
RAB15	RAB15, member RAS oncogene family	-0.860	0.0003
MMP12	matrix metallopeptidase 12	3.237	0.0003
SP140	SP140 nuclear body protein	1.360	0.0003
IL1R2	interleukin 1 receptor type 2	1.935	0.0003
IRF4	interferon regulatory factor 4	1.002	0.0003
DOCK8	dedicator of cytokinesis 8	1.968	0.0003
ANKRD20A2	ankyrin repeat domain 20 family member A2	-1.165	0.0003
ANKRD20A2	ankyrin repeat domain 20 family member A2	-1.165	0.0003
MRPL21	mitochondrial ribosomal protein L21	-0.885	0.0004
MANBA	mannosidase beta	1.150	0.0004
IFI6	interferon alpha inducible protein 6	0.935	0.0004
GCAT	glycine C-acetyltransferase	-0.777	0.0004
CREB5	cAMP responsive element binding protein 5	1.262	0.0004
DOCK10	dedicator of cytokinesis 10	1.499	0.0004
CLEC5A	C-type lectin domain family 5 member A	2.760	0.0004
KLHL6	kelch like family member 6	1.220	0.0004
HAP1	huntingtin associated protein 1	-1.226	0.0004
FCGR2B	Fc fragment of IgG receptor IIb	2.912	0.0004
EXPH5	exophilin 5	-1.669	0.0004
RASAL3	RAS protein activator like 3	0.853	0.0004
RNASE6	ribonuclease A family member k6	2.481	0.0004
MAOA	monoamine oxidase A	-1.599	0.0004
FCGR2A	Fc fragment of IgG receptor IIa	2.766	0.0004
C1orf162	chromosome 1 open reading frame 162	2.144	0.0004
JAML	junction adhesion molecule like	2.687	0.0004
RUBCNL	RUN and cysteine rich domain containing beclin 1 interacting pro	1.049	0.0004
ARHGEF6	Rac/Cdc42 guanine nucleotide exchange factor 6	1.132	0.0004

IDO1	indoleamine 2,3-dioxygenase 1	-2.231	0.0004
ARL4C	ADP ribosylation factor like GTPase 4C	1.000	0.0004
PSAP	prosaposin	1.017	0.0004
PRKCB	protein kinase C beta	1.912	0.0004
XAF1	XIAP associated factor 1	1.374	0.0004
MYO5C	myosin VC	-1.642	0.0004
CTSS	cathepsin S	2.237	0.0004
IDS	iduronate 2-sulfatase	0.995	0.0004
UBE2D2	ubiquitin conjugating enzyme E2 D2	-0.756	0.0005
GIMAP1-GIMAP5	GIMAP1-GIMAP5 readthrough	1.530	0.0005
APBB1IP	amyloid beta precursor protein binding family B member 1 interac	1.551	0.0005
CDCA7	cell division cycle associated 7	-1.066	0.0005
CELF2	CUGBP, Elav-like family member 2	1.419	0.0005
SLC1A1	solute carrier family 1 member 1	-2.084	0.0005
RHOH	ras homolog family member H	1.093	0.0005
BBOF1	basal body orientation factor 1	-0.958	0.0005
FCGR3B	Fc fragment of IgG receptor IIIb	3.009	0.0005
FCGR3A	Fc fragment of IgG receptor IIIa	2.665	0.0005
ACSL4	acyl-CoA synthetase long-chain family member 4	-1.169	0.0005
ATXN10	ataxin 10	-0.758	0.0005
SCARNA17	small Cajal body-specific RNA 17	1.153	0.0005
OSGIN2	oxidative stress induced growth inhibitor family member 2	0.955	0.0005
HLA-DQA2	major histocompatibility complex, class II, DQ alpha 2	2.673	0.0005
HLA-DQA2	major histocompatibility complex, class II, DQ alpha 2	2.673	0.0005
CLEC7A	C-type lectin domain family 7 member A	2.387	0.0005
RPF2	ribosome production factor 2 homolog	-1.402	0.0005
CD28	CD28 molecule	1.305	0.0005
ALDOC	aldolase, fructose-bisphosphate C	1.189	0.0005
NDUFC2-KCTD14	NDUFC2-KCTD14 readthrough	-0.707	0.0005
CHST15	carbohydrate sulfotransferase 15	1.751	0.0005
CXCL16	C-X-C motif chemokine ligand 16	2.076	0.0005
HLA-DMA	major histocompatibility complex, class II, DM alpha	1.569	0.0005
HLA-DMA	major histocompatibility complex, class II, DM alpha	1.569	0.0005
TIMP2	TIMP metallopeptidase inhibitor 2	1.092	0.0006
MEF2C	myocyte enhancer factor 2C	1.040	0.0006
CTNNAL1	catenin alpha like 1	-1.388	0.0006
LASP1	LIM and SH3 protein 1	0.765	0.0006
PRSS8	protease, serine 8	-0.687	0.0006
HERC5	HECT and RLD domain containing E3 ubiquitin protein ligase 5	1.509	0.0006
LYVE1	lymphatic vessel endothelial hyaluronan receptor 1	2.217	0.0006
DPYD	dihydropyrimidine dehydrogenase	1.185	0.0006
HLA-DMA	major histocompatibility complex, class II, DM alpha	1.537	0.0006
FAM134B	family with sequence similarity 134 member B	-0.910	0.0006
RNF180	ring finger protein 180	-1.608	0.0006
FAM216B	family with sequence similarity 216 member B	-1.136	0.0006

HLA-DRB3	major histocompatibility complex, class II, DR beta 3	1.674	0.0006
RAC2	ras-related C3 botulinum toxin substrate 2 (rho family, small GTF	1.354	0.0006
S100P	S100 calcium binding protein P	-2.550	0.0006
SIRPA	signal regulatory protein alpha	1.092	0.0006
CRISP2	cysteine rich secretory protein 2	-1.257	0.0006
SELPLG	selectin P ligand	1.629	0.0006
RAB11A	RAB11A, member RAS oncogene family	-1.032	0.0006
SLC35A3	solute carrier family 35 member A3	-0.739	0.0006
CHI3L1	chitinase 3 like 1	2.120	0.0006
CD48	CD48 molecule	2.156	0.0006
MYO1G	myosin IG	1.257	0.0006
MIA2	melanoma inhibitory activity 2	-0.693	0.0006
ACADSB	acyl-CoA dehydrogenase, short/branched chain	-1.032	0.0006
TYROBP	TYRO protein tyrosine kinase binding protein	1.582	0.0006
TTC39A	tetratricopeptide repeat domain 39A	-0.609	0.0006
WAS	Wiskott-Aldrich syndrome	1.132	0.0006
TNFAIP8L2	TNF alpha induced protein 8 like 2	0.619	0.0006
HBB	hemoglobin subunit beta	2.277	0.0006
PPBP	pro-platelet basic protein	3.418	0.0006
LRRK2	leucine rich repeat kinase 2	2.071	0.0006
GPR160	G protein-coupled receptor 160	-1.643	0.0006
LRRC8E	leucine rich repeat containing 8 family member E	-0.752	0.0006
CCDC39	coiled-coil domain containing 39	-1.073	0.0007
PIK3CG	phosphatidylinositol-4,5-bisphosphate 3-kinase catalytic subunit	1.291	0.0007
CD180	CD180 molecule	1.753	0.0007
LGALS9C	galectin 9C	1.272	0.0007
IL17RA	interleukin 17 receptor A	1.265	0.0007
LITAF	lipopolysaccharide induced TNF factor	1.095	0.0007
HBA2	hemoglobin subunit alpha 2	4.022	0.0007
HBA2	hemoglobin subunit alpha 2	4.022	0.0007
AIF1	allograft inflammatory factor 1	1.744	0.0007
AIF1	allograft inflammatory factor 1	1.744	0.0007
AIF1	allograft inflammatory factor 1	1.744	0.0007
BCO1	beta-carotene oxygenase 1	-1.178	0.0007
CTH	cystathionine gamma-lyase	-1.024	0.0007
RPS6KA5	ribosomal protein S6 kinase A5	-1.794	0.0007
FAM65B	family with sequence similarity 65 member B	1.869	0.0007
KCNK1	potassium two pore domain channel subfamily K member 1	-1.206	0.0007
TMEM181	transmembrane protein 181	-0.572	0.0007
C19orf33	chromosome 19 open reading frame 33	-1.104	0.0007
STK10	serine/threonine kinase 10	1.141	0.0007
SLC7A7	solute carrier family 7 member 7	1.538	0.0007
TLR2	toll like receptor 2	1.701	0.0007
MMP9	matrix metallopeptidase 9	2.600	0.0007
CYTOR	cytoskeleton regulator RNA	0.698	0.0007

FMNL1	formin like 1	1.213	0.0007
LYZ	lysozyme	2.854	0.0007
TGFBI	transforming growth factor beta induced	1.677	0.0007
C5AR1	complement C5a receptor 1	1.939	0.0007
COA3	cytochrome c oxidase assembly factor 3	-0.649	0.0007
STK4	serine/threonine kinase 4	1.136	0.0007
RIN3	Ras and Rab interactor 3	0.747	0.0007
IL2RG	interleukin 2 receptor subunit gamma	2.175	0.0007
ZNF626	zinc finger protein 626	-0.807	0.0007
CEACAM1	carcinoembryonic antigen related cell adhesion molecule 1	-1.386	0.0007
PLCXD3	phosphatidylinositol specific phospholipase C X domain containi	-1.462	0.0007
BCAS1	breast carcinoma amplified sequence 1	-0.765	0.0007
CEP70	centrosomal protein 70	-1.574	0.0007
HCK	HCK proto-oncogene, Src family tyrosine kinase	1.512	0.0007
TST	thiosulfate sulfurtransferase	-1.086	0.0007
ITGB2	integrin subunit beta 2	1.646	0.0007
ANKRD20A2	ankyrin repeat domain 20 family member A2	-1.108	0.0007
FUT2	fucosyltransferase 2	-1.311	0.0008
CLEC4A	C-type lectin domain family 4 member A	1.498	0.0008
RAB31	RAB31, member RAS oncogene family	1.125	0.0008
BHLHE40	basic helix-loop-helix family member e40	1.346	0.0008
FCGR1A	Fc fragment of IgG receptor Ia	1.977	0.0008
MPP1	membrane palmitoylated protein 1	1.834	0.0008
PRL	prolactin	2.723	0.0008
GCSH	glycine cleavage system protein H	-1.460	0.0008
WDFY4	WDFY family member 4	0.747	0.0008
RASEF	RAS and EF-hand domain containing	-1.446	0.0008
SERPINE1	serpin family E member 1	1.689	0.0008
MMP19	matrix metallopeptidase 19	1.343	0.0008
TGFB1	transforming growth factor beta 1	1.283	0.0008
BCAP29	B-cell receptor associated protein 29	-0.898	0.0008
HEY1	hes related family bHLH transcription factor with YRPW motif 1	-1.423	0.0008
PDE4DIP	phosphodiesterase 4D interacting protein	0.805	0.0008
FRY	FRY microtubule binding protein	1.092	0.0008
FYB	FYN binding protein	2.167	0.0008
SLC44A1	solute carrier family 44 member 1	-1.574	0.0008
CDH1	cadherin 1	-1.487	0.0008
ST8SIA4	ST8 alpha-N-acetyl-neuraminide alpha-2,8-sialyltransferase 4	1.451	0.0008
OGN	osteoglycin	-2.138	0.0008
S100A16	S100 calcium binding protein A16	-0.877	0.0008
FCN1	ficolin 1	1.111	0.0008
SSH2	slingshot protein phosphatase 2	1.240	0.0008
FCGR1A	Fc fragment of IgG receptor Ia	1.933	0.0008
ITGB7	integrin subunit beta 7	0.879	0.0008
TRAF1	TNF receptor associated factor 1	0.673	0.0008

LEFTY2	left-right determination factor 2	0.941	0.0008
AP1M2	adaptor related protein complex 1 mu 2 subunit	-0.846	0.0008
PLA2G10	phospholipase A2 group X	-2.889	0.0008
ITGB3	integrin subunit beta 3	1.532	0.0009
INHBA	inhibin beta A subunit	1.384	0.0009
CD68	CD68 molecule	2.188	0.0009
BIN2	bridging integrator 2	1.698	0.0009
LGALS9C	galectin 9C	1.219	0.0009
ARHGEF2	Rho/Rac guanine nucleotide exchange factor 2	0.645	0.0009
ICA1	islet cell autoantigen 1	-1.337	0.0009
ANKRD20A3	ankyrin repeat domain 20 family member A3	-1.410	0.0009
DHRS9	dehydrogenase/reductase 9	1.238	0.0009
QPCT	glutaminyl-peptide cyclotransferase	0.773	0.0009
LPXN	leupaxin	1.290	0.0009
EFCAB1	EF-hand calcium binding domain 1	-1.226	0.0009
MAFB	MAF bZIP transcription factor B	1.415	0.0009
MPC1	mitochondrial pyruvate carrier 1	-0.801	0.0009
TRIM21	tripartite motif containing 21	0.840	0.0009
C1QC	complement C1q C chain	1.730	0.0009
NCF2	neutrophil cytosolic factor 2	2.428	0.0009
APOBEC4	apolipoprotein B mRNA editing enzyme catalytic polypeptide like	-0.867	0.0009
TMEM106A	transmembrane protein 106A	1.148	0.0009
SFRP1	secreted frizzled related protein 1	-1.103	0.0009
PPP1R18	protein phosphatase 1 regulatory subunit 18	0.831	0.0009
TLCD1	TLC domain containing 1	-0.746	0.0009
CELF2-AS1	CELF2 antisense RNA 1	1.160	0.0009
ITGAL	integrin subunit alpha L	1.101	0.0009
BCL2	BCL2, apoptosis regulator	0.583	0.0009
FCER1G	Fc fragment of IgE receptor Ig	2.340	0.0009
LEO1	LEO1 homolog, Paf1/RNA polymerase II complex component	-1.009	0.0009
CYTH4	cytohesin 4	1.298	0.0009
SEMA7A	semaphorin 7A (John Milton Hagen blood group)	0.644	0.0009
ARHGAP25	Rho GTPase activating protein 25	1.431	0.0009
MOGAT1	monoacylglycerol O-acyltransferase 1	-1.780	0.0009
SLC16A10	solute carrier family 16 member 10	1.953	0.0009
MARVELD2	MARVEL domain containing 2	-1.626	0.0009
MARVELD2	MARVEL domain containing 2	-1.626	0.0009
MS4A6A	membrane spanning 4-domains A6A	1.519	0.0009
GNG8	G protein subunit gamma 8	0.959	0.0009
DERA	deoxyribose-phosphate aldolase	-0.922	0.0009
ISOC1	isochorismatase domain containing 1	-0.708	0.0010
PECAM1	platelet and endothelial cell adhesion molecule 1	1.164	0.0010
TSPAN13	tetraspanin 13	-0.907	0.0010
FCHSD2	FCH and double SH3 domains 2	0.826	0.0010
SLAIN1	SLAIN motif family member 1	-1.436	0.0010

HEBP2	heme binding protein 2	-0.817	0.0010
GUCY1A3	guanylate cyclase 1 soluble subunit alpha	-0.951	0.0010
RAB8B	RAB8B, member RAS oncogene family	1.097	0.0010
GK	glycerol kinase	1.593	0.0010
GPR84	G protein-coupled receptor 84	1.754	0.0010
IL33	interleukin 33	-1.370	0.0010
C7orf13	chromosome 7 open reading frame 13	0.555	0.0010
TMEM106A	transmembrane protein 106A	1.140	0.0010
PTN	pleiotrophin	-2.352	0.0010
CST6	cystatin E/M	1.692	0.0010
TSPAN15	tetraspanin 15	-0.782	0.0010
CD1C	CD1c molecule	1.000	0.0010
RASSF6	Ras association domain family member 6	-0.859	0.0010
MRPL32	mitochondrial ribosomal protein L32	-0.645	0.0011
FLCN	folliculin	0.660	0.0011
DNAAF2	dynein axonemal assembly factor 2	-0.714	0.0011
GPR141	G protein-coupled receptor 141	1.493	0.0011
GLO1	glyoxalase I	-0.564	0.0011
HEY2	hes related family bHLH transcription factor with YRPW motif 2	-1.149	0.0011
OTUB2	OTU deubiquitinase, ubiquitin aldehyde binding 2	-0.662	0.0011
SLC43A3	solute carrier family 43 member 3	1.448	0.0011
SLC9A9	solute carrier family 9 member A9	0.826	0.0011
C2orf88	chromosome 2 open reading frame 88	-1.127	0.0011
SHROOM3	shroom family member 3	-1.561	0.0011
TMEM196	transmembrane protein 196	-1.732	0.0011
SLC2A3	solute carrier family 2 member 3	2.357	0.0011
MYO1F	myosin IF	1.702	0.0011
ENTPD3	ectonucleoside triphosphate diphosphohydrolase 3	-1.888	0.0011
CD37	CD37 molecule	1.659	0.0011
ZBBX	zinc finger B-box domain containing	-1.522	0.0011
PIK3R5	phosphoinositide-3-kinase regulatory subunit 5	1.179	0.0011
ICAM1	intercellular adhesion molecule 1	1.242	0.0011
NDUFB4	NADH:ubiquinone oxidoreductase subunit B4	-0.665	0.0011
NDUFB4	NADH:ubiquinone oxidoreductase subunit B4	-0.665	0.0011
MNAT1	MNAT1, CDK activating kinase assembly factor	-0.832	0.0011
GK	glycerol kinase	1.506	0.0011
MUC15	mucin 15, cell surface associated	-2.234	0.0012
TNFAIP3	TNF alpha induced protein 3	0.857	0.0012
CYSLTR1	cysteinyl leukotriene receptor 1	1.198	0.0012
PLLP	plasmolipin	-0.990	0.0012
CARD8	caspase recruitment domain family member 8	0.803	0.0012
NFATC2	nuclear factor of activated T-cells 2	1.059	0.0012
CD226	CD226 molecule	1.604	0.0012
HLA-DPB1	major histocompatibility complex, class II, DP beta 1	1.577	0.0012
ENTPD5	ectonucleoside triphosphate diphosphohydrolase 5	-0.917	0.0012

CD83	CD83 molecule	1.362	0.0012
CMKLR1	chemerin chemokine-like receptor 1	1.375	0.0012
CASZ1	castor zinc finger 1	-1.384	0.0012
SCGB2A2	secretoglobin family 2A member 2	-2.503	0.0012
NCAM2	neural cell adhesion molecule 2	-0.907	0.0012
ABCA5	ATP binding cassette subfamily A member 5	-0.853	0.0012
AGPAT5	1-acylglycerol-3-phosphate O-acyltransferase 5	-1.406	0.0012
CR1	complement C3b/C4b receptor 1 (Knops blood group)	1.875	0.0012
P2RY10	purinergic receptor P2Y10	1.894	0.0012
B4GALT4	beta-1,4-galactosyltransferase 4	-1.016	0.0012
PLAUR	plasminogen activator, urokinase receptor	1.469	0.0012
STARD4	StAR related lipid transfer domain containing 4	0.954	0.0012
STAT5A	signal transducer and activator of transcription 5A	0.659	0.0012
GBP4	guanylate binding protein 4	0.846	0.0012
RPF2	ribosome production factor 2 homolog	-1.347	0.0012
CREB3L1	cAMP responsive element binding protein 3 like 1	-1.418	0.0012
IFIT2	interferon induced protein with tetratricopeptide repeats 2	1.662	0.0012
SESTD1	SEC14 and spectrin domain containing 1	-1.437	0.0013
LGALS9	galectin 9	1.089	0.0013
NPC2	NPC intracellular cholesterol transporter 2	1.236	0.0013
PLEKHO1	pleckstrin homology domain containing O1	1.038	0.0013
HLA-DOA	major histocompatibility complex, class II, DO alpha	1.128	0.0013
CTSB	cathepsin B	1.276	0.0013
SYK	spleen associated tyrosine kinase	1.202	0.0013
GCNT2	glucosaminyl (N-acetyl) transferase 2, I-branching enzyme (I bloc	-1.073	0.0013
EMP3	epithelial membrane protein 3	1.291	0.0013
ECI1	enoyl-CoA delta isomerase 1	-0.744	0.0014
NPL	N-acetylneuraminate pyruvate lyase	1.980	0.0014
FAM105A	family with sequence similarity 105 member A	0.818	0.0014
ZNF165	zinc finger protein 165	-0.717	0.0014
TAPBP	TAP binding protein	0.664	0.0014
PLEK	pleckstrin	1.981	0.0014
CTSZ	cathepsin Z	1.024	0.0014
IL1B	interleukin 1 beta	1.970	0.0014
LY75-CD302	LY75-CD302 readthrough	0.551	0.0014
PRIMA1	proline rich membrane anchor 1	0.652	0.0014
CBL	Cbl proto-oncogene	0.962	0.0014
TNFRSF1B	TNF receptor superfamily member 1B	0.952	0.0014
CYB5B	cytochrome b5 type B	-1.231	0.0014
ABHD17C	abhydrolase domain containing 17C	-0.704	0.0014
SLC11A1	solute carrier family 11 member 1	2.019	0.0014
POP7	POP7 homolog, ribonuclease P/MRP subunit	-0.513	0.0014
ARHGAP30	Rho GTPase activating protein 30	1.176	0.0014
DOCK4	dedicator of cytokinesis 4	1.058	0.0014
ANPEP	alanyl aminopeptidase, membrane	1.795	0.0014

ADGRE4P	adhesion G protein-coupled receptor E4, pseudogene	0.883	0.0014
STX18	syntaxin 18	-1.231	0.0014
SYT14	synaptotagmin 14	-1.552	0.0015
KRT19	keratin 19	-1.353	0.0015
THBS1	thrombospondin 1	1.611	0.0015
PIK3AP1	phosphoinositide-3-kinase adaptor protein 1	1.484	0.0015
C9orf135	chromosome 9 open reading frame 135	-1.235	0.0015
CYFIP2	cytoplasmic FMR1 interacting protein 2	1.222	0.0015
LGALS2	galectin 2	1.799	0.0015
MERTK	MER proto-oncogene, tyrosine kinase	0.946	0.0015
PGR	progesterone receptor	-1.251	0.0015
C4BPA	complement component 4 binding protein alpha	-2.233	0.0015
HLA-DQA2	major histocompatibility complex, class II, DQ alpha 2	2.036	0.0015
GOT1	glutamic-oxaloacetic transaminase 1	-1.032	0.0015
MSN	moesin	1.002	0.0016
GPR37	G protein-coupled receptor 37	-0.755	0.0016
MAPK6	mitogen-activated protein kinase 6	-1.054	0.0016
NDUFA2	NADH:ubiquinone oxidoreductase subunit A2	-0.790	0.0016
ADH5	alcohol dehydrogenase 5 (class III), chi polypeptide	-0.804	0.0016
DYNLT1	dynein light chain Tctex-type 1	-0.768	0.0016
MCU	mitochondrial calcium uniporter	-0.837	0.0016
PARD6B	par-6 family cell polarity regulator beta	-0.890	0.0016
CAPZA2	capping actin protein of muscle Z-line alpha subunit 2	-1.242	0.0016
LCP1	lymphocyte cytosolic protein 1	1.537	0.0016
PLSCR1	phospholipid scramblase 1	0.983	0.0016
PTPN22	protein tyrosine phosphatase, non-receptor type 22	1.600	0.0016
HLA-DQA2	major histocompatibility complex, class II, DQ alpha 2	2.000	0.0016
WWC1	WW and C2 domain containing 1	-0.955	0.0016
APH1B	aph-1 homolog B, gamma-secretase subunit	0.793	0.0017
AQP9	aquaporin 9	2.335	0.0017
MAPKAPK3	mitogen-activated protein kinase-activated protein kinase 3	1.096	0.0017
TNFSF8	tumor necrosis factor superfamily member 8	1.169	0.0017
PSTPIP1	proline-serine-threonine phosphatase interacting protein 1	0.778	0.0017
FKBP11	FK506 binding protein 11	-0.775	0.0017
PTPN6	protein tyrosine phosphatase, non-receptor type 6	1.103	0.0017
SGIP1	SH3 domain GRB2 like endophilin interacting protein 1	1.322	0.0017
PARP14	poly(ADP-ribose) polymerase family member 14	0.967	0.0017
IRX3	iroquois homeobox 3	-0.932	0.0017
EFCAB10	EF-hand calcium binding domain 10	-1.031	0.0017
NWD1	NACHT and WD repeat domain containing 1	-0.680	0.0017
GMFG	glia maturation factor gamma	1.660	0.0017
UQCRQ	ubiquinol-cytochrome c reductase complex III subunit VII	-0.731	0.0017
ANKRD20A2	ankyrin repeat domain 20 family member A2	-1.059	0.0017
ANKRD20A2	ankyrin repeat domain 20 family member A2	-1.059	0.0017
BTN2A2	butyrophilin subfamily 2 member A2	0.586	0.0017

ARFGEF3	ARFGEF family member 3	-0.608	0.0017
MT2A	metallothionein 2A	-1.066	0.0017
HLA-DPB1	major histocompatibility complex, class II, DP beta 1	1.801	0.0017
SHISA5	shisa family member 5	0.641	0.0017
C9orf66	chromosome 9 open reading frame 66	1.365	0.0017
GHITM	growth hormone inducible transmembrane protein	-1.035	0.0017
HLA-F	major histocompatibility complex, class I, F	0.815	0.0017
TMEM71	transmembrane protein 71	1.914	0.0017
NPTX1	neuronal pentraxin 1	0.515	0.0018
ITGA5	integrin subunit alpha 5	1.018	0.0018
SIGLEC14	sialic acid binding Ig like lectin 14	0.874	0.0018
AIM2	absent in melanoma 2	1.086	0.0018
HLX	H2.0 like homeobox	0.655	0.0018
USMG5	up-regulated during skeletal muscle growth 5 homolog (mouse)	-0.821	0.0018
GULP1	GULP, engulfment adaptor PTB domain containing 1	-1.119	0.0018
PIEZO1	piezo type mechanosensitive ion channel component 1	0.652	0.0018
OAS3	2'-5'-oligoadenylate synthetase 3	1.225	0.0018
NCKAP1	NCK associated protein 1	-1.145	0.0018
EPB41L3	erythrocyte membrane protein band 4.1 like 3	1.112	0.0018
HSD11B1	hydroxysteroid 11-beta dehydrogenase 1	2.230	0.0018
SLC23A2	solute carrier family 23 member 2	0.769	0.0018
PHOSPHO1	phosphoethanolamine/phosphocholine phosphatase	0.935	0.0019
ZBP1	Z-DNA binding protein 1	0.704	0.0019
FCMR	Fc fragment of IgM receptor	1.689	0.0019
FLI1	Fli-1 proto-oncogene, ETS transcription factor	1.192	0.0019
FAM49A	family with sequence similarity 49 member A	1.106	0.0019
NAALADL2	N-acetylated alpha-linked acidic dipeptidase like 2	-1.209	0.0019
RTKN	rhotekin	-0.502	0.0019
PHYHD1	phytanoyl-CoA dioxygenase domain containing 1	-0.700	0.0019
BTK	Bruton tyrosine kinase	1.215	0.0019
MRPL3	mitochondrial ribosomal protein L3	-0.697	0.0019
HLA-DRB1	major histocompatibility complex, class II, DR beta 1	1.622	0.0019
PTPRC	protein tyrosine phosphatase, receptor type C	2.047	0.0019
TMEM141	transmembrane protein 141	-0.936	0.0019
ZFP1	ZFP1 zinc finger protein	-0.577	0.0020
CPEB4	cytoplasmic polyadenylation element binding protein 4	0.624	0.0020
FAIM	Fas apoptotic inhibitory molecule	-1.044	0.0020
IRF8	interferon regulatory factor 8	1.100	0.0020
MINA	MYC induced nuclear antigen	-0.485	0.0020
RHOU	ras homolog family member U	-0.803	0.0021
LRRC8C	leucine rich repeat containing 8 family member C	0.825	0.0021
TAPBP	TAP binding protein	0.723	0.0021
FHDC1	FH2 domain containing 1	-0.896	0.0021
C1QA	complement C1q A chain	1.616	0.0021
MRPL19	mitochondrial ribosomal protein L19	-0.693	0.0021

MS4A4A	membrane spanning 4-domains A4A	1.797	0.0021
CXorf21	chromosome X open reading frame 21	1.342	0.0021
YARS2	tyrosyl-tRNA synthetase 2	-0.659	0.0021
SLCO2B1	solute carrier organic anion transporter family member 2B1	1.349	0.0021
USP47	ubiquitin specific peptidase 47	-1.086	0.0022
CMYA5	cardiomyopathy associated 5	-0.751	0.0022
ANKRD35	ankyrin repeat domain 35	-1.102	0.0022
MAR1	membrane associated ring-CH-type finger 1	1.547	0.0022
SP110	SP110 nuclear body protein	1.108	0.0022
MR1	major histocompatibility complex, class I-related	0.839	0.0022
GTF3C6	general transcription factor IIIC subunit 6	-0.808	0.0022
MAL2	mal, T-cell differentiation protein 2 (gene/pseudogene)	-1.283	0.0022
ENO2	enolase 2	1.074	0.0022
CD40	CD40 molecule	1.021	0.0022
NEU1	neuraminidase 1	-1.335	0.0022
NEU1	neuraminidase 1	-1.335	0.0022
NEU1	neuraminidase 1	-1.335	0.0022
RASSF5	Ras association domain family member 5	1.164	0.0022
ARHGAP26	Rho GTPase activating protein 26	1.527	0.0022
IL3RA	interleukin 3 receptor subunit alpha	1.186	0.0022
IL3RA	interleukin 3 receptor subunit alpha	1.186	0.0022
TMEM136	transmembrane protein 136	-1.128	0.0022
LRRFIP1	LRR binding FLII interacting protein 1	1.433	0.0022
MRAS	muscle RAS oncogene homolog	0.915	0.0022
STX19	syntaxin 19	-1.150	0.0022
ELOVL7	ELOVL fatty acid elongase 7	-0.853	0.0023
ELK4	ELK4, ETS transcription factor	-0.769	0.0023
CHST11	carbohydrate sulfotransferase 11	1.215	0.0023
IFIT3	interferon induced protein with tetratricopeptide repeats 3	1.277	0.0023
FGD2	FYVE, RhoGEF and PH domain containing 2	0.821	0.0023
MS4A1	membrane spanning 4-domains A1	1.847	0.0023
BACE2	beta-site APP-cleaving enzyme 2	-1.187	0.0023
ZNF28	zinc finger protein 28	-0.755	0.0023
LILRB1	leukocyte immunoglobulin like receptor B1	0.930	0.0023
HLA-F	major histocompatibility complex, class I, F	0.812	0.0024
STAB1	stabilin 1	0.619	0.0024
ZNF876P	zinc finger protein 876, pseudogene	-0.799	0.0024
FGF7	fibroblast growth factor 7	-1.258	0.0024
FGF7	fibroblast growth factor 7	-1.258	0.0024
FGF7	fibroblast growth factor 7	-1.258	0.0024
FTL	ferritin light chain	1.447	0.0024
THOC7	THO complex 7	-0.663	0.0024
PARVG	parvin gamma	1.116	0.0024
INPP5D	inositol polyphosphate-5-phosphatase D	1.292	0.0024
PRELID3B	PRELI domain containing 3B	-0.643	0.0024

ATF5	activating transcription factor 5	0.737	0.0024
USP53	ubiquitin specific peptidase 53	-1.559	0.0024
ADCY7	adenylate cyclase 7	0.704	0.0024
ETV5	ETS variant 5	0.919	0.0024
EFHD2	EF-hand domain family member D2	0.986	0.0024
VAV3	vav guanine nucleotide exchange factor 3	1.181	0.0024
ANKRD20A2	ankyrin repeat domain 20 family member A2	-1.013	0.0024
TBC1D14	TBC1 domain family member 14	0.819	0.0024
PSMG1	proteasome assembly chaperone 1	-0.618	0.0024
TMEM101	transmembrane protein 101	-1.314	0.0024
CCR1	C-C motif chemokine receptor 1	1.805	0.0024
NRROS	negative regulator of reactive oxygen species	0.907	0.0025
SGPP2	sphingosine-1-phosphate phosphatase 2	-0.918	0.0025
FGL1	fibrinogen like 1	-1.168	0.0025
ADAR	adenosine deaminase, RNA specific	0.683	0.0025
DCTPP1	dCTP pyrophosphatase 1	-0.872	0.0025
NLGN1	neuroligin 1	-1.099	0.0025
PTP4A1	protein tyrosine phosphatase type IVA, member 1	-0.932	0.0025
ARHGAP9	Rho GTPase activating protein 9	1.240	0.0025
HLA-F	major histocompatibility complex, class I, F	0.817	0.0026
IGF2R	insulin like growth factor 2 receptor	0.711	0.0026
IFIH1	interferon induced with helicase C domain 1	0.753	0.0026
CAB39L	calcium binding protein 39 like	1.985	0.0026
CSF3R	colony stimulating factor 3 receptor	1.876	0.0026
LOC653653	adaptor related protein complex 1 sigma 2 subunit pseudogene	1.043	0.0026
PGA3	pepsinogen 3, group I (pepsinogen A)	0.756	0.0026
HLA-DPB1	major histocompatibility complex, class II, DP beta 1	1.530	0.0026
ESM1	endothelial cell specific molecule 1	-0.587	0.0026
NEDD9	neural precursor cell expressed, developmentally down-regulated	1.070	0.0026
SDR42E1	short chain dehydrogenase/reductase family 42E, member 1	-0.586	0.0026
IKZF1	IKAROS family zinc finger 1	1.373	0.0026
ISG15	ISG15 ubiquitin-like modifier	0.882	0.0026
FN1	fibronectin 1	1.785	0.0026
MSMO1	methylsterol monooxygenase 1	-0.886	0.0026
SRGN	serglycin	1.735	0.0027
GXYLT2	glucoside xylosyltransferase 2	-1.585	0.0027
ARAP1	ArfGAP with RhoGAP domain, ankyrin repeat and PH domain 1	0.726	0.0027
CD84	CD84 molecule	1.677	0.0027
LST1	leukocyte specific transcript 1	1.766	0.0027
LST1	leukocyte specific transcript 1	1.766	0.0027
LST1	leukocyte specific transcript 1	1.766	0.0027
PAK1IP1	PAK1 interacting protein 1	-0.948	0.0027
ID2	inhibitor of DNA binding 2, HLH protein	-0.667	0.0027
EVI2B	ecotropic viral integration site 2B	2.018	0.0027
MRPL37	mitochondrial ribosomal protein L37	-0.656	0.0027

FBXO36	F-box protein 36	-0.588	0.0027
LTBP2	latent transforming growth factor beta binding protein 2	0.704	0.0027
HMGB3	high mobility group box 3	-0.695	0.0027
SYT1	synaptotagmin 1	0.791	0.0027
LILRA6	leukocyte immunoglobulin like receptor A6	1.619	0.0028
NIPSNAP3A	nipsnap homolog 3A	-1.129	0.0028
DAPP1	dual adaptor of phosphotyrosine and 3-phosphoinositides 1	1.546	0.0028
ATP5I	ATP synthase, H+ transporting, mitochondrial Fo complex subur	-0.458	0.0028
C6orf52	chromosome 6 open reading frame 52	-0.736	0.0028
SIGLEC9	sialic acid binding Ig like lectin 9	0.968	0.0028
IDO2	indoleamine 2,3-dioxygenase 2	-2.431	0.0028
COLEC12	collectin subfamily member 12	1.279	0.0028
ITSN2	intersectin 2	0.724	0.0029
PHF1	PHD finger protein 1	0.570	0.0029
GBP5	guanylate binding protein 5	1.676	0.0029
C11orf52	chromosome 11 open reading frame 52	-0.655	0.0029
HLA-DQB2	major histocompatibility complex, class II, DQ beta 2	1.405	0.0029
HLA-DQB2	major histocompatibility complex, class II, DQ beta 2	1.405	0.0029
UVRAG	UV radiation resistance associated	0.806	0.0030
DYNLRB2	dynein light chain roadblock-type 2	-1.063	0.0030
SECTM1	secreted and transmembrane 1	0.791	0.0030
FPR1	formyl peptide receptor 1	2.275	0.0030
TM9SF3	transmembrane 9 superfamily member 3	-0.563	0.0030
LAT	linker for activation of T-cells	0.699	0.0030
RUNX1	runt related transcription factor 1	0.814	0.0030
CD55	CD55 molecule (Cromer blood group)	-0.714	0.0030
EPC1	enhancer of polycomb homolog 1	1.757	0.0031
RNF213	ring finger protein 213	0.963	0.0031
HLA-DRB1	major histocompatibility complex, class II, DR beta 1	1.657	0.0031
HBD	hemoglobin subunit delta	1.726	0.0031
MRPL15	mitochondrial ribosomal protein L15	-0.950	0.0031
DEF6	DEF6, guanine nucleotide exchange factor	0.766	0.0031
HLA-F	major histocompatibility complex, class I, F	0.629	0.0031
GPAT3	glycerol-3-phosphate acyltransferase 3	0.712	0.0031
SPP1	secreted phosphoprotein 1	1.737	0.0031
SLC22A15	solute carrier family 22 member 15	0.744	0.0032
HIST1H2AC	histone cluster 1 H2A family member c	-0.662	0.0032
GLUL	glutamate-ammonia ligase	0.963	0.0032
GAS7	growth arrest specific 7	0.563	0.0032
HN1	hematological and neurological expressed 1	-0.614	0.0032
TCIRG1	T-cell immune regulator 1, ATPase H+ transporting V0 subunit a3	0.640	0.0032
TM6SF1	transmembrane 6 superfamily member 1	0.929	0.0032
TMEM260	transmembrane protein 260	-0.849	0.0032
TAGAP	T-cell activation RhoGTPase activating protein	1.625	0.0032
ELP3	elongator acetyltransferase complex subunit 3	-1.528	0.0032

C10orf54	chromosome 10 open reading frame 54	0.609	0.0033
ESRP1	epithelial splicing regulatory protein 1	-1.577	0.0033
PGA3	pepsinogen 3, group I (pepsinogen A)	0.821	0.0033
RASGRP2	RAS guanyl releasing protein 2	0.788	0.0033
CORO1C	coronin 1C	0.951	0.0033
GIMAP7	GTPase, IMAP family member 7	1.289	0.0033
UPP1	uridine phosphorylase 1	0.963	0.0033
LYPLA1	lysophospholipase I	-0.682	0.0033
GPD1L	glycerol-3-phosphate dehydrogenase 1-like	-0.725	0.0033
LPIN3	lipin 3	-0.696	0.0033
MRPL13	mitochondrial ribosomal protein L13	-0.908	0.0033
N4BP2L1	NEDD4 binding protein 2 like 1	1.116	0.0033
POC1B-GALNT4	POC1B-GALNT4 readthrough	-1.088	0.0033
NDUFA7	NADH:ubiquinone oxidoreductase subunit A7	-0.618	0.0033
CCR7	C-C motif chemokine receptor 7	1.347	0.0033
RAB29	RAB29, member RAS oncogene family	0.733	0.0033
REL	REL proto-oncogene, NF-kB subunit	0.759	0.0033
HAVCR2	hepatitis A virus cellular receptor 2	1.210	0.0034
TIGIT	T-cell immunoreceptor with Ig and ITIM domains	0.808	0.0034
MPZL2	myelin protein zero like 2	-1.667	0.0034
ANKRD44	ankyrin repeat domain 44	1.157	0.0034
ADAMTS19	ADAM metallopeptidase with thrombospondin type 1 motif 19	-0.838	0.0034
TOB1	transducer of ERBB2, 1	-0.855	0.0034
CYTOR	cytoskeleton regulator RNA	0.635	0.0034
MSX1	msh homeobox 1	-0.639	0.0034
NEK10	NIMA related kinase 10	-0.855	0.0035
ZNF770	zinc finger protein 770	-0.462	0.0035
MRPS28	mitochondrial ribosomal protein S28	-1.147	0.0035
RNF213	ring finger protein 213	0.975	0.0035
MFNG	MFNG O-fucosylpeptide 3-beta-N-acetylglucosaminyltransferase	0.490	0.0035
ELK3	ELK3, ETS transcription factor	0.707	0.0035
MCTP2	multiple C2 and transmembrane domain containing 2	0.749	0.0035
PDE3B	phosphodiesterase 3B	1.236	0.0035
SH3BGRL3	SH3 domain binding glutamate rich protein like 3	0.632	0.0035
COPS3	COP9 signalosome subunit 3	-0.432	0.0035
RGS6	regulator of G-protein signaling 6	-0.695	0.0035
TLR8	toll like receptor 8	1.793	0.0036
STAT4	signal transducer and activator of transcription 4	1.206	0.0036
CATSPER2	cation channel sperm associated 2	0.638	0.0036
EPB41L5	erythrocyte membrane protein band 4.1 like 5	-1.113	0.0036
HLA-F	major histocompatibility complex, class I, F	0.838	0.0036
HLA-F	major histocompatibility complex, class I, F	0.838	0.0036
MYADM	myeloid associated differentiation marker	1.015	0.0036
CCL13	C-C motif chemokine ligand 13	2.478	0.0037
GPR183	G protein-coupled receptor 183	1.738	0.0037

LRRFIP1	LRR binding FLII interacting protein 1	1.469	0.0037
RCSD1	RCSD domain containing 1	0.747	0.0037
TLN1	talin 1	0.709	0.0037
TIMM13	translocase of inner mitochondrial membrane 13	-0.607	0.0037
SLC12A6	solute carrier family 12 member 6	0.853	0.0037
FGL2	fibrinogen like 2	1.484	0.0037
ADGRL2	adhesion G protein-coupled receptor L2	-1.404	0.0037
TMEM176A	transmembrane protein 176A	1.719	0.0037
CIITA	class II major histocompatibility complex transactivator	0.608	0.0037
PLXDC1	plexin domain containing 1	0.728	0.0037
CDS1	CDP-diacylglycerol synthase 1	-0.734	0.0037
EPSTI1	epithelial stromal interaction 1 (breast)	1.022	0.0037
GIMAP6	GTPase, IMAP family member 6	0.721	0.0037
PKD1L2	polycystin 1 like 2 (gene/pseudogene)	-0.669	0.0037
SELL	selectin L	2.457	0.0038
TAPBP	TAP binding protein	0.702	0.0038
SLPI	secretory leukocyte peptidase inhibitor	-1.941	0.0038
NAPSA	napsin A aspartic peptidase	0.976	0.0038
C1QB	complement C1q B chain	1.522	0.0038
COQ9	coenzyme Q9	-0.467	0.0038
YAE1D1	Yae1 domain containing 1	-0.806	0.0038
TBCK	TBC1 domain containing kinase	-1.154	0.0038
CACNA1D	calcium voltage-gated channel subunit alpha1 D	-1.353	0.0038
DENND1A	DENN domain containing 1A	0.760	0.0038
FAM198B	family with sequence similarity 198 member B	-1.031	0.0038
NAV1	neuron navigator 1	0.598	0.0038
PTPRR	protein tyrosine phosphatase, receptor type R	-0.896	0.0039
PRDX4	peroxiredoxin 4	-0.916	0.0039
SERF1B	small EDRK-rich factor 1B	-0.782	0.0039
SERF1B	small EDRK-rich factor 1B	-0.782	0.0039
SERF1B	small EDRK-rich factor 1B	-0.782	0.0039
CABYR	calcium binding tyrosine phosphorylation regulated	-0.777	0.0039
MPHOSPH10	M-phase phosphoprotein 10	-0.606	0.0039
LRBA	LPS responsive beige-like anchor protein	-0.797	0.0040
PHYH	phytanoyl-CoA 2-hydroxylase	-0.552	0.0040
TMEM144	transmembrane protein 144	-0.930	0.0041
NLRC4	NLR family CARD domain containing 4	1.238	0.0041
TTYH3	tweety family member 3	0.773	0.0041
CFAP206	cilia and flagella associated protein 206	-0.834	0.0041
UCA1	urothelial cancer associated 1 (non-protein coding)	-1.727	0.0042
LRRC31	leucine rich repeat containing 31	-1.083	0.0042
MAP4K3	mitogen-activated protein kinase kinase kinase kinase 3	-0.701	0.0042
CCR2	C-C motif chemokine receptor 2	1.477	0.0042
ITPR1	inositol 1,4,5-trisphosphate receptor type 1	0.812	0.0042
GRK3	G protein-coupled receptor kinase 3	1.210	0.0042

STMN2	stathmin 2	1.741	0.0042
FGF7	fibroblast growth factor 7	-1.413	0.0042
CD33	CD33 molecule	1.086	0.0042
ZNF433	zinc finger protein 433	-0.654	0.0042
SLAMF6	SLAM family member 6	1.685	0.0043
MYO5B	myosin VB	-0.977	0.0043
TCAF2	TRPM8 channel associated factor 2	0.715	0.0043
LSP1	lymphocyte-specific protein 1	0.682	0.0043
PREX1	phosphatidylinositol-3,4,5-trisphosphate dependent Rac exchan	0.928	0.0043
MBNL1	muscleblind like splicing regulator 1	0.785	0.0043
SNTN	sentan, cilia apical structure protein	-1.358	0.0043
LYN	LYN proto-oncogene, Src family tyrosine kinase	1.553	0.0043
C7orf55-LUC7L2	C7orf55-LUC7L2 readthrough	-0.582	0.0043
COX6A1	cytochrome c oxidase subunit 6A1	-0.521	0.0043
KAT2B	lysine acetyltransferase 2B	0.882	0.0043
HLA-F	major histocompatibility complex, class I, F	0.728	0.0043
GM2A	GM2 ganglioside activator	1.112	0.0043
EFHC2	EF-hand domain containing 2	-0.794	0.0044
CYP2C9	cytochrome P450 family 2 subfamily C member 9	-0.694	0.0044
ZRSR2	zinc finger CCCH-type, RNA binding motif and serine/arginine ric	0.525	0.0044
DNAH12	dynein axonemal heavy chain 12	-1.048	0.0044
SCARB1	scavenger receptor class B member 1	0.514	0.0044
GALNT15	polypeptide N-acetylgalactosaminyltransferase 15	0.997	0.0044
SLA	Src-like-adaptor	1.628	0.0044
HTRA4	HtrA serine peptidase 4	0.557	0.0044
NCALD	neurocalcin delta	-0.683	0.0044
GNAI2	G protein subunit alpha i2	0.643	0.0044
LINC01140	long intergenic non-protein coding RNA 1140	-1.877	0.0044
HLA-F	major histocompatibility complex, class I, F	0.739	0.0044
MYH11	myosin heavy chain 11	1.560	0.0045
SNORD116-3	small nucleolar RNA, C/D box 116-3	-1.402	0.0045
SNORD116-3	small nucleolar RNA, C/D box 116-3	-1.402	0.0045
CLIP4	CAP-Gly domain containing linker protein family member 4	1.142	0.0045
CASP10	caspase 10	0.639	0.0045
TPD52	tumor protein D52	-0.963	0.0045
CD52	CD52 molecule	1.977	0.0045
JARID2	jumonji and AT-rich interaction domain containing 2	0.739	0.0045
FCGR1CP	Fc fragment of IgG receptor Ic, pseudogene	1.961	0.0046
SYNJ2BP-COX16	SYNJ2BP-COX16 readthrough	-0.963	0.0046
NR3C1	nuclear receptor subfamily 3 group C member 1	0.770	0.0046
STEAP1	STEAP family member 1	-1.108	0.0046
LACTB2	lactamase beta 2	-0.983	0.0047
ARHGDIB	Rho GDP dissociation inhibitor beta	1.355	0.0047
SH3BP2	SH3 domain binding protein 2	0.563	0.0047
FKBP15	FK506 binding protein 15	0.869	0.0047

GADD45A	growth arrest and DNA damage inducible alpha	-0.951	0.0047
GNA15	G protein subunit alpha 15	1.468	0.0047
TATDN1	TatD DNase domain containing 1	-0.537	0.0047
MRPS15	mitochondrial ribosomal protein S15	-0.690	0.0047
HLA-F	major histocompatibility complex, class I, F	0.810	0.0047
CTDSPL	CTD small phosphatase like	-0.723	0.0047
RUBCN	RUN and cysteine rich domain containing beclin 1 interacting pro	0.862	0.0047
SKAP2	src kinase associated phosphoprotein 2	1.068	0.0047
FGR	FGR proto-oncogene, Src family tyrosine kinase	1.071	0.0047
TNNI2	troponin I2, fast skeletal type	0.619	0.0047
CASP1	caspase 1	1.361	0.0047
HTR1D	5-hydroxytryptamine receptor 1D	-0.794	0.0048
TWF1	twinfilin actin binding protein 1	-0.613	0.0048
HMOX1	heme oxygenase 1	1.962	0.0048
ZNF443	zinc finger protein 443	-0.939	0.0048
ZNF468	zinc finger protein 468	-0.939	0.0048
PDE4B	phosphodiesterase 4B	0.695	0.0048
BTG1	BTG anti-proliferation factor 1	0.857	0.0048
NDUFAF2	NADH:ubiquinone oxidoreductase complex assembly factor 2	-0.912	0.0048
EIF3J	eukaryotic translation initiation factor 3 subunit J	-0.564	0.0048
LTB	lymphotoxin beta	0.594	0.0048
LTB	lymphotoxin beta	0.594	0.0048
LTB	lymphotoxin beta	0.594	0.0048
MARCO	macrophage receptor with collagenous structure	1.957	0.0049
PLS1	plastin 1	-0.907	0.0049
LRPAP1	LDL receptor related protein associated protein 1	0.825	0.0049
CCL4L2	C-C motif chemokine ligand 4 like 2	1.142	0.0049
GAA	glucosidase alpha, acid	0.590	0.0049
PDE7B	phosphodiesterase 7B	1.022	0.0049
CNKSR3	CNKSR family member 3	-0.736	0.0049
FANCF	Fanconi anemia complementation group F	-0.485	0.0049
VNN2	vanin 2	2.349	0.0049
ARHGEF38	Rho guanine nucleotide exchange factor 38	-0.907	0.0050
CCDC69	coiled-coil domain containing 69	0.624	0.0050
RBPJ	recombination signal binding protein for immunoglobulin kappa .	0.811	0.0050
PLEK2	pleckstrin 2	0.558	0.0050
CD200	CD200 molecule	-1.089	0.0050
SLC36A1	solute carrier family 36 member 1	0.721	0.0050
TIMM9	translocase of inner mitochondrial membrane 9	-0.651	0.0050
ANKH	ANKH inorganic pyrophosphate transport regulator	0.686	0.0050
HLA-F	major histocompatibility complex, class I, F	0.676	0.0051
TNF	tumor necrosis factor	0.599	0.0051
TNF	tumor necrosis factor	0.599	0.0051
TNF	tumor necrosis factor	0.599	0.0051
APPBP2	amyloid beta precursor protein binding protein 2	-0.678	0.0051

LURAP1L	leucine rich adaptor protein 1 like	-0.833	0.0051
OLR1	oxidized low density lipoprotein receptor 1	1.616	0.0051
CPVL	carboxypeptidase, vitellogenic like	1.096	0.0051
TMPRSS4	transmembrane protease, serine 4	-1.052	0.0051
SH3BP5	SH3 domain binding protein 5	0.596	0.0051
UBD	ubiquitin D	0.539	0.0051
CASZ1	castor zinc finger 1	-0.613	0.0051
CHODL	chondrolectin	-0.924	0.0051
SLC2A5	solute carrier family 2 member 5	1.599	0.0052
ARHGAP15	Rho GTPase activating protein 15	1.337	0.0052
MTA3	metastasis associated 1 family member 3	-0.696	0.0052
HDGFRP3	hepatoma-derived growth factor, related protein 3	-0.715	0.0052
DOLPP1	dolichyldiphosphatase 1	-0.602	0.0053
TRPC6	transient receptor potential cation channel subfamily C member 6	-1.332	0.0053
IFT20	intraflagellar transport 20	-0.791	0.0053
PHF21A	PHD finger protein 21A	0.807	0.0053
CORO2A	coronin 2A	-0.651	0.0053
TOM1L1	target of myb1 like 1 membrane trafficking protein	-1.026	0.0053
LOC100294362	uncharacterized LOC100294362	0.441	0.0053
TFB2M	transcription factor B2, mitochondrial	-0.778	0.0053
LNPEP	leucyl and cystinyl aminopeptidase	0.503	0.0053
HLA-F	major histocompatibility complex, class I, F	0.695	0.0053
HLA-F	major histocompatibility complex, class I, F	0.695	0.0053
CD47	CD47 molecule	0.641	0.0053
MCOLN1	mucolipin 1	0.644	0.0053
CBY1	chibby family member 1, beta catenin antagonist	-0.462	0.0053
PLEKHO2	pleckstrin homology domain containing O2	0.549	0.0053
STX7	syntaxin 7	0.713	0.0054
VAV1	vav guanine nucleotide exchange factor 1	0.895	0.0054
SLC3A1	solute carrier family 3 member 1	-1.448	0.0054
PICALM	phosphatidylinositol binding clathrin assembly protein	0.549	0.0054
HCST	hematopoietic cell signal transducer	1.514	0.0054
P4HA3	prolyl 4-hydroxylase subunit alpha 3	0.557	0.0054
CEP295NL	CEP295 N-terminal like	0.887	0.0054
MKNK1	MAP kinase interacting serine/threonine kinase 1	0.791	0.0054
CSF1	colony stimulating factor 1	0.874	0.0054
ARHGAP5	Rho GTPase activating protein 5	-0.673	0.0054
CARD11	caspase recruitment domain family member 11	0.707	0.0055
WEE1	WEE1 G2 checkpoint kinase	-2.036	0.0055
TIAM1	T-cell lymphoma invasion and metastasis 1	0.675	0.0055
NLRP3	NLR family pyrin domain containing 3	0.978	0.0055
UAP1	UDP-N-acetylglucosamine pyrophosphorylase 1	-0.812	0.0055
TMED6	transmembrane p24 trafficking protein 6	-0.821	0.0055
CCL4L2	C-C motif chemokine ligand 4 like 2	1.276	0.0055
CCL4L2	C-C motif chemokine ligand 4 like 2	1.276	0.0055

SYTL2	synaptotagmin like 2	0.708	0.0055
TIMP1	TIMP metallopeptidase inhibitor 1	1.476	0.0055
SERPINB2	serpin family B member 2	1.426	0.0055
FAM78A	family with sequence similarity 78 member A	0.751	0.0055
HLA-F	major histocompatibility complex, class I, F	0.615	0.0055
HLA-F	major histocompatibility complex, class I, F	0.615	0.0055
RGS1	regulator of G-protein signaling 1	2.028	0.0055
TIAF1	TGFB1-induced anti-apoptotic factor 1	0.429	0.0055
SH2B3	SH2B adaptor protein 3	0.562	0.0055
SH3BGRL	SH3 domain binding glutamate rich protein like	0.719	0.0055
CCL5	C-C motif chemokine ligand 5	1.636	0.0055
GRB2	growth factor receptor bound protein 2	0.902	0.0055
PDCD5	programmed cell death 5	-0.583	0.0055
MDFIC	MyoD family inhibitor domain containing	0.592	0.0056
TREML1	triggering receptor expressed on myeloid cells like 1	0.891	0.0056
APITD1-CORT	APITD1-CORT readthrough	-0.505	0.0056
ESRP2	epithelial splicing regulatory protein 2	-0.533	0.0057
FCRL6	Fc receptor like 6	0.892	0.0057
ARL9	ADP ribosylation factor like GTPase 9	-0.763	0.0057
RALGDS	ral guanine nucleotide dissociation stimulator	0.722	0.0057
PAIP2B	poly(A) binding protein interacting protein 2B	-0.756	0.0057
IFNK	interferon kappa	0.746	0.0057
PTPRJ	protein tyrosine phosphatase, receptor type J	0.897	0.0057
PTAFR	platelet activating factor receptor	0.882	0.0057
ANKRD45	ankyrin repeat domain 45	-0.494	0.0057
ABI3BP	ABI family member 3 binding protein	2.025	0.0057
SLC50A1	solute carrier family 50 member 1	-0.784	0.0058
RASSF4	Ras association domain family member 4	0.601	0.0058
PIFO	primary cilia formation	-0.963	0.0058
CCT6A	chaperonin containing TCP1 subunit 6A	-0.601	0.0058
BRMS1L	breast cancer metastasis-suppressor 1-like	-0.608	0.0058
RORC	RAR related orphan receptor C	-0.615	0.0058
PIM2	Pim-2 proto-oncogene, serine/threonine kinase	0.820	0.0058
GINS3	GINS complex subunit 3	-0.847	0.0058
C11orf54	chromosome 11 open reading frame 54	-0.856	0.0058
NET1	neuroepithelial cell transforming 1	-0.920	0.0058
TOM1L1	target of myb1 like 1 membrane trafficking protein	-1.402	0.0059
JAK2	Janus kinase 2	0.684	0.0059
GRK5	G protein-coupled receptor kinase 5	0.769	0.0059
RNF182	ring finger protein 182	0.793	0.0059
CISD1	CDGSH iron sulfur domain 1	-0.735	0.0059
TLR7	toll like receptor 7	1.327	0.0059
MTF1	metal regulatory transcription factor 1	-1.100	0.0059
CD109	CD109 molecule	1.014	0.0059
DSE	dermatan sulfate epimerase	0.913	0.0059

MAN2B1	mannosidase alpha class 2B member 1	0.826	0.0060
PLCG2	phospholipase C gamma 2	0.787	0.0060
SIX4	SIX homeobox 4	-0.606	0.0060
HOXB6	homeobox B6	-1.015	0.0060
IFNGR2	interferon gamma receptor 2 (interferon gamma transducer 1)	0.780	0.0061
S100A1	S100 calcium binding protein A1	-0.894	0.0061
SLC30A1	solute carrier family 30 member 1	-0.632	0.0061
ATP2C1	ATPase secretory pathway Ca2+ transporting 1	-0.736	0.0061
SERPINA1	serpin family A member 1	1.834	0.0061
C5AR2	complement component 5a receptor 2	0.980	0.0061
FERMT3	fermitin family member 3	0.779	0.0061
SYTL5	synaptotagmin like 5	-0.975	0.0061
PFKFB2	6-phosphofructo-2-kinase/fructose-2,6-biphosphatase 2	-0.582	0.0062
ACPP	acid phosphatase, prostate	-1.329	0.0062
TEP1	telomerase associated protein 1	0.472	0.0062
KCTD12	potassium channel tetramerization domain containing 12	0.628	0.0062
SH3KBP1	SH3 domain containing kinase binding protein 1	0.856	0.0062
WDSUB1	WD repeat, sterile alpha motif and U-box domain containing 1	-0.597	0.0062
GOLPH3L	golgi phosphoprotein 3 like	-0.741	0.0062
MNDA	myeloid cell nuclear differentiation antigen	2.673	0.0062
LIPA	lipase A, lysosomal acid type	1.247	0.0063
HIST1H2BK	histone cluster 1 H2B family member k	-0.843	0.0063
CCR2	C-C motif chemokine receptor 2	1.074	0.0063
SNCA	synuclein alpha	2.374	0.0063
DENND3	DENN domain containing 3	0.980	0.0063
GPM6B	glycoprotein M6B	-0.559	0.0063
SLC26A2	solute carrier family 26 member 2	-1.781	0.0063
SORL1	sortilin related receptor 1	1.788	0.0064
HNF1A-AS1	HNF1A antisense RNA 1	-1.920	0.0064
S100A8	S100 calcium binding protein A8	3.361	0.0064
ABHD11	abhydrolase domain containing 11	-0.455	0.0064
A4GNT	alpha-1,4-N-acetylglucosaminyltransferase	-0.548	0.0064
PAG1	phosphoprotein membrane anchor with glycosphingolipid micro	1.098	0.0064
MXD1	MAX dimerization protein 1	1.411	0.0065
XCR1	X-C motif chemokine receptor 1	0.696	0.0065
SMYD3	SET and MYND domain containing 3	-0.558	0.0065
MYO9B	myosin IXB	0.666	0.0065
ETS1	ETS proto-oncogene 1, transcription factor	0.968	0.0065
SFT2D3	SFT2 domain containing 3	-0.434	0.0065
SLC31A2	solute carrier family 31 member 2	0.814	0.0065
CCL3L3	C-C motif chemokine ligand 3 like 3	1.087	0.0065
CCL3L3	C-C motif chemokine ligand 3 like 3	1.087	0.0065
CCL3L3	C-C motif chemokine ligand 3 like 3	1.087	0.0065
UNC5C	unc-5 netrin receptor C	-0.678	0.0065
FCRL1	Fc receptor like 1	0.982	0.0065

PLK3	polo like kinase 3	0.715	0.0065
SEC11C	SEC11 homolog C, signal peptidase complex subunit	-0.716	0.0065
HLA-F	major histocompatibility complex, class I, F	0.689	0.0065
CRLF1	cytokine receptor like factor 1	0.501	0.0065
DLAT	dihydrolipoamide S-acetyltransferase	-0.519	0.0065
DDX60	DEXD/H-box helicase 60	0.841	0.0065
TFEC	transcription factor EC	1.622	0.0066
CFH	complement factor H	2.942	0.0066
CD3D	CD3d molecule	1.585	0.0066
MTUS1	microtubule associated tumor suppressor 1	-1.046	0.0066
TNFSF15	tumor necrosis factor superfamily member 15	0.772	0.0067
NLRP1	NLR family pyrin domain containing 1	0.732	0.0067
ABR	active BCR-related	0.794	0.0067
C20orf85	chromosome 20 open reading frame 85	-1.331	0.0068
TET2	tet methylcytosine dioxygenase 2	0.750	0.0068
MORN2	MORN repeat containing 2	-1.005	0.0068
ZEB2	zinc finger E-box binding homeobox 2	1.046	0.0068
TMEM132B	transmembrane protein 132B	-0.593	0.0068
OSBPL8	oxysterol binding protein like 8	0.567	0.0068
MAP3K3	mitogen-activated protein kinase kinase kinase 3	0.422	0.0069
FST	follistatin	0.966	0.0069
GPR155	G protein-coupled receptor 155	0.987	0.0069
SLFN12L	schlafen family member 12 like	0.890	0.0069
UQCR10	ubiquinol-cytochrome c reductase, complex III subunit X	-0.601	0.0069
CD3G	CD3g molecule	1.614	0.0070
GPT2	glutamic--pyruvic transaminase 2	-0.823	0.0070
MAR3	membrane associated ring-CH-type finger 3	0.507	0.0070
ZNF492	zinc finger protein 492	-0.584	0.0070
CHRDL1	chordin like 1	1.469	0.0070
ERBB3	erb-b2 receptor tyrosine kinase 3	-1.005	0.0070
MME	membrane metalloendopeptidase	-1.508	0.0070
ZNF486	zinc finger protein 486	-1.223	0.0071
CEP170	centrosomal protein 170	0.677	0.0071
OVOS	ovostatin	-1.905	0.0071
TJP2	tight junction protein 2	-0.684	0.0071
KIAA1671	KIAA1671	-0.778	0.0072
CXCL5	C-X-C motif chemokine ligand 5	1.638	0.0072
EAF1	ELL associated factor 1	0.764	0.0072
ZNF385A	zinc finger protein 385A	0.900	0.0072
OVOS	ovostatin	-1.781	0.0072
CHST9	carbohydrate sulfotransferase 9	-1.026	0.0072
RUSC2	RUN and SH3 domain containing 2	0.434	0.0072
LPCAT3	lysophosphatidylcholine acyltransferase 3	-0.788	0.0072
SLAMF9	SLAM family member 9	1.227	0.0072
CAMK1D	calcium/calmodulin dependent protein kinase ID	0.937	0.0073

OCLN	occludin	-1.385	0.0073
EVL	Enah/Vasp-like	0.709	0.0073
TNFAIP2	TNF alpha induced protein 2	0.573	0.0073
ZNFX1	zinc finger NFX1-type containing 1	0.694	0.0073
SEMA4D	semaphorin 4D	1.017	0.0073
ACRBP	acrosin binding protein	0.404	0.0073
MYO1B	myosin IB	-1.003	0.0073
FOXA2	forkhead box A2	-0.478	0.0074
OR1Q1	olfactory receptor family 1 subfamily Q member 1	-1.172	0.0074
MCEMP1	mast cell expressed membrane protein 1	0.531	0.0074
TRIM38	tripartite motif containing 38	0.622	0.0074
RAPGEF1	Rap guanine nucleotide exchange factor 1	0.921	0.0075
DHRS7B	dehydrogenase/reductase 7B	-0.489	0.0075
CNN1	calponin 1	1.087	0.0075
ITK	IL2 inducible T-cell kinase	1.237	0.0075
RASGRP1	RAS guanyl releasing protein 1	1.120	0.0075
MGST1	microsomal glutathione S-transferase 1	-0.996	0.0075
GRB7	growth factor receptor bound protein 7	-0.731	0.0076
UBD	ubiquitin D	0.527	0.0076
DDT	D-dopachrome tautomerase	-0.486	0.0076
PHB	prohibitin	-0.636	0.0076
GABRA2	gamma-aminobutyric acid type A receptor alpha2 subunit	-1.036	0.0076
TMEM261	transmembrane protein 261	-0.734	0.0077
ARSD	arylsulfatase D	-0.560	0.0077
SNORD116-3	small nucleolar RNA, C/D box 116-3	-1.200	0.0077
GAB3	GRB2 associated binding protein 3	0.841	0.0077
KIAA0930	KIAA0930	0.586	0.0077
NIT2	nitrilase family member 2	-0.745	0.0078
TLR6	toll like receptor 6	1.212	0.0078
SSFA2	sperm specific antigen 2	-0.549	0.0078
AJUBA	ajuba LIM protein	0.491	0.0078
MMADHC	methylmalonic aciduria and homocystinuria, cblD type	-0.579	0.0078
GRAMD1A	GRAM domain containing 1A	0.419	0.0079
CACYBP	calcyclin binding protein	-0.859	0.0079
B2M	beta-2-microglobulin	0.526	0.0079
HLA-F	major histocompatibility complex, class I, F	0.735	0.0079
WISP1	WNT1 inducible signaling pathway protein 1	0.597	0.0079
NR2C1	nuclear receptor subfamily 2 group C member 1	-0.704	0.0079
B3GALNT1	beta-1,3-N-acetylgalactosaminyltransferase 1 (globoside blood gı	-0.702	0.0079
SPI1	Spi-1 proto-oncogene	0.913	0.0080
ERLEC1	endoplasmic reticulum lectin 1	-0.611	0.0080
CD200R1	CD200 receptor 1	1.116	0.0080
TLE1	transducin like enhancer of split 1	-1.376	0.0080
HLA-DQB2	major histocompatibility complex, class II, DQ beta 2	0.702	0.0080
HLA-F	major histocompatibility complex, class I, F	0.687	0.0081

HERC3	HECT and RLD domain containing E3 ubiquitin protein ligase 3	0.822	0.0081
MSH2	mutS homolog 2	-0.670	0.0081
COTL1	coactosin like F-actin binding protein 1	1.068	0.0082
CLUH	clustered mitochondria homolog	-0.428	0.0082
TBC1D30	TBC1 domain family member 30	-0.575	0.0082
GPX2	glutathione peroxidase 2	-0.896	0.0082
OR1N1	olfactory receptor family 1 subfamily N member 1	-1.391	0.0082
TLE3	transducin like enhancer of split 3	0.732	0.0082
CMTM8	CKLF like MARVEL transmembrane domain containing 8	-0.707	0.0082
MED19	mediator complex subunit 19	-0.418	0.0082
SNORA28	small nucleolar RNA, H/ACA box 28	-1.643	0.0082
ANKRD13A	ankyrin repeat domain 13A	0.787	0.0082
FBXO16	F-box protein 16	-0.754	0.0082
ZNF562	zinc finger protein 562	-0.525	0.0083
CAMK2G	calcium/calmodulin dependent protein kinase II gamma	0.493	0.0083
LINC00471	long intergenic non-protein coding RNA 471	-0.539	0.0083
NYNRIN	NYN domain and retroviral integrase containing	0.534	0.0084
PSTPIP2	proline-serine-threonine phosphatase interacting protein 2	0.954	0.0084
ZBTB10	zinc finger and BTB domain containing 10	-0.543	0.0084
ADM	adrenomedullin	0.844	0.0084
OTUD1	OTU deubiquitinase 1	0.863	0.0084
KDELR3	KDEL endoplasmic reticulum protein retention receptor 3	-1.010	0.0084
COX6A1	cytochrome c oxidase subunit 6A1	-0.529	0.0085
HEXB	hexosaminidase subunit beta	0.792	0.0085
HAT1	histone acetyltransferase 1	-0.696	0.0086
ALPK2	alpha kinase 2	1.203	0.0087
SLC16A6	solute carrier family 16 member 6	0.829	0.0088
AMPD3	adenosine monophosphate deaminase 3	0.787	0.0088
CECR1	cat eye syndrome chromosome region, candidate 1	0.770	0.0088
SNX4	sorting nexin 4	-0.643	0.0088
KCNT2	potassium sodium-activated channel subfamily T member 2	0.772	0.0088
STRAP	serine/threonine kinase receptor associated protein	-0.550	0.0088
GRHL2	grainyhead like transcription factor 2	-0.967	0.0088
PNPLA8	patatin like phospholipase domain containing 8	-0.736	0.0088
C19orf66	chromosome 19 open reading frame 66	0.566	0.0088
DSP	desmoplakin	-1.296	0.0088
ERBB2	erb-b2 receptor tyrosine kinase 2	-0.561	0.0088
NDUFAF4	NADH:ubiquinone oxidoreductase complex assembly factor 4	-0.809	0.0089
MRPS36	mitochondrial ribosomal protein S36	-1.176	0.0089
SSH1	slingshot protein phosphatase 1	0.455	0.0089
DMXL2	Dmx like 2	1.253	0.0089
UGDH	UDP-glucose 6-dehydrogenase	-1.045	0.0089
RNF38	ring finger protein 38	0.657	0.0090
TNFRSF10D	TNF receptor superfamily member 10d	0.614	0.0090
ADH6	alcohol dehydrogenase 6 (class V)	-0.702	0.0090

LAMP3	lysosomal associated membrane protein 3	0.742	0.0090
PIK3C2G	phosphatidylinositol-4-phosphate 3-kinase catalytic subunit type	-0.806	0.0090
GIMAP8	GTPase, IMAP family member 8	0.746	0.0091
AK7	adenylate kinase 7	-0.708	0.0092
LRRC1	leucine rich repeat containing 1	-1.707	0.0092
IL1RN	interleukin 1 receptor antagonist	1.471	0.0092
CCDC58	coiled-coil domain containing 58	-0.631	0.0092
PTPN3	protein tyrosine phosphatase, non-receptor type 3	-1.148	0.0092
NOP16	NOP16 nucleolar protein	-0.670	0.0092
GALK2	galactokinase 2	-0.533	0.0092
UCHL1	ubiquitin C-terminal hydrolase L1	0.961	0.0092
ARL11	ADP ribosylation factor like GTPase 11	0.628	0.0092
KLHDC2	kelch domain containing 2	-0.799	0.0092
RAB25	RAB25, member RAS oncogene family	-0.984	0.0092
TRAPPC4	trafficking protein particle complex 4	-0.599	0.0093
HMGCR	3-hydroxy-3-methylglutaryl-CoA reductase	-1.201	0.0093
CCL4	C-C motif chemokine ligand 4	1.035	0.0093
RANBP2	RAN binding protein 2	-0.788	0.0094
EPHX1	epoxide hydrolase 1	-0.756	0.0094
ABCA10	ATP binding cassette subfamily A member 10	-0.960	0.0095
TNFSF13B	tumor necrosis factor superfamily member 13b	1.306	0.0095
ZBED2	zinc finger BED-type containing 2	0.609	0.0095
HLA-DQB2	major histocompatibility complex, class II, DQ beta 2	0.690	0.0095
RAB40B	RAB40B, member RAS oncogene family	-0.624	0.0095
PLCB1	phospholipase C beta 1	-1.138	0.0095
SERINC2	serine incorporator 2	-0.836	0.0095
AGR2	anterior gradient 2, protein disulphide isomerase family member	-1.745	0.0095
CD3E	CD3e molecule	1.142	0.0096
SRI	sorcin	-0.764	0.0096
ANXA2	annexin A2	-0.719	0.0096
AGER	advanced glycosylation end-product specific receptor	0.441	0.0096
LRMP	lymphoid restricted membrane protein	1.438	0.0096
SH3YL1	SH3 and SYLF domain containing 1	-0.681	0.0096
LYST	lysosomal trafficking regulator	0.890	0.0096
IFNAR2	interferon alpha and beta receptor subunit 2	0.655	0.0096
LTBP1	latent transforming growth factor beta binding protein 1	1.235	0.0096
TMEM39A	transmembrane protein 39A	-0.504	0.0096
TOMM70	translocase of outer mitochondrial membrane 70	-0.591	0.0096
CASZ1	castor zinc finger 1	-0.799	0.0096
OSBPL11	oxysterol binding protein like 11	0.530	0.0096
PRG2	proteoglycan 2, pro eosinophil major basic protein	0.586	0.0096
FLNA	filamin A	0.886	0.0097
GJA5	gap junction protein alpha 5	-0.406	0.0098
GCLM	glutamate-cysteine ligase modifier subunit	-0.562	0.0098
FYN	FYN proto-oncogene, Src family tyrosine kinase	0.799	0.0098

HERC6	HECT and RLD domain containing E3 ubiquitin protein ligase fan	0.798	0.0098
GUCY1B3	guanylate cyclase 1 soluble subunit beta	-0.820	0.0099
ADGRL4	adhesion G protein-coupled receptor L4	-1.622	0.0099
PPA2	pyrophosphatase (inorganic) 2	-0.513	0.0099
CAPSL	calcyphosine like	-0.859	0.0099
GPRIN3	GPRIN family member 3	0.611	0.0099
MRPS33	mitochondrial ribosomal protein S33	-0.999	0.0099
PPP2R3C	protein phosphatase 2 regulatory subunit B''gamma	-0.535	0.0099
C11orf63	chromosome 11 open reading frame 63	-0.778	0.0100
TMED3	transmembrane p24 trafficking protein 3	-0.583	0.0100
GIPC2	GIPC PDZ domain containing family member 2	-0.663	0.0100
HOXB-AS3	HOXB cluster antisense RNA 3	-0.678	0.0100
LINC00649	long intergenic non-protein coding RNA 649	0.567	0.0100
TEX101	testis expressed 101	-0.622	0.0101
CCL18	C-C motif chemokine ligand 18	1.468	0.0101
PLEKHA5	pleckstrin homology domain containing A5	-1.414	0.0101
BTBD6	BTB domain containing 6	-0.590	0.0101
TWF1	twinfilin actin binding protein 1	-0.694	0.0101
RASA2	RAS p21 protein activator 2	0.500	0.0101
CD2	CD2 molecule	1.171	0.0101
EFCAB2	EF-hand calcium binding domain 2	-0.969	0.0101
VBP1	VHL binding protein 1	-0.918	0.0102
LIX1	limb and CNS expressed 1	1.111	0.0102
CCL8	C-C motif chemokine ligand 8	1.064	0.0102
PRKG1	protein kinase, cGMP-dependent, type I	-0.630	0.0103
APOL3	apolipoprotein L3	0.338	0.0103
C10orf88	chromosome 10 open reading frame 88	-0.552	0.0104
ZDHHC23	zinc finger DHHC-type containing 23	-0.371	0.0104
NDUFAF5	NADH:ubiquinone oxidoreductase complex assembly factor 5	-0.427	0.0104
ZNF555	zinc finger protein 555	-0.547	0.0104
DENND4B	DENN domain containing 4B	0.736	0.0105
PARD3	par-3 family cell polarity regulator	-0.918	0.0105
ADGRG3	adhesion G protein-coupled receptor G3	0.893	0.0105
PYHIN1	pyrin and HIN domain family member 1	1.131	0.0105
TSC22D3	TSC22 domain family member 3	0.702	0.0105
ANKRD1	ankyrin repeat domain 1	0.697	0.0105
LZTFL1	leucine zipper transcription factor like 1	-0.592	0.0105
TREM2	triggering receptor expressed on myeloid cells 2	1.010	0.0106
ZNF702P	zinc finger protein 702, pseudogene	-0.666	0.0106
ZNF432	zinc finger protein 432	-0.763	0.0106
MYO6	myosin VI	-1.336	0.0106
EPC1	enhancer of polycomb homolog 1	1.591	0.0107
ANXA4	annexin A4	-1.141	0.0107
SNORD116-1	small nucleolar RNA, C/D box 116-1	-0.948	0.0107
BACH1	BTB domain and CNC homolog 1	0.665	0.0108

PATJ	PATJ, crumbs cell polarity complex component	-0.953	0.0108
ACP5	acid phosphatase 5, tartrate resistant	0.951	0.0109
TTLL12	tubulin tyrosine ligase like 12	-0.417	0.0109
C3orf30	chromosome 3 open reading frame 30	-1.034	0.0109
NT5C1B-RDH14	NT5C1B-RDH14 readthrough	-0.501	0.0109
LRRFIP1	LRR binding FLII interacting protein 1	0.901	0.0109
NAE1	NEDD8 activating enzyme E1 subunit 1	-0.727	0.0110
ZNF644	zinc finger protein 644	-0.432	0.0110
SCGB1D1	secretoglobin family 1D member 1	-0.503	0.0110
PGA3	pepsinogen 3, group I (pepsinogen A)	0.692	0.0110
TRMT10A	tRNA methyltransferase 10A	-0.749	0.0110
SH2D4A	SH2 domain containing 4A	-0.912	0.0111
C11orf74	chromosome 11 open reading frame 74	-0.796	0.0111
CCDC169	coiled-coil domain containing 169	-0.465	0.0112
MDH1B	malate dehydrogenase 1B	-0.665	0.0112
F2RL1	F2R like trypsin receptor 1	-0.510	0.0112
TNFAIP8	TNF alpha induced protein 8	1.085	0.0112
CEP57L1	centrosomal protein 57 like 1	-0.636	0.0112
TAGLN	transgelin	1.054	0.0112
LILRB4	leukocyte immunoglobulin like receptor B4	1.212	0.0113
PAIP1	poly(A) binding protein interacting protein 1	-0.541	0.0113
USP32P2	ubiquitin specific peptidase 32 pseudogene 2	0.442	0.0113
SPOCK2	SPARC/osteonectin, cwcv and kazal like domains proteoglycan 2	0.526	0.0113
TXN	thioredoxin	-0.665	0.0114
SIPA1L1	signal induced proliferation associated 1 like 1	0.870	0.0114
PIK3R6	phosphoinositide-3-kinase regulatory subunit 6	0.794	0.0114
BANK1	B-cell scaffold protein with ankyrin repeats 1	0.990	0.0114
BIRC3	baculoviral IAP repeat containing 3	1.013	0.0114
UGGT2	UDP-glucose glycoprotein glucosyltransferase 2	-0.903	0.0114
ADARB1	adenosine deaminase, RNA specific B1	0.689	0.0114
TRIM14	tripartite motif containing 14	0.650	0.0114
FGF7P6	fibroblast growth factor 7 pseudogene 6	-0.537	0.0115
STAU2	staufen double-stranded RNA binding protein 2	-0.524	0.0115
CNPY3	canopy FGF signaling regulator 3	0.547	0.0115
LYSMD3	LysM domain containing 3	-0.687	0.0115
RSRP1	arginine and serine rich protein 1	-0.494	0.0115
GABRP	gamma-aminobutyric acid type A receptor pi subunit	-1.426	0.0115
HAL	histidine ammonia-lyase	-1.066	0.0115
ATP8B4	ATPase phospholipid transporting 8B4 (putative)	1.220	0.0115
NAA50	N(alpha)-acetyltransferase 50, NatE catalytic subunit	-0.689	0.0115
CFAP53	cilia and flagella associated protein 53	-0.978	0.0115
HDHD3	haloacid dehalogenase like hydrolase domain containing 3	-0.510	0.0115
MAPRE2	microtubule associated protein RP/EB family member 2	0.582	0.0115
FBXO4	F-box protein 4	-0.745	0.0115
ADGRB3	adhesion G protein-coupled receptor B3	-0.736	0.0115

MTERF2	mitochondrial transcription termination factor 2	-0.617	0.0115
DPY30	dpy-30, histone methyltransferase complex regulatory subunit	-0.980	0.0116
SNORD116-15	small nucleolar RNA, C/D box 116-15	-1.017	0.0116
AGER	advanced glycosylation end-product specific receptor	0.382	0.0116
CFLAR	CASP8 and FADD like apoptosis regulator	0.596	0.0116
HPCAL1	hippocalcin like 1	0.398	0.0117
EPC1	enhancer of polycomb homolog 1	0.966	0.0117
ALG2	ALG2, alpha-1,3/1,6-mannosyltransferase	-0.496	0.0117
COL4A4	collagen type IV alpha 4 chain	0.721	0.0117
WDR78	WD repeat domain 78	-0.802	0.0117
SMAP2	small ArfGAP2	1.287	0.0117
GGCT	gamma-glutamylcyclotransferase	-0.625	0.0117
SPACA6	sperm acrosome associated 6	0.447	0.0117
EGR2	early growth response 2	1.076	0.0117
EPCAM	epithelial cell adhesion molecule	-1.068	0.0117
FLRT2	fibronectin leucine rich transmembrane protein 2	-0.998	0.0117
CLDN20	claudin 20	0.542	0.0118
GCC2	GRIP and coiled-coil domain containing 2	-0.592	0.0118
AGER	advanced glycosylation end-product specific receptor	0.440	0.0118
IGF1	insulin like growth factor 1	-1.885	0.0118
SF3B6	splicing factor 3b subunit 6	-0.870	0.0119
AP5M1	adaptor related protein complex 5 mu 1 subunit	-0.515	0.0120
INPP4A	inositol polyphosphate-4-phosphatase type I A	0.688	0.0120
HLA-F	major histocompatibility complex, class I, F	0.610	0.0121
RNU6ATAC	RNA, U6atac small nuclear (U12-dependent splicing)	-0.750	0.0122
PDIA5	protein disulfide isomerase family A member 5	-0.876	0.0122
APOBR	apolipoprotein B receptor	0.665	0.0122
RANBP17	RAN binding protein 17	-1.505	0.0122
ANAPC11	anaphase promoting complex subunit 11	-0.671	0.0122
ROPN1L	rhophilin associated tail protein 1 like	-0.821	0.0122
EBAG9	estrogen receptor binding site associated, antigen, 9	-0.455	0.0122
LIPC	lipase C, hepatic type	-0.691	0.0122
CLDN3	claudin 3	-0.741	0.0122
CAMKMT	calmodulin-lysine N-methyltransferase	-0.599	0.0122
GZMK	granzyme K	1.565	0.0122
PIP4K2A	phosphatidylinositol-5-phosphate 4-kinase type 2 alpha	1.116	0.0122
KLHL5	kelch like family member 5	0.491	0.0123
FANCE	Fanconi anemia complementation group E	0.511	0.0123
CYLD	CYLD lysine 63 deubiquitinase	0.619	0.0123
MTX2	metaxin 2	-0.812	0.0123
LINC01356	long intergenic non-protein coding RNA 1356	-0.593	0.0124
SUMO1	small ubiquitin-like modifier 1	-0.685	0.0125
AADACL2	arylacetamide deacetylase like 2	-0.721	0.0125
LGR4	leucine rich repeat containing G protein-coupled receptor 4	-1.213	0.0126
PDCL	phosducin like	-0.648	0.0126

DGKA	diacylglycerol kinase alpha	0.821	0.0127
PYCARD	PYD and CARD domain containing	0.819	0.0127
FGF7	fibroblast growth factor 7	-1.656	0.0127
CCDC113	coiled-coil domain containing 113	-0.816	0.0127
TBXAS1	thromboxane A synthase 1	0.961	0.0127
LMO3	LIM domain only 3	1.109	0.0127
PDK1	pyruvate dehydrogenase kinase 1	0.731	0.0128
PMM2	phosphomannomutase 2	-0.649	0.0128
DSCC1	DNA replication and sister chromatid cohesion 1	-0.835	0.0128
SIGLEC5	sialic acid binding Ig like lectin 5	0.854	0.0128
NUCB2	nucleobindin 2	-0.784	0.0128
HLA-F	major histocompatibility complex, class I, F	0.709	0.0128
DESI1	desumoylating isopeptidase 1	-0.541	0.0130
TRMT5	tRNA methyltransferase 5	-0.482	0.0130
SFXN3	sideroflexin 3	0.495	0.0131
C12orf57	chromosome 12 open reading frame 57	-0.618	0.0131
PLEKHA2	pleckstrin homology domain containing A2	0.763	0.0132
KIF21A	kinesin family member 21A	-0.814	0.0132
LRRFIP1	LRR binding FLII interacting protein 1	1.419	0.0132
SPAG16	sperm associated antigen 16	-0.600	0.0133
EPCAM	epithelial cell adhesion molecule	-1.128	0.0134
PPP1R15A	protein phosphatase 1 regulatory subunit 15A	0.726	0.0134
SNORD60	small nucleolar RNA, C/D box 60	-0.512	0.0134
TMTC2	transmembrane and tetratricopeptide repeat containing 2	-0.631	0.0135
YES1	YES proto-oncogene 1, Src family tyrosine kinase	-0.765	0.0135
RAB39A	RAB39A, member RAS oncogene family	0.732	0.0135
FGD3	FYVE, RhoGEF and PH domain containing 3	0.789	0.0135
TNFSF4	tumor necrosis factor superfamily member 4	0.645	0.0135
AAK1	AP2 associated kinase 1	0.591	0.0136
RAB2A	RAB2A, member RAS oncogene family	-0.409	0.0136
PPP1R3C	protein phosphatase 1 regulatory subunit 3C	0.530	0.0137
ZNF816	zinc finger protein 816	-0.569	0.0138
C1orf194	chromosome 1 open reading frame 194	-1.477	0.0138
NOV	nephroblastoma overexpressed	-0.799	0.0138
ZNF823	zinc finger protein 823	-0.673	0.0139
REN	renin	1.602	0.0140
MGST2	microsomal glutathione S-transferase 2	-0.861	0.0141
EIF4E3	eukaryotic translation initiation factor 4E family member 3	-0.834	0.0141
IER3IP1	immediate early response 3 interacting protein 1	-0.543	0.0141
MRPL36	mitochondrial ribosomal protein L36	-0.686	0.0141
FAM60A	family with sequence similarity 60 member A	-0.935	0.0141
LRRC15	leucine rich repeat containing 15	0.515	0.0142
MFSD1	major facilitator superfamily domain containing 1	0.731	0.0143
C6	complement C6	-0.760	0.0143
HDDC2	HD domain containing 2	-0.617	0.0143

ZNF438	zinc finger protein 438	0.750	0.0143
ZEB2	zinc finger E-box binding homeobox 2	1.359	0.0143
ARHGAP18	Rho GTPase activating protein 18	0.961	0.0143
THEMIS	thymocyte selection associated	1.244	0.0143
DDB2	damage specific DNA binding protein 2	0.398	0.0143
F5	coagulation factor V	0.789	0.0144
COX5A	cytochrome c oxidase subunit 5A	-0.555	0.0144
MMP10	matrix metallopeptidase 10	1.627	0.0144
ZCWPW2	zinc finger CW-type and PWWP domain containing 2	1.116	0.0144
HAMP	hepcidin antimicrobial peptide	1.026	0.0144
EMB	embigin	0.975	0.0144
ATMIN	ATM interactor	-0.437	0.0144
SGK1	serum/glucocorticoid regulated kinase 1	-1.183	0.0144
NDUFAF2	NADH:ubiquinone oxidoreductase complex assembly factor 2	-0.593	0.0144
SLIRP	SRA stem-loop interacting RNA binding protein	-0.654	0.0144
ITGB5	integrin subunit beta 5	0.528	0.0144
IGF2BP3	insulin like growth factor 2 mRNA binding protein 3	0.574	0.0144
LOC645166	lymphocyte-specific protein 1 pseudogene	0.793	0.0144
TADA2A	transcriptional adaptor 2A	-0.434	0.0145
FAM47E-STBD1	FAM47E-STBD1 readthrough	-0.522	0.0145
GNB5	G protein subunit beta 5	-0.436	0.0145
CNTRL	centriolin	0.722	0.0146
CNOT11	CCR4-NOT transcription complex subunit 11	-0.511	0.0146
CD80	CD80 molecule	0.885	0.0146
HIST1H4C	histone cluster 1 H4 family member c	-1.117	0.0146
MIR29B2	microRNA 29b-2	0.608	0.0146
ARFGAP3	ADP ribosylation factor GTPase activating protein 3	-0.467	0.0146
TARP	TCR gamma alternate reading frame protein	1.256	0.0146
FAM46C	family with sequence similarity 46 member C	1.343	0.0146
HHEX	hematopoietically expressed homeobox	0.656	0.0146
SLC30A9	solute carrier family 30 member 9	-0.625	0.0147
GPN3	GPN-loop GTPase 3	-0.764	0.0147
SNRPE	small nuclear ribonucleoprotein polypeptide E	-0.641	0.0147
HMOX2	heme oxygenase 2	-0.478	0.0148
NCF1B	neutrophil cytosolic factor 1B pseudogene	1.523	0.0149
AGPAT4-IT1	AGPAT4 intronic transcript 1	0.638	0.0149
C16orf54	chromosome 16 open reading frame 54	0.561	0.0149
C16orf54	chromosome 16 open reading frame 54	0.561	0.0149
COL28A1	collagen type XXVIII alpha 1 chain	-0.657	0.0149
COX11	COX11, cytochrome c oxidase copper chaperone	-0.682	0.0149
IL2RA	interleukin 2 receptor subunit alpha	0.815	0.0149
RASSF2	Ras association domain family member 2	1.092	0.0150
SNORD116-3	small nucleolar RNA, C/D box 116-3	-1.103	0.0150
SNORD116-3	small nucleolar RNA, C/D box 116-3	-1.103	0.0150
TMEM165	transmembrane protein 165	-0.612	0.0150

GPC4	glypican 4	-0.804	0.0150
NCF1	neutrophil cytosolic factor 1	1.432	0.0150
CST7	cystatin F	0.937	0.0150
C8orf34	chromosome 8 open reading frame 34	-0.570	0.0150
ILF2	interleukin enhancer binding factor 2	-0.744	0.0151
MIPOL1	mirror-image polydactyly 1	-1.092	0.0151
GPR65	G protein-coupled receptor 65	1.363	0.0151
IKBKE	inhibitor of kappa light polypeptide gene enhancer in B-cells, kina	0.529	0.0151
ZYX	zyxin	0.619	0.0151
CAPZB	capping actin protein of muscle Z-line beta subunit	0.486	0.0151
POLR1D	RNA polymerase I subunit D	-0.372	0.0151
FAM126A	family with sequence similarity 126 member A	0.575	0.0151
FERMT1	fermitin family member 1	-0.688	0.0152
ERV3-1	endogenous retrovirus group 3 member 1	0.548	0.0152
MTHFS	5,10-methenyltetrahydrofolate synthetase (5-formyltetrahydrofola	-0.751	0.0152
LRRN4	leucine rich repeat neuronal 4	0.554	0.0152
CD274	CD274 molecule	0.961	0.0152
EMP1	epithelial membrane protein 1	0.917	0.0153
AFDN-AS1	AFDN antisense RNA 1 (head to head)	-0.535	0.0154
PCNP	PEST proteolytic signal containing nuclear protein	-0.575	0.0154
FAM65A	family with sequence similarity 65 member A	0.383	0.0154
HINT1	histidine triad nucleotide binding protein 1	-0.404	0.0155
TRMT10C	tRNA methyltransferase 10C, mitochondrial RNase P subunit	-0.725	0.0156
SAMD8	sterile alpha motif domain containing 8	0.596	0.0156
PPA1	pyrophosphatase (inorganic) 1	-0.825	0.0156
NCF1	neutrophil cytosolic factor 1	1.504	0.0158
RGMB	repulsive guidance molecule family member b	-0.505	0.0158
PIR	pirin	-0.745	0.0158
ANKRD55	ankyrin repeat domain 55	-1.170	0.0158
ZCRB1	zinc finger CCHC-type and RNA binding motif containing 1	-0.546	0.0158
TNIK	TRAF2 and NCK interacting kinase	0.950	0.0159
PFDN6	prefoldin subunit 6	-0.720	0.0159
PFDN6	prefoldin subunit 6	-0.720	0.0159
PFDN6	prefoldin subunit 6	-0.720	0.0159
ZCWPW2	zinc finger CW-type and PWWP domain containing 2	0.623	0.0159
C2orf47	chromosome 2 open reading frame 47	-0.676	0.0162
SCIN	scinderin	0.734	0.0162
DPYSL3	dihydropyrimidinase like 3	0.608	0.0162
ADGRE3	adhesion G protein-coupled receptor E3	1.639	0.0162
RASD1	ras related dexamethasone induced 1	-0.643	0.0163
FURIN	furin, paired basic amino acid cleaving enzyme	0.457	0.0163
NBEAL2	neurobeachin like 2	0.456	0.0163
SPA17	sperm autoantigenic protein 17	-1.035	0.0163
RBM7	RNA binding motif protein 7	-0.778	0.0163
PDCD1LG2	programmed cell death 1 ligand 2	0.961	0.0163

CCDC88A	coiled-coil domain containing 88A	0.800	0.0164
BMP2K	BMP2 inducible kinase	0.739	0.0164
DENR	density regulated re-initiation and release factor	-0.552	0.0164
GAD1	glutamate decarboxylase 1	-0.425	0.0164
DENND2D	DENN domain containing 2D	0.783	0.0165
BST2	bone marrow stromal cell antigen 2	0.797	0.0165
HSPA6	heat shock protein family A (Hsp70) member 6	1.192	0.0165
SUSD6	sushi domain containing 6	0.513	0.0165
MCCC2	methylcrotonoyl-CoA carboxylase 2	-1.047	0.0165
MCCC2	methylcrotonoyl-CoA carboxylase 2	-1.047	0.0165
PI4K2B	phosphatidylinositol 4-kinase type 2 beta	-0.548	0.0165
MRPS14	mitochondrial ribosomal protein S14	-0.704	0.0165
ADSS	adenylosuccinate synthase	-0.541	0.0165
SYAP1	synapse associated protein 1	-0.444	0.0165
EHF	ETS homologous factor	-1.492	0.0165
EBP	emopamil binding protein (sterol isomerase)	-0.575	0.0166
SCCPDH	saccharopine dehydrogenase (putative)	-0.675	0.0167
PPP2CB	protein phosphatase 2 catalytic subunit beta	-0.659	0.0167
CXCR1	C-X-C motif chemokine receptor 1	2.146	0.0167
MCEE	methylmalonyl-CoA epimerase	-0.727	0.0168
GPSM3	G-protein signaling modulator 3	0.821	0.0169
SLCO3A1	solute carrier organic anion transporter family member 3A1	0.538	0.0169
MAP7	microtubule associated protein 7	-0.859	0.0169
ABI3	ABI family member 3	0.661	0.0169
RAMP2	receptor activity modifying protein 2	-0.915	0.0169
OR51E1	olfactory receptor family 51 subfamily E member 1	-0.476	0.0169
LBH	limb bud and heart development	0.764	0.0170
DRAM1	DNA damage regulated autophagy modulator 1	0.612	0.0170
PDE7A	phosphodiesterase 7A	0.626	0.0170
NUAK2	NUAK family kinase 2	0.690	0.0171
SLED1	proteoglycan 3 pseudogene	0.492	0.0172
ALG14	ALG14, UDP-N-acetylglucosaminyltransferase subunit	-0.807	0.0172
ZNF280C	zinc finger protein 280C	-0.664	0.0172
EIF5AL1	eukaryotic translation initiation factor 5A-like 1	-0.686	0.0173
CXCL8	C-X-C motif chemokine ligand 8	2.315	0.0173
NDUFA1	NADH:ubiquinone oxidoreductase subunit A1	-0.434	0.0174
ENAH	enabled homolog (Drosophila)	-0.896	0.0174
PIGZ	phosphatidylinositol glycan anchor biosynthesis class Z	-0.345	0.0175
RPP40	ribonuclease P/MRP subunit p40	-0.604	0.0175
SPRED1	sprouty related EVH1 domain containing 1	0.558	0.0175
UNC119	unc-119 lipid binding chaperone	0.615	0.0176
EHBP1L1	EH domain binding protein 1 like 1	0.334	0.0176
TTC13	tetratricopeptide repeat domain 13	0.399	0.0176
COX7B	cytochrome c oxidase subunit 7B	-0.914	0.0176
SAMSN1	SAM domain, SH3 domain and nuclear localization signals 1	1.415	0.0176

NDUFA11	NADH:ubiquinone oxidoreductase subunit A11	-0.404	0.0176
LMBR1L	limb development membrane protein 1 like	0.499	0.0177
PTCH1	patched 1	-0.785	0.0177
PKM	pyruvate kinase, muscle	0.832	0.0177
LY86	lymphocyte antigen 86	1.288	0.0177
DIMT1	DIM1 dimethyladenosine transferase 1 homolog	-0.462	0.0177
TSPAN1	tetraspanin 1	-1.127	0.0178
CHMP4C	charged multivesicular body protein 4C	-0.916	0.0179
LY6E	lymphocyte antigen 6 complex, locus E	0.586	0.0179
CCNC	cyclin C	-0.639	0.0179
KCNJ16	potassium voltage-gated channel subfamily J member 16	1.076	0.0179
IQSEC1	IQ motif and Sec7 domain 1	0.768	0.0180
NEBL	nebulette	-0.987	0.0180
FPR2	formyl peptide receptor 2	1.028	0.0180
COX7A2	cytochrome c oxidase subunit 7A2	-0.803	0.0180
ZNF525	zinc finger protein 525	-0.350	0.0181
S1PR1	sphingosine-1-phosphate receptor 1	0.809	0.0181
CSRNP3	cysteine and serine rich nuclear protein 3	-0.559	0.0181
PKP2	plakophilin 2	-1.060	0.0181
PXMP2	peroxisomal membrane protein 2	-0.619	0.0181
PTPRK	protein tyrosine phosphatase, receptor type K	-1.059	0.0183
ANKRD20A3	ankyrin repeat domain 20 family member A3	-0.634	0.0183
GLIPR2	GLI pathogenesis related 2	0.747	0.0184
ATP2A2	ATPase sarcoplasmic/endoplasmic reticulum Ca2+ transporting 2	-0.896	0.0184
MOB3A	MOB kinase activator 3A	0.627	0.0184
GTF2H5	general transcription factor IIH subunit 5	-0.507	0.0185
PIP4K2C	phosphatidylinositol-5-phosphate 4-kinase type 2 gamma	-0.520	0.0185
HLA-F	major histocompatibility complex, class I, F	0.658	0.0185
IRF9	interferon regulatory factor 9	0.559	0.0186
LNPK	lunapark, ER junction formation factor	0.577	0.0186
AKAP7	A-kinase anchoring protein 7	-0.632	0.0186
DTWD1	DTW domain containing 1	-0.890	0.0186
SMCO4	single-pass membrane protein with coiled-coil domains 4	0.625	0.0186
HPGD	hydroxyprostaglandin dehydrogenase 15-(NAD)	-1.343	0.0186
TBCA	tubulin folding cofactor A	-0.455	0.0186
PPP1R36	protein phosphatase 1 regulatory subunit 36	-0.618	0.0186
TSPAN18	tetraspanin 18	0.665	0.0187
CD5	CD5 molecule	0.661	0.0187
PTGDS	prostaglandin D2 synthase	-0.557	0.0187
SLC2A1	solute carrier family 2 member 1	0.896	0.0188
PCNA	proliferating cell nuclear antigen	-0.424	0.0188
ZNF670	zinc finger protein 670	-0.799	0.0188
PDGFD	platelet derived growth factor D	1.455	0.0188
CRTAM	cytotoxic and regulatory T-cell molecule	0.883	0.0188
PTGIS	prostaglandin I2 synthase	0.790	0.0189

PGD	phosphogluconate dehydrogenase	0.904	0.0189
HSPB11	heat shock protein family B (small) member 11	-0.749	0.0189
ENC1	ectodermal-neural cortex 1	0.466	0.0190
ETAA1	Ewing tumor associated antigen 1	-0.790	0.0190
ZNF639	zinc finger protein 639	-0.552	0.0190
PCLO	piccolo presynaptic cytomatrix protein	-0.581	0.0190
CD300C	CD300c molecule	0.471	0.0190
TFPI	tissue factor pathway inhibitor	0.984	0.0191
ACSL3	acyl-CoA synthetase long-chain family member 3	-0.573	0.0191
NAMPT	nicotinamide phosphoribosyltransferase	0.815	0.0191
TM4SF18	transmembrane 4 L six family member 18	-1.009	0.0191
PXYLP1	2-phosphoxylose phosphatase 1	0.857	0.0191
PODNL1	podocan like 1	0.476	0.0191
CH25H	cholesterol 25-hydroxylase	1.544	0.0191
SIRPG	signal regulatory protein gamma	0.703	0.0191
ATL3	atlastin GTPase 3	-0.476	0.0192
ATP2A3	ATPase sarcoplasmic/endoplasmic reticulum Ca2+ transporting 3	0.651	0.0192
ARF4	ADP ribosylation factor 4	-0.616	0.0194
TCAF2	TRPM8 channel associated factor 2	0.617	0.0194
ARL4D	ADP ribosylation factor like GTPase 4D	-0.791	0.0194
NR4A1	nuclear receptor subfamily 4 group A member 1	0.488	0.0194
WEE1	WEE1 G2 checkpoint kinase	-0.893	0.0194
GTF2F2	general transcription factor IIF subunit 2	-0.648	0.0194
IL18RAP	interleukin 18 receptor accessory protein	0.912	0.0195
NDUFA12	NADH:ubiquinone oxidoreductase subunit A12	-0.405	0.0195
CP	ceruloplasmin	-1.619	0.0196
TRAT1	T cell receptor associated transmembrane adaptor 1	1.387	0.0196
P2RX7	purinergic receptor P2X 7	0.525	0.0196
HBG1	hemoglobin subunit gamma 1	2.651	0.0197
HBG1	hemoglobin subunit gamma 1	2.651	0.0197
GON7	GON7, KEOPS complex subunit homolog	-0.587	0.0198
C2orf76	chromosome 2 open reading frame 76	-0.506	0.0198
HELB	DNA helicase B	0.639	0.0198
HOXB7	homeobox B7	-0.561	0.0198
SSBP1	single stranded DNA binding protein 1	-0.419	0.0198
KLF7	Kruppel like factor 7	0.718	0.0199
ZNF93	zinc finger protein 93	-0.557	0.0199
MS4A8	membrane spanning 4-domains A8	-1.561	0.0199
PSMD12	proteasome 26S subunit, non-ATPase 12	-0.519	0.0199
AOX1	aldehyde oxidase 1	-1.577	0.0199
CARNMT1	carnosine N-methyltransferase 1	-0.517	0.0200
EIF4E2	eukaryotic translation initiation factor 4E family member 2	-0.579	0.0201
PBXIP1	PBX homeobox interacting protein 1	0.395	0.0201
SLC46A3	solute carrier family 46 member 3	0.707	0.0201
SFMBT2	Scm-like with four mbt domains 2	0.766	0.0201

FGF10	fibroblast growth factor 10	-1.202	0.0202
TCTA	T-cell leukemia translocation altered	-0.324	0.0202
SFN	stratifin	-0.579	0.0203
NFATC3	nuclear factor of activated T-cells 3	0.497	0.0203
IL18	interleukin 18	1.103	0.0203
FMOD	fibromodulin	1.357	0.0203
RASGRP4	RAS guanyl releasing protein 4	0.763	0.0204
DHX29	DEAH-box helicase 29	-0.507	0.0204
ADRA2A	adrenoceptor alpha 2A	-0.612	0.0205
MPPED2	metallophosphoesterase domain containing 2	-1.411	0.0205
HPSE2	heparanase 2 (inactive)	-0.852	0.0206
PDE2A	phosphodiesterase 2A	0.474	0.0206
TRMT61B	tRNA methyltransferase 61B	-0.695	0.0206
NRM	nurim (nuclear envelope membrane protein)	0.529	0.0207
FAM200B	family with sequence similarity 200 member B	-0.612	0.0207
SIRPB1	signal regulatory protein beta 1	1.193	0.0207
SPINT2	serine peptidase inhibitor, Kunitz type 2	-0.741	0.0207
RNASE2	ribonuclease A family member 2	1.272	0.0207
INTS12	integrator complex subunit 12	-0.542	0.0207
TRAF3IP3	TRAF3 interacting protein 3	1.159	0.0208
SQLE	squalene epoxidase	-0.652	0.0209
LRRFIP1	LRR binding FLII interacting protein 1	0.641	0.0209
SYTL3	synaptotagmin like 3	0.721	0.0209
MYCBP	MYC binding protein	-0.545	0.0209
S100B	S100 calcium binding protein B	0.832	0.0209
CHCHD1	coiled-coil-helix-coiled-coil-helix domain containing 1	-0.830	0.0209
ENG	endoglin	0.758	0.0209
NUDT11	nudix hydrolase 11	-0.613	0.0209
S100A9	S100 calcium binding protein A9	2.217	0.0210
RPL26L1	ribosomal protein L26 like 1	-0.548	0.0210
TXNL4A	thioredoxin like 4A	-0.661	0.0210
ATF7IP	activating transcription factor 7 interacting protein	0.431	0.0211
LRTOMT	leucine rich transmembrane and O-methyltransferase domain cor	-0.556	0.0211
CCL3	C-C motif chemokine ligand 3	1.268	0.0211
SCGB1D2	secretoglobin family 1D member 2	-2.194	0.0211
BCDIN3D	BCDIN3 domain containing RNA methyltransferase	-0.341	0.0211
STK38L	serine/threonine kinase 38 like	0.515	0.0211
PRMT9	protein arginine methyltransferase 9	-0.608	0.0211
FLJ42393	uncharacterized LOC401105	0.703	0.0212
HS3ST3B1	heparan sulfate-glucosamine 3-sulfotransferase 3B1	0.726	0.0212
DSEL	dermatan sulfate epimerase-like	-1.016	0.0212
SLC25A37	solute carrier family 25 member 37	1.546	0.0212
PLTP	phospholipid transfer protein	0.910	0.0212
BORCS7	BLOC-1 related complex subunit 7	-0.817	0.0213
MOCOS	molybdenum cofactor sulfurase	-0.929	0.0213

ZNF322	zinc finger protein 322	-0.441	0.0213
PACRGL	PARK2 coregulated like	-0.500	0.0213
FOLR2	folate receptor beta	1.165	0.0213
VTRNA1-3	vault RNA 1-3	-1.458	0.0213
MRPS2	mitochondrial ribosomal protein S2	-0.810	0.0213
NDUFA6	NADH:ubiquinone oxidoreductase subunit A6	-0.525	0.0214
VAMP2	vesicle associated membrane protein 2	0.648	0.0215
PRNP	prion protein	0.432	0.0215
QPRT	quinolinate phosphoribosyltransferase	-0.702	0.0216
CLDN7	claudin 7	-0.790	0.0216
ILDR1	immunoglobulin like domain containing receptor 1	-0.523	0.0217
ECI2	enoyl-CoA delta isomerase 2	-0.692	0.0217
SPAG1	sperm associated antigen 1	-0.493	0.0217
FGF7P6	fibroblast growth factor 7 pseudogene 6	-0.596	0.0218
PBX3	PBX homeobox 3	0.435	0.0220
RAB3IP	RAB3A interacting protein	-0.551	0.0220
PPP1R14A	protein phosphatase 1 regulatory inhibitor subunit 14A	-1.044	0.0221
GSTA4	glutathione S-transferase alpha 4	-1.006	0.0221
PLB1	phospholipase B1	0.419	0.0221
NPY6R	neuropeptide Y receptor Y6 (pseudogene)	0.494	0.0221
SNAI2	snail family transcriptional repressor 2	-1.511	0.0223
RPS6KA1	ribosomal protein S6 kinase A1	0.523	0.0223
ZNF322	zinc finger protein 322	-0.463	0.0224
SYNJ1	synaptojanin 1	0.500	0.0224
FAM124A	family with sequence similarity 124 member A	0.564	0.0224
RAB4A	RAB4A, member RAS oncogene family	-0.505	0.0224
SIRPB2	signal regulatory protein beta 2	0.711	0.0224
NBEAL1	neurobeachin like 1	-0.964	0.0225
ACSM3	acyl-CoA synthetase medium-chain family member 3	-0.892	0.0226
S100A13	S100 calcium binding protein A13	-0.800	0.0227
CCDC186	coiled-coil domain containing 186	-0.501	0.0228
GAS1	growth arrest specific 1	0.519	0.0228
LILRB5	leukocyte immunoglobulin like receptor B5	0.545	0.0228
TESPA1	thymocyte expressed, positive selection associated 1	0.979	0.0228
PDE4DIP	phosphodiesterase 4D interacting protein	0.808	0.0228
YWHAE	tyrosine 3-monooxygenase/tryptophan 5-monooxygenase activa	-0.422	0.0229
UGT2B10	UDP glucuronosyltransferase family 2 member B10	-0.491	0.0229
CRTC2	CREB regulated transcription coactivator 2	0.564	0.0229
S100A12	S100 calcium binding protein A12	2.117	0.0229
ZNF121	zinc finger protein 121	-0.375	0.0229
NUP37	nucleoporin 37	-0.740	0.0230
ICAM2	intercellular adhesion molecule 2	0.528	0.0231
CFAP44	cilia and flagella associated protein 44	-0.873	0.0231
HHLA3	HERV-H LTR-associating 3	-0.435	0.0231
FAM171B	family with sequence similarity 171 member B	-0.882	0.0231

ARAP3	ArfGAP with RhoGAP domain, ankyrin repeat and PH domain 3	0.382	0.0232
MAP4K1	mitogen-activated protein kinase kinase kinase kinase 1	0.783	0.0232
PTS	6-pyruvoyltetrahydropterin synthase	-0.790	0.0232
TMC8	transmembrane channel like 8	0.585	0.0232
MALT1	MALT1 paracaspase	0.659	0.0233
FCGRT	Fc fragment of IgG receptor and transporter	0.561	0.0233
CAPRIN1	cell cycle associated protein 1	-0.772	0.0233
NR4A3	nuclear receptor subfamily 4 group A member 3	0.683	0.0234
PCDHB14	protocadherin beta 14	-0.712	0.0234
NDUFS1	NADH:ubiquinone oxidoreductase core subunit S1	-0.711	0.0234
FAM183A	family with sequence similarity 183 member A	-1.134	0.0234
OAZ1	ornithine decarboxylase antizyme 1	0.580	0.0234
COPS2	COP9 signalosome subunit 2	-0.837	0.0234
ASRGL1	asparaginase like 1	-1.319	0.0235
FAM83B	family with sequence similarity 83 member B	-0.666	0.0235
MGC32805	uncharacterized LOC153163	-0.614	0.0235
USP15	ubiquitin specific peptidase 15	0.737	0.0235
YIF1B	Yip1 interacting factor homolog B, membrane trafficking protein	-0.390	0.0235
TMTC3	transmembrane and tetratricopeptide repeat containing 3	-0.741	0.0235
TXNL1	thioredoxin like 1	-0.454	0.0235
CYB5RL	cytochrome b5 reductase like	0.342	0.0236
RPL36A	ribosomal protein L36a	-0.681	0.0237
GAS5	growth arrest specific 5 (non-protein coding)	-0.892	0.0237
GRIPAP1	GRIP1 associated protein 1	0.478	0.0237
HK2	hexokinase 2	1.533	0.0237
NFATC1	nuclear factor of activated T-cells 1	0.566	0.0238
IFT74	intraflagellar transport 74	-0.700	0.0238
FNBP1	formin binding protein 1	0.573	0.0238
ETFA	electron transfer flavoprotein alpha subunit	-0.743	0.0238
OGFRL1	opioid growth factor receptor like 1	0.850	0.0240
PILRA	paired immunoglobin like type 2 receptor alpha	1.031	0.0240
TRIM27	tripartite motif containing 27	-0.633	0.0240
TRIM27	tripartite motif containing 27	-0.633	0.0240
TUBB4B	tubulin beta 4B class IVb	-0.463	0.0240
HIST1H4B	histone cluster 1 H4 family member b	-0.908	0.0241
ZNF799	zinc finger protein 799	-0.822	0.0241
ATP5C1	ATP synthase, H+ transporting, mitochondrial F1 complex, gamn	-0.537	0.0242
DNAL1	dynein axonemal light chain 1	-0.496	0.0243
TRIM25	tripartite motif containing 25	0.615	0.0244
LPAL2	lipoprotein(a) like 2, pseudogene	0.676	0.0244
MILR1	mast cell immunoglobulin like receptor 1	0.907	0.0244
ATPIF1	ATPase inhibitory factor 1	-0.404	0.0245
HIBCH	3-hydroxyisobutyryl-CoA hydrolase	-0.820	0.0245
HIVEP3	human immunodeficiency virus type I enhancer binding protein [illegible]	0.371	0.0245
ZNF252P	zinc finger protein 252, pseudogene	-0.462	0.0245

NR5A2	nuclear receptor subfamily 5 group A member 2	-0.697	0.0245
PKHD1L1	polycystic kidney and hepatic disease 1 (autosomal recessive)-like	-1.982	0.0245
SPCS2	signal peptidase complex subunit 2	-0.646	0.0245
KCNAB2	potassium voltage-gated channel subfamily A regulatory beta sub	0.598	0.0245
MFF	mitochondrial fission factor	-0.508	0.0245
MRPL35	mitochondrial ribosomal protein L35	-0.716	0.0246
KDR	kinase insert domain receptor	0.954	0.0246
LOC105370792	uncharacterized LOC105370792	0.801	0.0246
HOXD8	homeobox D8	-0.387	0.0247
CCND3	cyclin D3	0.573	0.0247
TBCA	tubulin folding cofactor A	-0.574	0.0247
EIF2AK2	eukaryotic translation initiation factor 2 alpha kinase 2	0.621	0.0247
TCTEX1D2	Tctex1 domain containing 2	-0.816	0.0247
GATM	glycine amidinotransferase	1.013	0.0247
PNPLA4	patatin like phospholipase domain containing 4	-0.464	0.0248
MTAP	methylthioadenosine phosphorylase	-0.415	0.0249
BET1	Bet1 golgi vesicular membrane trafficking protein	-0.680	0.0249
TGDS	TDP-glucose 4,6-dehydratase	-0.444	0.0249
RUVBL2	RuvB like AAA ATPase 2	-0.428	0.0249
SPATS2	spermatogenesis associated serine rich 2	-0.723	0.0250
RPF1	ribosome production factor 1 homolog	-0.620	0.0251
ZNF57	zinc finger protein 57	-0.690	0.0251
RCAN2	regulator of calcineurin 2	0.416	0.0251
EEF1E1	eukaryotic translation elongation factor 1 epsilon 1	-0.963	0.0253
SLA2	Src like adaptor 2	0.492	0.0253
SLC2A14	solute carrier family 2 member 14	0.881	0.0253
PRMT6	protein arginine methyltransferase 6	-0.621	0.0253
TET2	tet methylcytosine dioxygenase 2	0.778	0.0254
BNIP3L	BCL2 interacting protein 3 like	0.796	0.0254
TIMD4	T-cell immunoglobulin and mucin domain containing 4	0.960	0.0254
KMO	kynurenine 3-monooxygenase	1.085	0.0254
TARP	TCR gamma alternate reading frame protein	1.078	0.0255
LMOD1	leiomodin 1	0.928	0.0256
PRDX3	peroxiredoxin 3	-0.537	0.0256
SLC29A3	solute carrier family 29 member 3	0.612	0.0257
IGFBP6	insulin like growth factor binding protein 6	0.825	0.0257
CD1A	CD1a molecule	0.459	0.0257
USMG5	up-regulated during skeletal muscle growth 5 homolog (mouse)	-0.519	0.0258
LRPPRC	leucine rich pentatricopeptide repeat containing	-0.457	0.0258
SNORD116-14	small nucleolar RNA, C/D box 116-14	-1.159	0.0258
HCP5	HLA complex P5 (non-protein coding)	0.655	0.0258
ITGA4	integrin subunit alpha 4	0.976	0.0259
SPAG17	sperm associated antigen 17	-0.817	0.0259
ITPKB	inositol-trisphosphate 3-kinase B	0.468	0.0259
C7orf43	chromosome 7 open reading frame 43	0.455	0.0259

ANAPC11	anaphase promoting complex subunit 11	-0.654	0.0259
UGT8	UDP glycosyltransferase 8	-0.575	0.0259
FASTKD2	FAST kinase domains 2	-0.747	0.0259
SPAG6	sperm associated antigen 6	-0.892	0.0260
FAM69C	family with sequence similarity 69 member C	-0.663	0.0260
PGGHG	protein-glucosylgalactosylhydroxylysine glucosidase	0.672	0.0260
LHFPL3	lipoma HMGIC fusion partner-like 3	-1.041	0.0260
EIF5AL1	eukaryotic translation initiation factor 5A-like 1	-0.608	0.0260
PPARD	peroxisome proliferator activated receptor delta	0.669	0.0261
SERPINI2	serpin family I member 2	-0.571	0.0261
CCL7	C-C motif chemokine ligand 7	1.515	0.0261
LRRC42	leucine rich repeat containing 42	-0.330	0.0261
CD209	CD209 molecule	0.666	0.0261
MSL3	male-specific lethal 3 homolog (Drosophila)	0.524	0.0262
MDM1	Mdm1 nuclear protein	0.636	0.0262
C9orf116	chromosome 9 open reading frame 116	-0.807	0.0263
POTEJ	POTE ankyrin domain family member J	0.403	0.0265
ST3GAL4	ST3 beta-galactoside alpha-2,3-sialyltransferase 4	-0.463	0.0266
AK6	adenylate kinase 6	-0.522	0.0266
HSD17B11	hydroxysteroid 17-beta dehydrogenase 11	0.628	0.0266
CSTA	cystatin A	1.875	0.0266
WNK1	WNK lysine deficient protein kinase 1	0.604	0.0267
TTC9	tetratricopeptide repeat domain 9	-0.459	0.0267
UBASH3B	ubiquitin associated and SH3 domain containing B	0.786	0.0267
LMLN	leishmanolysin like peptidase	-0.451	0.0267
PFKFB4	6-phosphofructo-2-kinase/fructose-2,6-biphosphatase 4	0.665	0.0268
DPY19L4	dpy-19 like 4 (C. elegans)	-0.673	0.0268
STX5	syntaxin 5	-0.417	0.0268
MMP26	matrix metallopeptidase 26	-2.376	0.0268
TJP1	tight junction protein 1	-1.061	0.0268
ZNF664	zinc finger protein 664	-0.389	0.0268
TLR10	toll like receptor 10	0.955	0.0268
P2RX1	purinergic receptor P2X 1	0.602	0.0268
CSF2RB	colony stimulating factor 2 receptor beta common subunit	0.836	0.0268
UFM1	ubiquitin fold modifier 1	-0.827	0.0268
ATP6V1B2	ATPase H+ transporting V1 subunit B2	0.817	0.0268
RPS6KA3	ribosomal protein S6 kinase A3	0.768	0.0269
NRM	nurim (nuclear envelope membrane protein)	0.587	0.0269
NRM	nurim (nuclear envelope membrane protein)	0.587	0.0269
TXNDC9	thioredoxin domain containing 9	-0.894	0.0269
ZAK	sterile alpha motif and leucine zipper containing kinase AZK	-0.678	0.0270
CYSLTR2	cysteinyl leukotriene receptor 2	0.667	0.0270
GNA13	G protein subunit alpha 13	0.522	0.0270
GPR39	G protein-coupled receptor 39	0.970	0.0270
PPFIBP2	PPFIA binding protein 2	-0.707	0.0270

KCNMB3	potassium calcium-activated channel subfamily M regulatory beta	0.387	0.0270
HLA-F	major histocompatibility complex, class I, F	0.620	0.0271
SUMO1	small ubiquitin-like modifier 1	-0.411	0.0271
SEC31B	SEC31 homolog B, COPII coat complex component	0.394	0.0271
CEMP1	cementum protein 1	0.365	0.0271
LAT2	linker for activation of T-cells family member 2	0.417	0.0271
CKMT2	creatine kinase, mitochondrial 2	-0.766	0.0274
ZNF320	zinc finger protein 320	-0.852	0.0274
LINC01123	long intergenic non-protein coding RNA 1123	-1.181	0.0274
PCDH1	protocadherin 1	-0.354	0.0275
KCNN4	potassium calcium-activated channel subfamily N member 4	0.615	0.0275
CXCL11	C-X-C motif chemokine ligand 11	0.471	0.0275
SIGLEC7	sialic acid binding Ig like lectin 7	0.556	0.0276
ZNF667-AS1	ZNF667 antisense RNA 1 (head to head)	-1.011	0.0276
PLEKHA1	pleckstrin homology domain containing A1	-0.667	0.0276
ATP8B1	ATPase phospholipid transporting 8B1	-0.675	0.0277
LAMTOR5	late endosomal/lysosomal adaptor, MAPK and MTOR activator 5	-0.374	0.0278
PAN2	PAN2 poly(A) specific ribonuclease subunit	-0.428	0.0279
PIM1	Pim-1 proto-oncogene, serine/threonine kinase	0.677	0.0279
ZNF627	zinc finger protein 627	-0.594	0.0279
MRPS35	mitochondrial ribosomal protein S35	-0.648	0.0279
IRF6	interferon regulatory factor 6	-0.995	0.0279
EIF5AL1	eukaryotic translation initiation factor 5A-like 1	-0.608	0.0279
CGN	cingulin	-0.530	0.0279
TDG	thymine DNA glycosylase	-0.447	0.0280
PIGH	phosphatidylinositol glycan anchor biosynthesis class H	-0.560	0.0280
SETD9	SET domain containing 9	-0.668	0.0280
CD247	CD247 molecule	0.686	0.0281
STK33	serine/threonine kinase 33	-0.892	0.0281
NDST2	N-deacetylase and N-sulfotransferase 2	0.591	0.0281
TPRKB	TP53RK binding protein	-0.855	0.0282
TSTD1	thiosulfate sulfurtransferase like domain containing 1	-0.539	0.0282
PDLIM7	PDZ and LIM domain 7	0.599	0.0282
VPS8	VPS8, CORVET complex subunit	0.387	0.0283
C11orf57	chromosome 11 open reading frame 57	-0.520	0.0283
CHIT1	chitinase 1	1.171	0.0283
CTSV	cathepsin V	-0.833	0.0283
AKAP14	A-kinase anchoring protein 14	-0.611	0.0284
PKHD1	polycystic kidney and hepatic disease 1 (autosomal recessive)	0.578	0.0284
PDZK1IP1	PDZK1 interacting protein 1	0.652	0.0285
UBA7	ubiquitin like modifier activating enzyme 7	0.535	0.0286
PDCD2	programmed cell death 2	-0.366	0.0286
PATL1	PAT1 homolog 1, processing body mRNA decay factor	0.702	0.0286
RPS7	ribosomal protein S7	-0.783	0.0286
GLI3	GLI family zinc finger 3	-0.798	0.0287

TMEM91	transmembrane protein 91	0.433	0.0288
C1QBP	complement C1q binding protein	-0.426	0.0288
UTP11	UTP11, small subunit processome component homolog (S. cere	-0.627	0.0288
PRRG4	proline rich and Gla domain 4	-0.529	0.0288
HELZ	helicase with zinc finger	-0.776	0.0288
CFHR1	complement factor H related 1	1.214	0.0288
ATXN7L1	ataxin 7 like 1	0.937	0.0288
ZW10	zw10 kinetochore protein	-0.589	0.0288
PPP1R9B	protein phosphatase 1 regulatory subunit 9B	0.421	0.0288
PSMD6	proteasome 26S subunit, non-ATPase 6	-0.490	0.0288
ABCD3	ATP binding cassette subfamily D member 3	-0.699	0.0288
DOK5	docking protein 5	-0.569	0.0288
USF2	upstream transcription factor 2, c-fos interacting	0.542	0.0289
TNFRSF11B	TNF receptor superfamily member 11b	0.756	0.0289
NXT2	nuclear transport factor 2 like export factor 2	-0.534	0.0290
WBP1L	WW domain binding protein 1-like	0.354	0.0290
PFKFB3	6-phosphofructo-2-kinase/fructose-2,6-biphosphatase 3	0.767	0.0290
LCK	LCK proto-oncogene, Src family tyrosine kinase	0.658	0.0290
TMEM61	transmembrane protein 61	-0.402	0.0290
ARHGEF1	Rho guanine nucleotide exchange factor 1	0.618	0.0290
STAT2	signal transducer and activator of transcription 2	0.614	0.0291
BACH2	BTB domain and CNC homolog 2	0.787	0.0292
ATM	ATM serine/threonine kinase	0.569	0.0292
SEPT10	septin 10	-0.759	0.0295
PF4	platelet factor 4	1.673	0.0295
MRPL14	mitochondrial ribosomal protein L14	-0.499	0.0295
DZIP1	DAZ interacting zinc finger protein 1	-0.914	0.0295
TERC	telomerase RNA component	-0.568	0.0295
ARHGAP32	Rho GTPase activating protein 32	-0.650	0.0295
PCCB	propionyl-CoA carboxylase beta subunit	-0.517	0.0295
SNORD116-21	small nucleolar RNA, C/D box 116-21	-0.823	0.0296
ANO10	anoctamin 10	-0.710	0.0296
USO1	USO1 vesicle transport factor	-0.480	0.0297
BCL2L10	BCL2 like 10	-0.646	0.0297
IHH	indian hedgehog	0.410	0.0297
CDC42EP4	CDC42 effector protein 4	-0.316	0.0297
VIM	vimentin	0.584	0.0297
ITPRIP	inositol 1,4,5-trisphosphate receptor interacting protein	0.565	0.0297
KRT8	keratin 8	-1.146	0.0298
PLD3	phospholipase D family member 3	0.601	0.0298
CD36	CD36 molecule	-1.419	0.0298
ZNF552	zinc finger protein 552	-0.796	0.0298
ASB14	ankyrin repeat and SOCS box containing 14	-0.401	0.0299
GNAI1	G protein subunit alpha i1	-0.855	0.0299
RPL15	ribosomal protein L15	-0.468	0.0299

CCND2	cyclin D2	0.670	0.0299
ENDOG	endonuclease G	-0.365	0.0301
CFDP1	craniofacial development protein 1	-0.538	0.0301
ADRB3	adrenoceptor beta 3	0.645	0.0302
GIMAP1-GIMAP5	GIMAP1-GIMAP5 readthrough	0.660	0.0303
CCDC138	coiled-coil domain containing 138	-0.668	0.0303
PTPN1	protein tyrosine phosphatase, non-receptor type 1	0.431	0.0303
KCTD11	potassium channel tetramerization domain containing 11	0.438	0.0304
CCDC84	coiled-coil domain containing 84	0.414	0.0305
RASA3	RAS p21 protein activator 3	0.665	0.0305
PON3	paraoxonase 3	-0.474	0.0305
SMAD9	SMAD family member 9	-1.020	0.0306
DNAH3	dynein axonemal heavy chain 3	-0.365	0.0306
C1orf186	chromosome 1 open reading frame 186	-1.378	0.0307
ZNF138	zinc finger protein 138	-0.525	0.0307
ZNF229	zinc finger protein 229	-0.441	0.0308
CSNK1G1	casein kinase 1 gamma 1	-0.385	0.0309
UBE3A	ubiquitin protein ligase E3A	-0.441	0.0309
SLC15A4	solute carrier family 15 member 4	-0.745	0.0309
FCER2	Fc fragment of IgE receptor II	0.420	0.0309
PRDX5	peroxiredoxin 5	-0.822	0.0309
OLFML2B	olfactomedin like 2B	0.869	0.0309
CRHR1-IT1	CRHR1 intronic transcript 1	0.482	0.0310
RNF166	ring finger protein 166	0.679	0.0310
AK6	adenylate kinase 6	-0.581	0.0310
RRN3P2	RRN3 homolog, RNA polymerase I transcription factor pseudoge	0.639	0.0310
MUT	methylmalonyl-CoA mutase	-0.589	0.0310
LTA4H	leukotriene A4 hydrolase	0.849	0.0311
RAB30	RAB30, member RAS oncogene family	0.602	0.0311
ZNF506	zinc finger protein 506	-0.601	0.0311
IL18BP	interleukin 18 binding protein	0.689	0.0311
PLP2	proteolipid protein 2	0.554	0.0311
FAM102B	family with sequence similarity 102 member B	0.649	0.0311
RASGEF1B	RasGEF domain family member 1B	0.862	0.0312
DNAJB9	DnaJ heat shock protein family (Hsp40) member B9	-0.574	0.0312
PBOV1	prostate and breast cancer overexpressed 1	-0.631	0.0312
RPN1	ribophorin I	-0.423	0.0312
CMTR1	cap methyltransferase 1	0.351	0.0313
NAMPT	nicotinamide phosphoribosyltransferase	0.702	0.0313
CYB5A	cytochrome b5 type A	-1.081	0.0314
NOL4L	nucleolar protein 4 like	0.450	0.0315
DPEP2	dipeptidase 2	0.825	0.0315
CYP4X1	cytochrome P450 family 4 subfamily X member 1	-0.807	0.0315
MRPL30	mitochondrial ribosomal protein L30	-0.556	0.0315
CTF1	cardiotrophin 1	0.356	0.0315

MMP16	matrix metallopeptidase 16	-1.044	0.0315
ATP5H	ATP synthase, H+ transporting, mitochondrial Fo complex subur	-0.448	0.0316
ATP6V1G1	ATPase H+ transporting V1 subunit G1	-0.485	0.0317
GIT2	GIT ArfGAP 2	0.637	0.0317
TCAIM	T-cell activation inhibitor, mitochondrial	-0.457	0.0317
NDUFC1	NADH:ubiquinone oxidoreductase subunit C1	-0.539	0.0317
COL21A1	collagen type XXI alpha 1 chain	-0.409	0.0318
DNAJB5	DnaJ heat shock protein family (Hsp40) member B5	0.528	0.0318
SPN	sialophorin	0.579	0.0318
LIF	leukemia inhibitory factor	-0.711	0.0318
TTC23L	tetratricopeptide repeat domain 23 like	-0.409	0.0320
NMRK1	nicotinamide riboside kinase 1	-0.676	0.0321
TWISTNB	TWIST neighbor	-0.948	0.0321
NUP62	nucleoporin 62	0.601	0.0321
CKLF	chemokine like factor	0.461	0.0321
NSA2	NSA2, ribosome biogenesis homolog	-0.498	0.0321
KCTD3	potassium channel tetramerization domain containing 3	-0.782	0.0322
VOPP1	vesicular, overexpressed in cancer, prosurvival protein 1	0.388	0.0322
SLC15A3	solute carrier family 15 member 3	0.983	0.0322
ZBED8	zinc finger BED-type containing 8	-0.758	0.0322
TRMT13	tRNA methyltransferase 13 homolog	-0.509	0.0323
ATP5J	ATP synthase, H+ transporting, mitochondrial Fo complex subur	-0.525	0.0323
PSMA3	proteasome subunit alpha 3	-0.547	0.0323
SNORD116-19	small nucleolar RNA, C/D box 116-19	-1.026	0.0323
SNORD116-19	small nucleolar RNA, C/D box 116-19	-1.026	0.0323
OR1J4	olfactory receptor family 1 subfamily J member 4	-1.003	0.0324
HBS1L	HBS1 like translational GTPase	-0.614	0.0324
OPRPN	opiorphin prepropeptide	-1.125	0.0325
PIEZO1	piezo type mechanosensitive ion channel component 1	0.373	0.0325
TIGD6	tigger transposable element derived 6	-0.472	0.0325
SOD1	superoxide dismutase 1, soluble	-0.438	0.0325
PIGR	polymeric immunoglobulin receptor	-1.486	0.0325
BEND6	BEN domain containing 6	-0.444	0.0325
CCDC88C	coiled-coil domain containing 88C	1.401	0.0325
DYNC1I2	dynein cytoplasmic 1 intermediate chain 2	-0.566	0.0325
M6PR	mannose-6-phosphate receptor, cation dependent	0.564	0.0326
PLA2G2A	phospholipase A2 group IIA	-1.118	0.0326
NCOR2	nuclear receptor corepressor 2	0.406	0.0326
SIGLEC1	sialic acid binding Ig like lectin 1	0.719	0.0326
NHEJ1	non-homologous end joining factor 1	-0.341	0.0326
UBE2N	ubiquitin conjugating enzyme E2 N	-0.436	0.0327
DUOXA1	dual oxidase maturation factor 1	-0.671	0.0327
CHL1	cell adhesion molecule L1 like	1.370	0.0327
SMOX	spermine oxidase	0.514	0.0327
TCEB1	transcription elongation factor B subunit 1	-0.733	0.0328

HDAC10	histone deacetylase 10	0.332	0.0328
RPL26L1	ribosomal protein L26 like 1	-0.608	0.0328
STX11	syntaxin 11	0.892	0.0328
PARPBP	PARP1 binding protein	-0.575	0.0329
SLC30A5	solute carrier family 30 member 5	-0.455	0.0330
ODAM	odontogenic, ameloblast asssociated	-0.775	0.0330
CPNE8	copine 8	0.471	0.0330
KLRG1	killer cell lectin like receptor G1	0.980	0.0330
RAB21	RAB21, member RAS oncogene family	-0.515	0.0330
CHCHD2	coiled-coil-helix-coiled-coil-helix domain containing 2	-0.626	0.0332
CMPK2	cytidine/uridine monophosphate kinase 2	0.474	0.0332
CYP2S1	cytochrome P450 family 2 subfamily S member 1	0.391	0.0332
FAM69B	family with sequence similarity 69 member B	-0.336	0.0333
NFKBIA	NFKB inhibitor alpha	0.514	0.0333
UQCRFS1	ubiquinol-cytochrome c reductase, Rieske iron-sulfur polypeptide	-0.781	0.0333
SRP19	signal recognition particle 19	-0.470	0.0333
RPL7A	ribosomal protein L7a	-0.876	0.0333
RHOBTB3	Rho related BTB domain containing 3	-0.920	0.0333
SKA2	spindle and kinetochore associated complex subunit 2	-0.873	0.0333
PCCA	propionyl-CoA carboxylase alpha subunit	-0.702	0.0334
ARHGAP4	Rho GTPase activating protein 4	0.493	0.0335
PRF1	perforin 1	0.841	0.0335
MRPS30	mitochondrial ribosomal protein S30	-0.449	0.0336
EDNRB	endothelin receptor type B	-1.208	0.0336
SCFD1	sec1 family domain containing 1	-0.319	0.0337
OSTC	oligosaccharyltransferase complex non-catalytic subunit	-0.802	0.0337
GRINA	glutamate ionotropic receptor NMDA type subunit associated pro	0.629	0.0337
ATXN7L1	ataxin 7 like 1	1.103	0.0337
ZNF436	zinc finger protein 436	-0.693	0.0337
APOC2	apolipoprotein C2	0.638	0.0338
FLVCR2	feline leukemia virus subgroup C cellular receptor family member	0.540	0.0339
NDUFV2	NADH:ubiquinone oxidoreductase core subunit V2	-0.470	0.0339
C16orf87	chromosome 16 open reading frame 87	-0.569	0.0339
EGLN3	egl-9 family hypoxia inducible factor 3	0.835	0.0341
RPL34	ribosomal protein L34	-0.818	0.0341
ZBTB25	zinc finger and BTB domain containing 25	0.491	0.0341
SERPIND1	serpin family D member 1	0.547	0.0342
NUDT15	nudix hydrolase 15	-0.420	0.0343
ARID3B	AT-rich interaction domain 3B	0.424	0.0343
PCNX1	pecanex homolog 1 (Drosophila)	0.511	0.0343
PRKRA	protein activator of interferon induced protein kinase EIF2AK2	-0.388	0.0343
SHISA6	shisa family member 6	-1.283	0.0344
TRIM2	tripartite motif containing 2	-0.847	0.0344
ADH1A	alcohol dehydrogenase 1A (class I), alpha polypeptide	-0.533	0.0346
SLC16A14	solute carrier family 16 member 14	-0.514	0.0347

ALG6	ALG6, alpha-1,3-glucosyltransferase	-0.418	0.0347
BMP8A	bone morphogenetic protein 8a	-0.548	0.0348
B3GNT2	UDP-GlcNAc:betaGal beta-1,3-N-acetylglucosaminyltransferase 2	-0.498	0.0349
VAMP1	vesicle associated membrane protein 1	0.512	0.0350
BTNL8	butyrophilin like 8	0.465	0.0350
PGM3	phosphoglucomutase 3	-0.683	0.0350
LOC100130691	uncharacterized LOC100130691	0.399	0.0352
AVPR1B	arginine vasopressin receptor 1B	-0.530	0.0352
SEC23B	Sec23 homolog B, coat complex II component	-0.456	0.0353
ZNF333	zinc finger protein 333	0.280	0.0353
CEBPZ	CCAAT/enhancer binding protein zeta	-0.531	0.0353
SNX27	sorting nexin family member 27	0.496	0.0353
SH3BGRL2	SH3 domain binding glutamate rich protein like 2	-0.704	0.0353
TPM4	tropomyosin 4	0.577	0.0353
COX7A2L	cytochrome c oxidase subunit 7A2 like	-0.432	0.0354
TNFRSF10C	TNF receptor superfamily member 10c	0.575	0.0354
HVCN1	hydrogen voltage gated channel 1	0.531	0.0354
THAP5	THAP domain containing 5	-0.485	0.0354
LDB2	LIM domain binding 2	-0.961	0.0354
CCDC93	coiled-coil domain containing 93	0.478	0.0354
NOD1	nucleotide binding oligomerization domain containing 1	0.500	0.0354
KDF1	keratinocyte differentiation factor 1	-0.385	0.0354
SNORD116-20	small nucleolar RNA, C/D box 116-20	-0.928	0.0354
ACTB	actin beta	0.580	0.0354
SRP54	signal recognition particle 54	-0.484	0.0356
CMBL	carboxymethylenebutenolidase homolog	-0.766	0.0356
TAP2	transporter 2, ATP binding cassette subfamily B member	0.643	0.0356
TAP2	transporter 2, ATP binding cassette subfamily B member	0.643	0.0356
TAP2	transporter 2, ATP binding cassette subfamily B member	0.643	0.0356
CDC42EP3	CDC42 effector protein 3	0.705	0.0357
ECHS1	enoyl-CoA hydratase, short chain 1	-0.511	0.0357
NDUFB11	NADH:ubiquinone oxidoreductase subunit B11	-0.421	0.0358
DISC1	disrupted in schizophrenia 1	0.311	0.0359
ACTN1	actinin alpha 1	0.733	0.0362
POTEF	POTE ankyrin domain family member F	0.411	0.0363
MYBL1	MYB proto-oncogene like 1	1.096	0.0363
PLAC9	placenta specific 9	-0.651	0.0363
LAX1	lymphocyte transmembrane adaptor 1	0.561	0.0363
NDFIP2	Nedd4 family interacting protein 2	-0.833	0.0363
SNORA22	small nucleolar RNA, H/ACA box 22	-1.209	0.0363
RAD17	RAD17 checkpoint clamp loader component	-0.385	0.0364
CD8A	CD8a molecule	0.449	0.0365
MYC	v-myc avian myelocytomatosis viral oncogene homolog	-0.767	0.0365
SRRM1	serine and arginine repetitive matrix 1	0.367	0.0365
ANGEL1	angel homolog 1	0.350	0.0366

NFKBID	NFKB inhibitor delta	0.489	0.0366
CDH20	cadherin 20	0.530	0.0368
SAR1B	secretion associated Ras related GTPase 1B	-0.575	0.0368
TMEM173	transmembrane protein 173	0.448	0.0369
CXCL2	C-X-C motif chemokine ligand 2	1.231	0.0370
TRIM66	tripartite motif containing 66	0.353	0.0370
HEMGN	hemogen	2.339	0.0370
GHRL	ghrelin and obestatin prepropeptide	0.534	0.0370
MRPS18A	mitochondrial ribosomal protein S18A	-0.638	0.0372
ARID3B	AT-rich interaction domain 3B	0.511	0.0372
PIBF1	progesterone immunomodulatory binding factor 1	-0.795	0.0372
NAGPA	N-acetylglucosamine-1-phosphodiester alpha-N-acetylglucosami	0.348	0.0372
NDUFS4	NADH:ubiquinone oxidoreductase subunit S4	-0.468	0.0372
ARHGAP45	Rho GTPase activating protein 45	0.505	0.0373
PPP2R2C	protein phosphatase 2 regulatory subunit Bgamma	-0.493	0.0373
PUS3	pseudouridylate synthase 3	-0.545	0.0373
CYP2J2	cytochrome P450 family 2 subfamily J member 2	-0.810	0.0373
RALGAPA2	Ral GTPase activating protein catalytic alpha subunit 2	-0.923	0.0373
C6orf120	chromosome 6 open reading frame 120	-0.347	0.0375
ORMDL2	ORMDL sphingolipid biosynthesis regulator 2	-0.577	0.0375
NNAT	neuronatin	0.383	0.0375
GFPT1	glutamine--fructose-6-phosphate transaminase 1	-0.807	0.0375
SEMA3E	semaphorin 3E	-0.535	0.0376
CEP170	centrosomal protein 170	0.550	0.0376
CBR1	carbonyl reductase 1	-0.454	0.0376
STAT5B	signal transducer and activator of transcription 5B	0.606	0.0376
EPHB2	EPH receptor B2	0.517	0.0376
R3HCC1L	R3H domain and coiled-coil containing 1 like	0.379	0.0377
CLEC12A	C-type lectin domain family 12 member A	1.107	0.0377
PLAU	plasminogen activator, urokinase	0.683	0.0378
KLHL26	kelch like family member 26	0.390	0.0378
COX14	COX14, cytochrome c oxidase assembly factor	-0.404	0.0378
IRF1	interferon regulatory factor 1	0.594	0.0378
ODF2L	outer dense fiber of sperm tails 2 like	-0.730	0.0378
PCGF5	polycomb group ring finger 5	0.527	0.0378
UGT2B15	UDP glucuronosyltransferase family 2 member B15	-1.570	0.0378
UNC13B	unc-13 homolog B	-0.514	0.0378
ORM1	orosomucoid 1	-0.905	0.0378
ETV6	ETS variant 6	0.685	0.0378
GEMIN6	gem nuclear organelle associated protein 6	-0.729	0.0378
FOLR3	folate receptor 3	0.658	0.0380
PCSK1	proprotein convertase subtilisin/kexin type 1	0.629	0.0380
BMPR1A	bone morphogenetic protein receptor type 1A	-0.960	0.0380
POC1B	POC1 centriolar protein B	-0.593	0.0380
CEP44	centrosomal protein 44	-0.748	0.0381

F3	coagulation factor III, tissue factor	0.966	0.0381
CCDC107	coiled-coil domain containing 107	0.344	0.0381
TBC1D8B	TBC1 domain family member 8B	-0.521	0.0381
RMDN2	regulator of microtubule dynamics 2	-0.628	0.0381
MAP3K8	mitogen-activated protein kinase kinase kinase 8	0.483	0.0382
RNASEK	ribonuclease K	0.396	0.0383
WDR63	WD repeat domain 63	-0.723	0.0383
AMPD2	adenosine monophosphate deaminase 2	0.482	0.0385
C9orf85	chromosome 9 open reading frame 85	-0.817	0.0386
MB21D2	Mab-21 domain containing 2	0.845	0.0386
RGS18	regulator of G-protein signaling 18	1.813	0.0386
DAP	death associated protein	-0.350	0.0386
MBP	myelin basic protein	0.489	0.0386
NUDT7	nudix hydrolase 7	0.425	0.0387
SASH3	SAM and SH3 domain containing 3	0.431	0.0387
SUCLG1	succinate-CoA ligase alpha subunit	-0.449	0.0387
SKA2	spindle and kinetochore associated complex subunit 2	-0.620	0.0388
NDUFB2	NADH:ubiquinone oxidoreductase subunit B2	-0.405	0.0389
TUSC3	tumor suppressor candidate 3	-0.791	0.0389
PRKAR2B	protein kinase cAMP-dependent type II regulatory subunit beta	0.861	0.0389
HLA-DOA	major histocompatibility complex, class II, DO alpha	0.599	0.0389
SSR2	signal sequence receptor subunit 2	-0.515	0.0392
DPP6	dipeptidyl peptidase like 6	0.773	0.0392
CPSF3L	cleavage and polyadenylation specific factor 3-like	-0.295	0.0393
FAM189A2	family with sequence similarity 189 member A2	-0.887	0.0394
ADORA3	adenosine A3 receptor	0.779	0.0394
GGA3	golgi associated, gamma adaptin ear containing, ARF binding pro	0.465	0.0396
PCNP	PEST proteolytic signal containing nuclear protein	-0.506	0.0396
ZNF682	zinc finger protein 682	-0.728	0.0396
FILIP1L	filamin A interacting protein 1 like	0.799	0.0396
KCND3	potassium voltage-gated channel subfamily D member 3	-0.519	0.0396
CD1D	CD1d molecule	0.351	0.0396
MBIP	MAP3K12 binding inhibitory protein 1	-0.556	0.0397
FOXN2	forkhead box N2	0.432	0.0397
ARHGEF12	Rho guanine nucleotide exchange factor 12	-0.707	0.0397
GRHL1	grainyhead like transcription factor 1	-0.421	0.0397
COL8A1	collagen type VIII alpha 1 chain	0.553	0.0397
DNAJC10	DnaJ heat shock protein family (Hsp40) member C10	-0.707	0.0397
MUC13	mucin 13, cell surface associated	-0.967	0.0398
AMD1	adenosylmethionine decarboxylase 1	-0.762	0.0399
CDKL4	cyclin dependent kinase like 4	-0.496	0.0399
SIGLEC10	sialic acid binding Ig like lectin 10	0.525	0.0400
NDUFC2-KCTD14	NDUFC2-KCTD14 readthrough	-0.791	0.0401
CMTM2	CKLF like MARVEL transmembrane domain containing 2	0.661	0.0401
DNAH12	dynein axonemal heavy chain 12	-0.613	0.0401

ATP2B2	ATPase plasma membrane Ca2+ transporting 2	-0.358	0.0401
LINC00173	long intergenic non-protein coding RNA 173	0.606	0.0405
PGS1	phosphatidylglycerophosphate synthase 1	0.367	0.0405
WDR49	WD repeat domain 49	-0.645	0.0405
MRPL24	mitochondrial ribosomal protein L24	-0.624	0.0405
DES	desmin	0.677	0.0407
DCAF13	DDB1 and CUL4 associated factor 13	-0.499	0.0407
SLC22A5	solute carrier family 22 member 5	-0.545	0.0407
FZD5	frizzled class receptor 5	-0.408	0.0407
CD96	CD96 molecule	0.996	0.0407
LMAN2	lectin, mannose binding 2	-0.501	0.0407
RNF111	ring finger protein 111	0.496	0.0408
FAM81B	family with sequence similarity 81 member B	-0.740	0.0408
MED17	mediator complex subunit 17	-0.374	0.0409
MIR223	microRNA 223	1.578	0.0409
ENPP3	ectonucleotide pyrophosphatase/phosphodiesterase 3	-2.157	0.0410
CYP26B1	cytochrome P450 family 26 subfamily B member 1	0.464	0.0410
GVINP1	GTPase, very large interferon inducible pseudogene 1	0.880	0.0410
LY9	lymphocyte antigen 9	0.780	0.0410
TIMM8B	translocase of inner mitochondrial membrane 8 homolog B	-0.736	0.0411
LOC284379	solute carrier family 7 member 3 pseudogene	0.483	0.0411
IL4R	interleukin 4 receptor	0.642	0.0411
FCRL5	Fc receptor like 5	0.682	0.0411
CECR2	CECR2, histone acetyl-lysine reader	-0.540	0.0412
RSPH4A	radial spoke head 4 homolog A	-0.700	0.0412
PLPPR4	phospholipid phosphatase related 4	-0.713	0.0412
ADAMTS2	ADAM metallopeptidase with thrombospondin type 1 motif 2	0.659	0.0412
SNORD56B	small nucleolar RNA, C/D box 56B	1.128	0.0412
COPG1	coatomer protein complex subunit gamma 1	-0.512	0.0412
COX17	COX17, cytochrome c oxidase copper chaperone	-0.526	0.0412
MRPS31	mitochondrial ribosomal protein S31	-0.541	0.0413
NMNAT1	nicotinamide nucleotide adenylyltransferase 1	-0.439	0.0414
MAX	MYC associated factor X	0.495	0.0414
HGF	hepatocyte growth factor	-0.679	0.0418
DLG5	discs large MAGUK scaffold protein 5	-0.992	0.0418
GTF2A2	general transcription factor IIA subunit 2	-0.608	0.0420
ZNF43	zinc finger protein 43	-0.509	0.0420
FNBP1L	formin binding protein 1 like	-1.105	0.0420
MUC3A	mucin 3A, cell surface associated	0.454	0.0421
STT3A	STT3A, catalytic subunit of the oligosaccharyltransferase comple›	-0.594	0.0421
MAN2A2	mannosidase alpha class 2A member 2	0.497	0.0421
TMEM99	transmembrane protein 99	-0.568	0.0422
ALOX12P2	arachidonate 12-lipoxygenase pseudogene 2	0.327	0.0423
STK40	serine/threonine kinase 40	0.473	0.0423
CYP1B1	cytochrome P450 family 1 subfamily B member 1	-0.760	0.0423

TCEB2	transcription elongation factor B subunit 2	-0.756	0.0423
REG1A	regenerating family member 1 alpha	0.520	0.0423
DPP7	dipeptidyl peptidase 7	0.374	0.0423
BTNL8	butyrophilin like 8	0.445	0.0423
ICAM3	intercellular adhesion molecule 3	0.777	0.0423
SLC9A2	solute carrier family 9 member A2	-0.547	0.0423
ANO6	anoctamin 6	0.498	0.0423
HNRNPL	heterogeneous nuclear ribonucleoprotein L	-0.410	0.0423
ATP11A	ATPase phospholipid transporting 11A	-0.635	0.0423
LILRA3	leukocyte immunoglobulin like receptor A3	0.841	0.0423
SNRNP25	small nuclear ribonucleoprotein U11/U12 subunit 25	-0.612	0.0423
CFHR1	complement factor H related 1	0.699	0.0423
POTEM	POTE ankyrin domain family member M	0.450	0.0425
ATP5D	ATP synthase, H+ transporting, mitochondrial F1 complex, delta	-0.278	0.0426
P2RY13	purinergic receptor P2Y13	1.538	0.0426
TAF1D	TATA-box binding protein associated factor, RNA polymerase I su	-0.627	0.0427
USP32	ubiquitin specific peptidase 32	0.417	0.0427
GALNT1	polypeptide N-acetylgalactosaminyltransferase 1	-0.413	0.0427
CXCR2	C-X-C motif chemokine receptor 2	2.011	0.0427
PRKAA1	protein kinase AMP-activated catalytic subunit alpha 1	-0.359	0.0427
SDCBP2	syndecan binding protein 2	-0.365	0.0427
MAP9	microtubule associated protein 9	-0.519	0.0427
RNF103	ring finger protein 103	-0.529	0.0427
GAB2	GRB2 associated binding protein 2	0.514	0.0428
VEGFA	vascular endothelial growth factor A	0.754	0.0428
LINC00299	long intergenic non-protein coding RNA 299	0.627	0.0428
LDLRAD4	low density lipoprotein receptor class A domain containing 4	0.365	0.0429
ZNF480	zinc finger protein 480	-0.395	0.0430
JUP	junction plakoglobin	-0.565	0.0430
CALCRL	calcitonin receptor like receptor	-1.354	0.0430
TMEM63C	transmembrane protein 63C	-0.465	0.0430
ARHGAP24	Rho GTPase activating protein 24	0.784	0.0431
SNORD36B	small nucleolar RNA, C/D box 36B	-0.818	0.0433
GZMB	granzyme B	0.888	0.0433
MPC2	mitochondrial pyruvate carrier 2	-0.770	0.0433
NFU1	NFU1 iron-sulfur cluster scaffold	-0.447	0.0433
PFDN4	prefoldin subunit 4	-1.214	0.0433
ZFAND1	zinc finger AN1-type containing 1	-0.772	0.0434
DNAH12	dynein axonemal heavy chain 12	-0.755	0.0434
ZBTB7A	zinc finger and BTB domain containing 7A	0.301	0.0435
SLC34A2	solute carrier family 34 member 2	-1.446	0.0435
ALAS2	5'-aminolevulinate synthase 2	1.173	0.0435
SEMA4A	semaphorin 4A	0.509	0.0435
TBC1D22A	TBC1 domain family member 22A	0.458	0.0436
CIPC	CLOCK interacting pacemaker	-0.413	0.0436

ZC3HAV1L	zinc finger CCCH-type containing, antiviral 1 like	-0.413	0.0436
PNPLA6	patatin like phospholipase domain containing 6	0.450	0.0437
HS6ST3	heparan sulfate 6-O-sulfotransferase 3	-0.488	0.0437
ACAT1	acetyl-CoA acetyltransferase 1	-0.393	0.0437
MAP2K6	mitogen-activated protein kinase kinase 6	-1.237	0.0437
RGS20	regulator of G-protein signaling 20	-0.554	0.0437
NME2	NME/NM23 nucleoside diphosphate kinase 2	-0.692	0.0438
COA6	cytochrome c oxidase assembly factor 6	-0.522	0.0438
TCEAL8	transcription elongation factor A like 8	-0.392	0.0438
HNF1B	HNF1 homeobox B	-1.195	0.0438
RBMS1	RNA binding motif single stranded interacting protein 1	0.422	0.0438
KLF5	Kruppel like factor 5	-0.645	0.0439
LAPTM4B	lysosomal protein transmembrane 4 beta	-0.882	0.0439
C14orf166	chromosome 14 open reading frame 166	-0.363	0.0439
GLS	glutaminase	0.461	0.0439
CFAP43	cilia and flagella associated protein 43	-0.995	0.0440
PAK1	p21 (RAC1) activated kinase 1	0.670	0.0440
SCOC	short coiled-coil protein	-0.846	0.0440
PEX13	peroxisomal biogenesis factor 13	-0.330	0.0440
SMCHD1	structural maintenance of chromosomes flexible hinge domain cc	0.545	0.0440
HSD17B10	hydroxysteroid 17-beta dehydrogenase 10	-0.407	0.0441
DAAM1	dishevelled associated activator of morphogenesis 1	-0.580	0.0441
BCAR1	BCAR1, Cas family scaffolding protein	-0.371	0.0441
RHBDL2	rhomboid like 2	-0.445	0.0441
JAK3	Janus kinase 3	0.479	0.0441
TSGA10	testis specific 10	-0.675	0.0441
GALM	galactose mutarotase	0.729	0.0441
CCDC90B	coiled-coil domain containing 90B	-0.416	0.0442
EIF2B3	eukaryotic translation initiation factor 2B subunit gamma	-0.581	0.0442
GNA14	G protein subunit alpha 14	-0.409	0.0442
INCA1	inhibitor of CDK, cyclin A1 interacting protein 1	0.399	0.0442
SURF4	surfeit 4	-0.461	0.0442
GPRC5C	G protein-coupled receptor class C group 5 member C	-0.441	0.0444
GMIP	GEM interacting protein	0.702	0.0444
ZNHIT3	zinc finger HIT-type containing 3	-0.486	0.0446
SUSD1	sushi domain containing 1	0.406	0.0448
BTBD3	BTB domain containing 3	-1.189	0.0448
HOXB5	homeobox B5	-0.635	0.0448
DLGAP1	DLG associated protein 1	-0.873	0.0448
CUL3	cullin 3	-0.467	0.0448
B4GALNT1	beta-1,4-N-acetyl-galactosaminyltransferase 1	0.464	0.0449
ERI2	ERI1 exoribonuclease family member 2	-0.467	0.0450
TARP	TCR gamma alternate reading frame protein	1.176	0.0450
TARP	TCR gamma alternate reading frame protein	1.176	0.0450
FGFBP1	fibroblast growth factor binding protein 1	1.027	0.0450

GATSL3	GATS protein like 3	-0.585	0.0450
KRCC1	lysine rich coiled-coil 1	-0.650	0.0450
CCDC125	coiled-coil domain containing 125	-0.363	0.0451
TEFM	transcription elongation factor, mitochondrial	-0.655	0.0453
USB1	U6 snRNA biogenesis phosphodiesterase 1	0.374	0.0453
TMEM86A	transmembrane protein 86A	0.535	0.0453
IRAK3	interleukin 1 receptor associated kinase 3	1.230	0.0453
POPDC3	popeye domain containing 3	-0.612	0.0453
TGFBR2	transforming growth factor beta receptor 2	0.508	0.0454
MRPL47	mitochondrial ribosomal protein L47	-0.541	0.0455
PANK1	pantothenate kinase 1	-0.293	0.0457
KCNA3	potassium voltage-gated channel subfamily A member 3	0.975	0.0457
ITGB6	integrin subunit beta 6	1.171	0.0457
ABHD2	abhydrolase domain containing 2	0.470	0.0457
DUOX1	dual oxidase 1	-0.955	0.0458
OGFR	opioid growth factor receptor	0.341	0.0458
IARS2	isoleucyl-tRNA synthetase 2, mitochondrial	-0.432	0.0458
IL5RA	interleukin 5 receptor subunit alpha	-0.651	0.0458
ECHDC1	ethylmalonyl-CoA decarboxylase 1	-0.325	0.0458
NOL11	nucleolar protein 11	-0.382	0.0458
CYP2C19	cytochrome P450 family 2 subfamily C member 19	-0.497	0.0458
MET	MET proto-oncogene, receptor tyrosine kinase	-1.082	0.0459
SMYD2	SET and MYND domain containing 2	-0.402	0.0460
PLA1A	phospholipase A1 member A	0.432	0.0460
ZNF678	zinc finger protein 678	-0.365	0.0460
KMT2A	lysine methyltransferase 2A	0.414	0.0460
TNIP1	TNFAIP3 interacting protein 1	0.469	0.0460
MAP3K19	mitogen-activated protein kinase kinase kinase 19	-0.589	0.0460
ZNF542P	zinc finger protein 542, pseudogene	-0.403	0.0460
LINC00612	long intergenic non-protein coding RNA 612	0.407	0.0460
NXF1	nuclear RNA export factor 1	0.482	0.0461
NOL10	nucleolar protein 10	-0.517	0.0462
TRIM50	tripartite motif containing 50	0.435	0.0462
NCEH1	neutral cholesterol ester hydrolase 1	0.674	0.0463
CPT1A	carnitine palmitoyltransferase 1A	-0.725	0.0463
F2R	coagulation factor II thrombin receptor	1.065	0.0464
TACC1	transforming acidic coiled-coil containing protein 1	0.588	0.0464
YTHDF1	YTH N6-methyladenosine RNA binding protein 1	-0.335	0.0464
ZCCHC9	zinc finger CCHC-type containing 9	-0.405	0.0465
TARP	TCR gamma alternate reading frame protein	0.991	0.0465
HCG27	HLA complex group 27 (non-protein coding)	0.527	0.0465
RHOG	ras homolog family member G	0.604	0.0465
NQO2	NAD(P)H quinone dehydrogenase 2	0.628	0.0465
SDHB	succinate dehydrogenase complex iron sulfur subunit B	-0.434	0.0468
BRIX1	BRX1, biogenesis of ribosomes	-0.399	0.0468

SSX2IP	SSX family member 2 interacting protein	-0.476	0.0469
SRP72	signal recognition particle 72	-0.381	0.0470
RPP38	ribonuclease P/MRP subunit p38	-0.346	0.0470
HIST1H2BC	histone cluster 1 H2B family member c	-0.641	0.0470
ECE1	endothelin converting enzyme 1	0.673	0.0471
APAF1	apoptotic peptidase activating factor 1	0.493	0.0472
DUT	deoxyuridine triphosphatase	-0.575	0.0472
NUS1	NUS1 dehydrodolichyl diphosphate synthase subunit	-0.380	0.0472
NT5C3B	5'-nucleotidase, cytosolic IIIB	-0.501	0.0473
LINC01123	long intergenic non-protein coding RNA 1123	-0.732	0.0475
DNAJC12	DnaJ heat shock protein family (Hsp40) member C12	-0.727	0.0475
ZSWIM3	zinc finger SWIM-type containing 3	-0.423	0.0476
C8orf4	chromosome 8 open reading frame 4	-1.194	0.0476
CSF2RA	colony stimulating factor 2 receptor alpha subunit	0.931	0.0476
CSF2RA	colony stimulating factor 2 receptor alpha subunit	0.931	0.0476
ARPC1B	actin related protein 2/3 complex subunit 1B	0.549	0.0476
IL16	interleukin 16	0.422	0.0476
RGS5	regulator of G-protein signaling 5	-0.969	0.0476
TAS2R40	taste 2 receptor member 40	0.763	0.0476
PCMTD1	protein-L-isoaspartate (D-aspartate) O-methyltransferase domain	-0.568	0.0476
TAX1BP1	Tax1 binding protein 1	-0.361	0.0476
GRAMD1B	GRAM domain containing 1B	0.699	0.0476
CETN3	centrin 3	-0.701	0.0476
APPL1	adaptor protein, phosphotyrosine interacting with PH domain an	-0.412	0.0476
SPCS2	signal peptidase complex subunit 2	-0.603	0.0476
PPP2CA	protein phosphatase 2 catalytic subunit alpha	-0.370	0.0476
FKBP4	FK506 binding protein 4	-0.514	0.0478
TEX30	testis expressed 30	-0.758	0.0479
HIF1A	hypoxia inducible factor 1 alpha subunit	-0.515	0.0479
UQCC3	ubiquinol-cytochrome c reductase complex assembly factor 3	-0.317	0.0479
PBX2	PBX homeobox 2	0.503	0.0479
ERO1B	endoplasmic reticulum oxidoreductase 1 beta	0.401	0.0479
ATP2C2	ATPase secretory pathway Ca2+ transporting 2	-0.772	0.0479
TM9SF2	transmembrane 9 superfamily member 2	-0.346	0.0480
UBASH3A	ubiquitin associated and SH3 domain containing A	0.533	0.0482
RWDD4	RWD domain containing 4	-0.387	0.0482
BNC2	basonuclin 2	0.649	0.0482
APOBEC3A_B	APOBEC3A and APOBEC3B deletion hybrid	0.796	0.0482
FCAR	Fc fragment of IgA receptor	0.377	0.0482
FOXN3	forkhead box N3	0.471	0.0482
DCAF6	DDB1 and CUL4 associated factor 6	-0.348	0.0483
RAP2C	RAP2C, member of RAS oncogene family	0.504	0.0483
RGS4	regulator of G-protein signaling 4	-0.860	0.0483
PPP1R42	protein phosphatase 1 regulatory subunit 42	-0.560	0.0484
MAGI2	membrane associated guanylate kinase, WW and PDZ domain cc	-0.389	0.0486

IFIT5	interferon induced protein with tetratricopeptide repeats 5	0.473	0.0486
ZNF680	zinc finger protein 680	-0.467	0.0488
CTSH	cathepsin H	0.614	0.0488
MYL12B	myosin light chain 12B	-0.459	0.0488
NDUFAF1	NADH:ubiquinone oxidoreductase complex assembly factor 1	-0.514	0.0488
COA5	cytochrome c oxidase assembly factor 5	-0.509	0.0491
HLA-F-AS1	HLA-F antisense RNA 1	0.357	0.0491
KRT14	keratin 14	0.668	0.0491
GTF2H2C_2	GTF2H2 family member C, copy 2	-0.668	0.0491
GTF2H2C_2	GTF2H2 family member C, copy 2	-0.668	0.0491
CLDN15	claudin 15	-0.479	0.0491
ZFP36L2	ZFP36 ring finger protein like 2	0.374	0.0491
KNG1	kininogen 1	-0.357	0.0491
CCRL2	C-C motif chemokine receptor like 2	0.747	0.0491
SAP25	Sin3A associated protein 25	0.454	0.0491
NIFK	nucleolar protein interacting with the FHA domain of MKI67	-0.393	0.0491
EFHB	EF-hand domain family member B	-0.494	0.0492
CMIP	c-Maf inducing protein	0.557	0.0492
MRVI1-AS1	MRVI1 antisense RNA 1	0.397	0.0492
CTAGE15	CTAGE family member 15	-0.994	0.0492
SHKBP1	SH3KBP1 binding protein 1	0.377	0.0492
TIMP4	TIMP metallopeptidase inhibitor 4	0.379	0.0493
HOMER2	homer scaffolding protein 2	-1.285	0.0493
MSX2	msh homeobox 2	-0.691	0.0494
CYP2C18	cytochrome P450 family 2 subfamily C member 18	-0.395	0.0494
LY75-CD302	LY75-CD302 readthrough	0.801	0.0494
TMED2	transmembrane p24 trafficking protein 2	-0.497	0.0494
CCT2	chaperonin containing TCP1 subunit 2	-0.491	0.0494
BTN2A1	butyrophilin subfamily 2 member A1	0.434	0.0496
ZNF718	zinc finger protein 718	-0.587	0.0496
ZNF718	zinc finger protein 718	-0.587	0.0496
AAK1	AP2 associated kinase 1	0.528	0.0496
SPG21	spastic paraplegia 21 (autosomal recessive, Mast syndrome)	0.327	0.0496
CNN3	calponin 3	-0.692	0.0498
SCNN1A	sodium channel epithelial 1 alpha subunit	-1.077	0.0499

Supplemental Table 2 List of pathways with altered expression in LNG-IUD users compared to controls, including only pathways with a combined score of ≥5.0.

Term	Overlap	P-value	Z-score	Combined Score
Immune System	343/1547	2.91E-34	-2.232	172.369
Cytokine Signaling in Immune system	144/620	2.15E-16	-2.387	86.110
Interferon Signaling	62/196	7.82E-14	-2.070	62.461
Interferon gamma signaling	39/93	1.40E-13	-1.735	51.351
Interferon alpha/beta signaling	26/68	1.65E-08	-1.729	30.985
Signaling by Interleukins	77/392	3.21E-06	-2.249	28.444
Regulation of signaling by CBL	9/18	7.35E-05	-0.989	9.415
Interleukin-3, 5 and GM-CSF signaling	46/261	2.99E-03	-1.837	10.675
Interleukin-2 signaling	44/252	4.33E-03	-1.765	9.603
Innate Immune System	172/807	1.51E-15	-2.404	82.049
Toll-Like Receptors Cascades	38/140	4.45E-07	-1.967	28.774
Toll Like Receptor 4 (TLR4) Cascade	30/122	5.51E-05	-1.759	17.245
MyD88:Mal cascade initiated on plasma membrane	21/92	1.89E-03	-1.445	9.063
MyD88-independent TLR3/TLR4 cascade	20/97	8.04E-03	-1.221	5.890
TRIF-mediated TLR3/TLR4 signaling	20/97	8.04E-03	-1.204	5.806
Activated TLR4 signalling	25/112	1.04E-03	-1.550	10.644
Trafficking and processing of endosomal TLR	8/13	2.61E-05	-0.650	6.865
Toll Like Receptor 3 (TLR3) Cascade	20/97	8.04E-03	-1.212	5.845
Toll Like Receptor 2 (TLR2) Cascade	21/92	1.89E-03	-1.433	8.987
Toll Like Receptor TLR1:TLR2 Cascade	21/92	1.89E-03	-1.427	8.949
Toll Like Receptor TLR6:TLR2 Cascade	21/92	1.89E-03	-1.416	8.877
Fcgamma receptor (FCGR) dependent phagocytosis	30/120	3.95E-05	-1.637	16.596
Regulation of actin dynamics for phagocytic cup formation	21/97	3.72E-03	-1.237	6.919
Fc epsilon receptor (FCERI) signaling	65/395	2.86E-03	-1.882	11.021
Nucleotide-binding domain, leucine rich repeat containing receptor (NLR) signaling pathways	16/47	5.05E-05	-1.594	15.774
Inflammasomes	10/17	4.24E-06	-1.161	14.364
DAP12 interactions	59/359	4.46E-03	-1.783	9.652
DAP12 signaling	55/344	1.02E-02	-1.454	6.671
C-type lectin receptors (CLRs)	27/123	8.81E-04	-1.669	11.737
Adaptive Immune System	153/762	8.64E-12	-2.215	56.432
Immunoregulatory interactions between a Lymphoid and a non-Lymphoid cell	51/157	4.04E-12	-1.954	51.262
TCR signaling	33/118	1.20E-06	-1.870	25.497
Translocation of ZAP-70 to Immunological synapse	12/21	6.86E-07	-1.480	21.007
Phosphorylation of CD3 and TCR zeta chains	15/24	4.75E-09	-1.841	35.279
Generation of second messenger molecules	20/35	1.23E-10	-1.894	43.216
Downstream TCR signaling	22/99	2.15E-03	-1.481	9.097
Costimulation by the CD28 family	27/72	1.43E-08	-1.854	33.476
PD-1 signaling	15/26	2.23E-08	-1.816	32.001
CD28 co-stimulation	11/33	9.17E-04	-1.326	9.275
CD28 dependent Vav1 pathway	8/12	1.12E-05	-0.882	10.058
CTLA4 inhibitory signaling	8/22	2.45E-03	-0.885	5.321

Antigen processing-Cross presentation	19/82	2.53E-03	-1.430	8.550
Antigen activates B Cell Receptor (BCR) leading to generation of second messengers	13/47	2.27E-03	-1.132	6.890
Hemostasis	138/552	1.27E-18	-2.140	88.196
Platelet activation, signaling and aggregation	73/253	9.94E-14	-2.120	63.481
GPVI-mediated activation cascade	26/53	2.15E-11	-1.740	42.745
Response to elevated platelet cytosolic Ca2+	28/110	4.99E-05	-1.604	15.886
Platelet degranulation	27/105	5.58E-05	-1.592	15.586
Platelet Aggregation (Plug Formation)	11/37	2.61E-03	-0.961	5.721
Cell surface interactions at the vascular wall	35/101	1.31E-09	-1.855	37.940
PECAM1 interactions	8/12	1.12E-05	-0.841	9.586
Platelet homeostasis	23/87	1.23E-04	-1.667	15.009
Metabolism	299/1908	3.08E-08	-2.113	36.551
The citric acid (TCA) cycle and respiratory electron transport	41/153	2.32E-07	-1.806	27.588
Respiratory electron transport, ATP synthesis by chemiosmotic coupling, and heat production by uncoupling proteins	34/109	4.62E-08	-1.854	31.319
Respiratory electron transport	29/88	1.17E-07	-1.804	28.792
Complex I biogenesis	17/49	2.23E-05	-1.637	17.530
Signal regulatory protein (SIRP) family interactions	10/13	9.74E-08	-1.362	21.988
Metabolism of carbohydrates	48/282	4.95E-03	-1.495	7.933
Arachidonic acid metabolism	14/53	2.51E-03	-1.194	7.149
Signal Transduction	340/2465	4.21E-04	-1.750	13.604
Chemokine receptors bind chemokines	20/56	2.57E-06	-1.679	21.614
Signaling by Rho GTPases	68/367	8.04E-05	-1.819	17.151
Rho GTPase cycle	32/122	7.69E-06	-1.569	18.475
Signaling by SCF-KIT	55/325	3.16E-03	-1.839	10.591
Regulation of KIT signaling	8/16	1.88E-04	-0.754	6.469
Signaling by VEGF	54/328	6.08E-03	-1.593	8.131
VEGFA-VEGFR2 Pathway	53/320	5.78E-03	-1.642	8.465
Signaling by PDGF	58/364	9.12E-03	-1.510	7.093
Integrin alphaIIb beta3 signaling	10/27	6.08E-04	-1.156	8.561
Peptide ligand-binding receptors	36/193	3.11E-03	-1.378	7.953
G-protein beta:gamma signalling	18/51	9.80E-06	-1.557	17.953
G beta:gamma signalling through PI3Kgamma	16/48	6.75E-05	-1.450	13.924
G alpha (12/13) signalling events	18/75	2.15E-03	-1.245	7.646
Gastrin-CREB signalling pathway via PKC and MAPK	67/432	9.90E-03	-1.459	6.733
G alpha (i) signalling events	41/240	8.34E-03	-1.275	6.105

Extracellular matrix organization	48/283	5.30E-03	-1.392	7.291
Integrin cell surface interactions	22/67	4.12E-06	-1.717	21.287
Extracellular matrix organization	48/283	*5.30E-03*	*-1.392*	*7.291*
Cell-Cell communication	26/131	4.75E-03	-1.290	6.903
Semaphorin interactions	19/67	1.73E-04	-1.422	12.323
Axon guidance	79/515	7.21E-03	-1.469	7.245
Response to metal ions	9/11	1.80E-07	-1.030	15.993
Metallothioneins bind metals	9/11	1.80E-07	-1.076	16.714
Mitochondrial translation	23/90	2.13E-04	-1.643	13.893
Mitochondrial translation initiation	23/84	6.84E-05	-1.642	15.751
Mitochondrial translation elongation	23/84	6.84E-05	-1.676	16.078
Mitochondrial translation termination	23/84	6.84E-05	-1.662	15.941

Supplemental Table 3. Differentially expressed genes: combined oral contraceptive users versus controls

SYMBOL	GENENAME	log2FC	adj.P.Val
MT2A	metallothionein 2A	-2.404	7.33E-05
MT1M	metallothionein 1M	-3.467	7.33E-05
MT2A	metallothionein 2A	-2.198	9.04E-05
MT1JP	metallothionein 1J, pseudogene	-2.030	0.0002
MT1A	metallothionein 1A	-2.232	0.0002
S100P	S100 calcium binding protein P	-3.685	0.0002
MT1F	metallothionein 1F	-2.747	0.0003
MT1X	metallothionein 1X	-2.371	0.0003
DHCR24	24-dehydrocholesterol reductase	-1.716	0.0003
MT1L	metallothionein 1L (gene/pseudogene)	-3.102	0.0003
MT1HL1	metallothionein 1H-like 1	-1.798	0.0003
ATRNL1	attractin like 1	1.713	0.0003
MT1G	metallothionein 1G	-3.451	0.0003
MT1B	metallothionein 1B	-1.394	0.0003
SLC30A2	solute carrier family 30 member 2	-1.450	0.0008
MT2A	metallothionein 2A	-1.604	0.0010
MFSD4A	major facilitator superfamily domain containing 4A	-1.938	0.0016
MT1H	metallothionein 1H	-3.206	0.0023
MT1E	metallothionein 1E	-2.243	0.0024
AIMP1	aminoacyl tRNA synthetase complex interacting multifunctional protein 1	-1.801	0.0024
FABP5	fatty acid binding protein 5	-1.497	0.0024
FABP5	fatty acid binding protein 5	-1.521	0.0024
SLC5A1	solute carrier family 5 member 1	-1.797	0.0025
PLXNC1	plexin C1	1.353	0.0025
SFN	stratifin	-0.995	0.0026
CATSPERB	cation channel sperm associated auxiliary subunit beta	-2.608	0.0029

AVPR1A	arginine vasopressin receptor 1A	1.407	0.0031
ANXA2	annexin A2	-1.079	0.0031
TMEM154	transmembrane protein 154	-2.000	0.0036
STEAP1	STEAP family member 1	-1.471	0.0039
BNC2	basonuclin 2	1.362	0.0046
PLA2G2A	phospholipase A2 group IIA	-2.489	0.0047
DEPDC1B	DEP domain containing 1B	-1.692	0.0055
LURAP1L	leucine rich adaptor protein 1 like	-1.026	0.0063
ESR1	estrogen receptor 1	1.401	0.0065
OTUB2	OTU deubiquitinase, ubiquitin aldehyde binding 2	-0.776	0.0077
TRIM5	tripartite motif containing 5	0.731	0.0088
TC2N	tandem C2 domains, nuclear	-1.493	0.0088
L3MBTL3	l(3)mbt-like 3 (Drosophila)	0.828	0.0111
GPX1	glutathione peroxidase 1	-1.019	0.0127
ZNF750	zinc finger protein 750	-1.564	0.0128
CAPZA2	capping actin protein of muscle Z-line alpha subunit 2	-1.309	0.0129
MMP7	matrix metallopeptidase 7	3.066	0.0134
DPP6	dipeptidyl peptidase like 6	1.668	0.0141
LPCAT2	lysophosphatidylcholine acyltransferase 2	1.134	0.0141
CPED1	cadherin like and PC-esterase domain containing 1	1.230	0.0141
PRELP	proline and arginine rich end leucine rich repeat protein	0.801	0.0141
ACACB	acetyl-CoA carboxylase beta	0.558	0.0147
FXYD3	FXYD domain containing ion transport regulator 3	-1.294	0.0147
RHPN2	rhophilin Rho GTPase binding protein 2	-1.396	0.0147
FAM177A1	family with sequence similarity 177 member A1	-1.259	0.0149
SPTLC3	serine palmitoyltransferase long chain base subunit 3	-1.543	0.0152

GAST	gastrin	-2.730	0.0194
MTHFS	5,10-methenyltetrahydrofolate synthetase (5-formyltetrahydrofolate cyclo-ligase)	-0.945	0.0200
BCAS1	breast carcinoma amplified sequence 1	-0.824	0.0200
AXIN2	axin 2	0.592	0.0200
C1orf168	chromosome 1 open reading frame 168	1.968	0.0200
FAM84B	family with sequence similarity 84 member B	-1.579	0.0200
GLRX	glutaredoxin	-1.718	0.0200
DYNLT3	dynein light chain Tctex-type 3	-1.656	0.0200
SULT1C4	sulfotransferase family 1C member 4	0.891	0.0200
PHLPP1	PH domain and leucine rich repeat protein phosphatase 1	-0.825	0.0200
C14orf37	chromosome 14 open reading frame 37	0.854	0.0200
ENPP4	ectonucleotide pyrophosphatase/phosphodiesterase 4 (putative)	-0.819	0.0208
MYH11	myosin heavy chain 11	1.377	0.0209
B3GNT2	UDP-GlcNAc:betaGal beta-1,3-N-acetylglucosaminyltransferase 2	-0.727	0.0215
OPRPN	opiorphin prepropeptide	-1.709	0.0217
GCLM	glutamate-cysteine ligase modifier subunit	-0.793	0.0218
MMP11	matrix metallopeptidase 11	1.108	0.0218
DEFB1	defensin beta 1	-1.428	0.0218
FAM129B	family with sequence similarity 129 member B	-0.724	0.0218
EIF4E3	eukaryotic translation initiation factor 4E family member 3	-0.960	0.0229
RIMKLB	ribosomal modification protein rimK like family member B	-1.798	0.0230
ST20	suppressor of tumorigenicity 20	-1.424	0.0233
ABHD17C	abhydrolase domain containing 17C	-0.657	0.0233
PHC1	polyhomeotic homolog 1	0.681	0.0233
GCNT3	glucosaminyl (N-acetyl) transferase 3, mucin type	-1.296	0.0238

FTH1	ferritin heavy chain 1	-0.844	0.0241
PLA2G16	phospholipase A2 group XVI	-1.207	0.0241
NQO1	NAD(P)H quinone dehydrogenase 1	-1.294	0.0254
PHC1	polyhomeotic homolog 1	0.685	0.0273
PDGFC	platelet derived growth factor C	1.204	0.0276
HMOX2	heme oxygenase 2	-0.539	0.0276
HEY1	hes related family bHLH transcription factor with YRPW motif 1	-1.264	0.0276
ZNF713	zinc finger protein 713	0.854	0.0276
AP1S3	adaptor related protein complex 1 sigma 3 subunit	-1.037	0.0291
SGIP1	SH3 domain GRB2 like endophilin interacting protein 1	1.910	0.0293
RHOU	ras homolog family member U	-0.732	0.0297
ZNF827	zinc finger protein 827	0.876	0.0297
PRELID3B	PRELI domain containing 3B	-0.492	0.0297
PALLD	palladin, cytoskeletal associated protein	1.020	0.0298
RPF2	ribosome production factor 2 homolog	-1.043	0.0313
PLXDC2	plexin domain containing 2	0.744	0.0318
TLR3	toll like receptor 3	-0.978	0.0318
STARD13	StAR related lipid transfer domain containing 13	0.959	0.0318
CNTN4	contactin 4	1.697	0.0318
FAM162A	family with sequence similarity 162 member A	-0.664	0.0318
LARP1B	La ribonucleoprotein domain family member 1B	-0.709	0.0329
OR52H1	olfactory receptor family 52 subfamily H member 1	0.730	0.0335
FBXL7	F-box and leucine rich repeat protein 7	0.649	0.0336
LRRC49	leucine rich repeat containing 49	0.723	0.0345
CDYL2	chromodomain Y-like 2	-1.002	0.0364
HEBP2	heme binding protein 2	-0.632	0.0364
CLUH	clustered mitochondria homolog	-0.522	0.0364

C19orf33	chromosome 19 open reading frame 33	-1.097	0.0367
PHYHIPL	phytanoyl-CoA 2-hydroxylase interacting protein like	-2.113	0.0367
TMEM45B	transmembrane protein 45B	-1.274	0.0374
DCTPP1	dCTP pyrophosphatase 1	-0.898	0.0379
ZNF704	zinc finger protein 704	0.852	0.0382
CLDN4	claudin 4	-0.915	0.0382
GPC4	glypican 4	-0.909	0.0382
FGF7	fibroblast growth factor 7	-1.993	0.0382
KRT19	keratin 19	-1.156	0.0387
PRKCA	protein kinase C alpha	1.292	0.0389
A4GNT	alpha-1,4-N-acetylglucosaminyltransferase	-0.719	0.0398
LDHA	lactate dehydrogenase A	-0.782	0.0399
CXCL14	C-X-C motif chemokine ligand 14	-2.036	0.0421
PTP4A1	protein tyrosine phosphatase type IVA, member 1	-0.939	0.0424
CD55	CD55 molecule (Cromer blood group)	-1.122	0.0436
WDR73	WD repeat domain 73	0.575	0.0450
TWF1	twinfilin actin binding protein 1	-0.566	0.0450
GUSBP1	glucuronidase, beta pseudogene 1	0.995	0.0450
MID1IP1	MID1 interacting protein 1	-0.628	0.0450
MALL	mal, T-cell differentiation protein like	-0.989	0.0458
EPB41L4B	erythrocyte membrane protein band 4.1 like 4B	-0.694	0.0458
SGK1	serum/glucocorticoid regulated kinase 1	-1.690	0.0464
CHODL	chondrolectin	-1.104	0.0478
TWF1	twinfilin actin binding protein 1	-0.613	0.0493
NIPSNAP3A	nipsnap homolog 3A	-1.023	0.0493
PRSS12	protease, serine 12	1.392	0.0493
DCHS1	dachsous cadherin-related 1	0.763	0.0493
ZDHHC17	zinc finger DHHC-type containing 17	0.652	0.0494

MRPL13	mitochondrial ribosomal protein L13	-0.961	0.0497

Supplemental Table 4 List of pathways with altered expression in COC users compared to controls, including only pathways with a combined score of ≥4.0.

Term	Overlap	P-value	Z-score	Combined Score
Response to metal ions	9/11	7.365E-19	-1.802	75.228
Metallothioneins bind metals	9/11	7.365E-19	-1.848	77.154
Metabolism	31/1908	8.919E-07	-2.229	31.051
Glycerophospholipid biosynthesis	3/102	2.779E-02	-1.761	6.308
Acyl chain remodelling of PC	3/25	5.313E-04	-1.882	14.191
Acyl chain remodelling of PI	2/16	4.599E-03	-1.863	10.028
Acyl chain remodelling of PS	2/17	5.190E-03	-1.730	9.103
Acyl chain remodelling of PE	2/23	9.417E-03	-1.792	8.359
Synthesis and interconversion of nucleotide di- and triphosphates	2/25	1.107E-02	-1.942	8.746
Keratan sulfate/keratin metabolism	2/32	1.778E-02	-1.987	8.007
Keratan sulfate biosynthesis	2/27	1.285E-02	-1.888	8.221
Metabolism of nucleotides	3/87	1.838E-02	-1.922	7.682
O-linked glycosylation	3/110	3.368E-02	-1.842	6.246
O-linked glycosylation of mucins	3/66	8.738E-03	-2.022	9.585
Glycosaminoglycan metabolism	3/121	4.275E-02	-1.723	5.433
Metabolism of lipids and lipoproteins	8/659	6.132E-02	-1.855	5.178
Disease	8/725	9.401E-02	-1.730	4.090
Diseases associated with glycosaminoglycan metabolism	2/26	1.195E-02	-1.899	8.409
Activation of Matrix Metalloproteinases	2/32	1.778E-02	-1.833	7.385
Collagen degradation	2/39	2.581E-02	-1.940	7.093
Signaling by Rho GTPases	6/367	3.081E-02	-2.068	7.197
Rho GTPase cycle	3/122	4.363E-02	-1.577	4.938
RHO GTPases activate PKNs	2/60	5.655E-02	-1.514	4.350
Smooth Muscle Contraction	2/33	1.885E-02	-1.725	6.850
Iron uptake and transport	2/43	3.094E-02	-1.858	6.459
Ca2+ pathway	2/61	5.823E-02	-1.729	4.915
Phospholipase C-mediated cascade; FGFR2	2/49	3.931E-02	-1.739	5.628
